# The Metabolomic Characterization of Different Types of Coronary Atherosclerotic Heart Disease in Male

**DOI:** 10.1155/2022/6491129

**Published:** 2022-07-12

**Authors:** Yuxuan Fan, Xianglan Quan, Shengquan Liu, Le Yue, Jizong Jiang, Zhiqing Fan

**Affiliations:** ^1^Department of Internal Medicine and Rehabilitation Science, Tohoku University, Sendai-shi, Japan; ^2^Daqing Medical College, Daqing, China; ^3^Department of Cardiology, Daqing Oilfield General Hospital, Daqing, China; ^4^School of Medicine, Shanghai University, Shanghai, China

## Abstract

**Background:**

In clinical practice, many patients with coronary atherosclerotic heart disease (CAD) have atypical clinical symptoms. It is difficult to accurately identify stable CAD or unstable CAD early through clinical symptoms and coronary angiography. This study aimed to screen the potential metabolite biomarkers in male patients with stable CAD and unstable CAD.

**Methods:**

In this work, the metabolomic characterization of the male patients with healthy control (*n* = 42), stable coronary artery disease (*n* = 60), non-ST-elevation acute coronary syndrome (*n* = 45), including prepercutaneous corona intervention (*n* = 14), and postpercutaneous coronary intervention (*n* = 31) were performed by using ultra-performance liquid chromatography-mass spectrometry (UPLC-MS). The serum samples of patients were analyzed by multivariate statistics.

**Results:**

Results showed that 17 altered metabolites were identified to have a clear distinction between the stable CAD group and the healthy subjects. Compared with the stable coronary artery disease group, 15 specific metabolite markers were found in the acute coronary syndrome group. The percutaneous coronary intervention also affected the metabolic behavior of patients with CAD.

**Conclusions:**

In summary, CAD is closely related to energy metabolism, lipid metabolism, and amino acid metabolism disorders. The different metabolic pattern characteristics of healthy, stable coronary artery disease and acute coronary syndrome are constructed, which brings a novel theoretical basis for the early diagnosis of patients with stable and unstable CAD.

## 1. Introduction

Cardiovascular disease is the leading cause of death and disability worldwide. The mortality rate of Chinese cardiovascular disease was the highest [1]. Coronary atherosclerotic heart disease (CAD) is still the cardiovascular disease with the highest fatality rate in the world [2]. At present, the “gold standard” for identifying and diagnosing CAD depends on coronary angiography and coronary CT imaging [3, 4]. Some studies indicated that the incidence of acute coronary events in CAD patients was closely related to the stability of coronary plaques and vulnerable plaques (unstable plaques) [5]. Stable coronary artery disease (SCAD) and acute coronary syndromes (ACS) (such as unstable angina, non-ST-elevation myocardial infarction, and ST-elevation myocardial infarction) are significantly different in terms of the treatment process, strategies, and prognostic outcomes. ST-segment elevation acute myocardial infarction can be diagnosed by ECG combined with clinical symptoms, while non-ST-segment elevation ACS is often difficult to distinguish between ECG, clinical symptoms, and SCAD. Although increased troponin is specific for identifying myocardial injury in non-ST-elevation ACS, it is negative in the early stage of non-ST-elevation ACS. As there is no effective conventional technology for the early diagnosis of stable coronary plaques and vulnerable plaques, it is particularly important to find a simple, low-cost, and effective method.

As a new branch of systems biology, metabolomics is an analysis technique that can quantitatively and qualitatively study the relationship between metabolites and pathological changes in the body. It can analyze the overall endogenous metabolites in cells, tissues, and other biological samples, such as blood or urine [6, 7]. Metabolomics research has unique application advantages [8–10]: (1) small changes in gene and protein expression can be amplified on metabolites by catalytic reactions of metabolic enzymes, thus making detection and analysis easier. (2) In addition to genome changes, metabolites are also affected by environmental factors and intestinal flora, which are more dynamic and more sensitive to changes in organisms. (3) Metabolic reactions and metabolic products are similar in the biological systems of all species. Therefore, the metabolomics methodology is more universal. (4) Metabolomics technology can directly detect almost all sample types, including whole blood, plasma/serum, tissue, cell, cell culture supernatant, urine, feces, food, saliva, cerebrospinal fluid, and fat, without establishing whole genome sequencing and mass expression sequence database. Applications of metabolic profiling in coronary heart disease have been developed by using LC-MS or GC-MS. The relationship between circulating blood metabolite levels and coronary heart disease was detected by metabolomics. This technology reveals different potential pathways for the development of coronary heart disease. The occurrence of cardiovascular diseases is associated with the metabolites of amino acids, lipids, peptides, carbohydrates, nucleotides, and xenobiotics [11–13]. These biomarkers are important not only for risk stratification and treatment decision-making but also for improving the understanding of the cardiovascular disease. Metabolomics research is likely to become a new technology and method for the early identification of CAD profiles. As the proinflammatory mediators do not appear to be directly linked to the disease [14], the metabolic markers open up a new diagnosis and treatment target for CAD [15, 16].

Previous studies using metabolomics as a potential diagnostic criterion for SCAD and ACS in human samples are limited, especially in China. In this work, we used metabolomics methods to construct the characteristics of patients' metabolites with SCAD, ACS, and healthy subjects. The pattern characteristics of different conditions were discussed. In addition, we analyzed the influence of PCI on the metabolites of patients with CAD. Through the differential changes and metabolic characteristics, metabolomics is expected to become a novel technology for the early diagnosis of different types of CAD.

## 2. Method

### 2.1. Baseline Characteristics and Study Design of Patients

Male participants with ages 40 to 65-year-old were enrolled in the Department of Cardiology (the Daqing Oilfield General Hospital, Daqing, China) between January 2015 and December 2015. As it was not clear that metabolites were the same in different genders under certain conditions, subjects of the same sex were selected to reduce the bias of the results. The inclusion criteria of healthy controls (HCs), SCAD, and non-ST-elevation ACS were confirmed according to American and European guidelines for the diagnosis and treatment of stable coronary heart disease and guidelines for the management of non-ST-segment elevation acute coronary syndrome in ESC [17, 18]. The subjects with no clinical history of the disease, normal electrocardiogram examination, and no uncomfortable symptoms of heavy physical activity were clinically diagnosed as healthy controls. All subjects were excluded from diseases such as hypertension, diabetes, chronic kidney disease, metabolic syndrome, heart failure, COPD, bronchial asthma, connective tissue disease, rheumatic immune disease, tumor, hyperthyroidism, hepatitis, metabolic disease, blood system disease, and severe liver and kidney damage. The baseline characteristics (including urea, Cr, Na, K, blood sugar, blood lipid, smoking history, and BMI) of patients were shown in Table 1. There were no statistical differences in the above indicators among the subjects in experimental groups.

**Figure 1:**
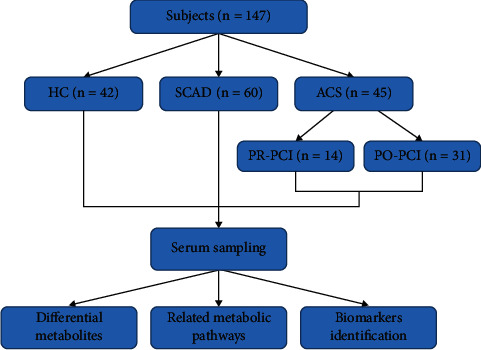
Study design. This study, involving 147 subjects, included 42 healthy controls, 105 patients with SCAD, and 45 patients with ACS. ACS: acute coronary syndrome; HC: healthy control; SCAD: stable coronary atherosclerosis disease; PR-ACS: prepercutaneous coronary intervention; PO-ACS: postpercutaneous coronary intervention.

The selected controls were healthy with no declared history of CAD (*n* = 42), SCAD (*n* = 60), and ACS group (*n* = 45), respectively. The ACS group was divided into prepercutaneous coronary intervention (PCI) (within 4 h, *n* = 14, PR-ACS group) and post-PCI (within 4 h, *n* = 31, PO-ACS group). The study was performed under the guidance of an institutional ethical committee from Daqing Oilfield General Hospital following the Helsinki Declaration. All subjects agreed to participate in this study, including the blood sample collection. The study design was shown in Figure 1.

### 2.2. Sample Preparation

Cubital vein blood samples were collected and immediately underwent plasma isolation. The blood samples were centrifuged at 1000 *g* for 10 min at room temperature. 100 *μ*L of serum was precipitated by adding 300 *μ*L of methanol and vortexed for 30 s. The precipitated proteins were then removed by centrifugation (13,000 *g*, 15 min) at 4°C. The supernatant was transferred to a microcentrifuge tube and stored at −80°C for further LC-MS analysis. Quality control (QC) samples were prepared by mixing 10 *μ*L of each sample.

### 2.3. LC-MS/MS Analysis

The separation was performed on an Agilent®1290 Infinity II (Agilent Technologies Inc., USA) using a Waters ACQUITY HSS T3 C18 (100 × 2.1 mm, 1.8 *µ*m). The column oven and the flow rate were set at 30°C and 0.5 mL/min, respectively. In positive mode, the mobile phase contained 0.1% FA in water (A) and 0.1% FA in ACN (B). In negative mode, the mobile phase consisted of 0.5 mM NH_4_F in water (A) and ACN (B). The gradient was 0 min, 1% B; 1 min, 1% B; 8 min, 100% B; 10 min, 100% B; 10.1 min, 1% B; 12 min, 1% B.

ABSCIEX® TripleTOF 6600 Plus ultra-performance liquid chromatography-tandem mass spectrometer (UPLC-Q-TOF/MS) was used to acquire the MS/MS spectra on an information-dependent basis during the LC/MS experiment. It was operated in positive and negative mode ion mode under the following operating parameters: GS1: 40 psi; GS2: 80 psi; CUR: 25 psi; TEM: 650°C; ISVF : 5000V (POS), −4000V(NEG), DP : 60V, CE: 35 ± 15. The pooled QC represented the sample matrix and metabolite composition of the samples. QC was used to construct the calibration curves and to judge precision. Stability and recovery were within the acceptable range. Acquisition software (Analyst TF1.7 software) continuously evaluated full scan survey MS data (m/z 50–1200) as it collected and triggered the acquisition of MS/MS spectra depending on preselected criteria.

### 2.4. Statistical Analysis

Overall normalization method was employed in this data analysis. The three-dimensional data, including the peak number, sample name, and normalized peak area were analyzed by the SIMCA14.0 software package (Umetrics, Umea, Sweden) for orthogonal projections to latent structures-discriminate analysis (OPLS-DA). To refine this analysis, the first principal component of variable importance projection (VIP) was obtained. The VIP value exceeding 1.0 was first selected as changed metabolites. Results were presented as mean ± SD. An unpaired, two-tailed Student's *t*-test was used for comparisons between two groups. All analyses were performed using GraphPad Prism 6.0. Differences were considered significant with *p* < 0.05.

## 3. Results

### 3.1. LC-MS Data Analysis

A total of 147 samples and 20 QC samples were obtained, of which 4568 peaks were detected for positive mode and 3516 peaks for negative mode. In order to optimize the data, the substance with RSD > 30% of the quality control samples was deleted. Data with a single group of null values or all groups with a null ≤50% were retained. The area normalization method was used to standardize the data. After processing the data, it remained at 925 peaks and 727 peaks, respectively.

### 3.2. Distinguished Health and CAD Patients by OPLS-DA Analysis

**Figure 2:**
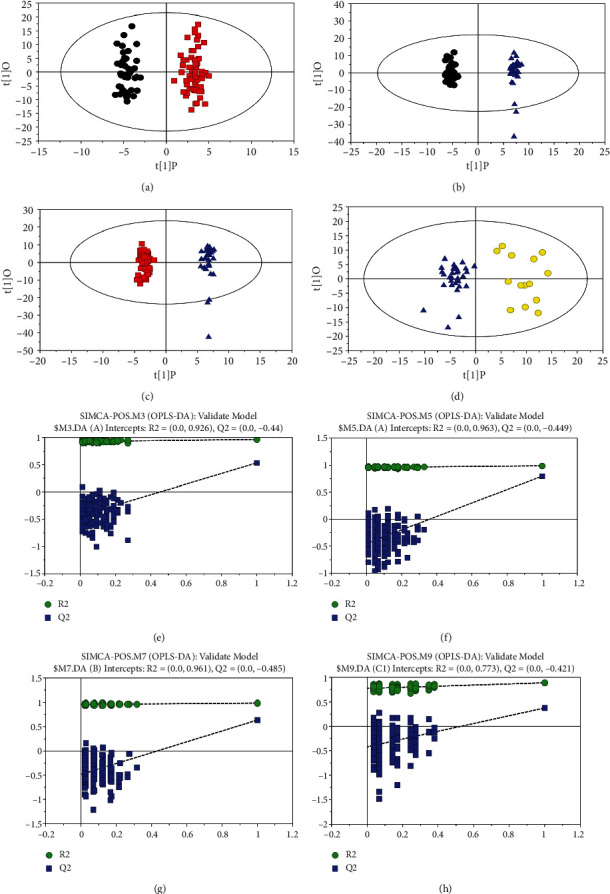
Score plot of and validated model of OPLS-DA obtained from experimental groups (POS). 2(a)–2(d). Score plot of OPLS-DA model obtained from experimental groups. black: HC, red: SCAD, blue: PR-ACS, yellow: PO-ACS. 2(e)–2(h), the validated model of OPLS-DA. 200 times were performed, and the resulting *R*^2^ and *Q*^2^ values were plotted. Green triangle: *R*^2^; blue square: *Q*^2^. The green line represents the regression line for *R*^2^ and the blue line for *Q*^2^.

SIMCA software was used to perform OPLS-DA to maximize the differences of the predictive component. The score plot of OPLS-DA(POS) was shown in Figures 2(a)–2(d). HC group was all located to the left of the midline, while the SCAD group was all located to the right (Figure 2(a)). At the latitude of the first principal component, the two groups were well separated. Compared with the principal component score, the separation trend of the two groups was obvious. The samples were all within the 99% confidence interval (Hotelling T2 Ellipse). Similar results were obtained between HC and PR-ACS, SCAD and PR-ACS, PR-ACS, and PO-ACS, respectively, as shown in Figures 2(b)–2(d). The robustness of OPLS-DA was assessed by 200 times permutation tests. The validated model of OPLS-DA was shown in Figures 2(e)–2(h). The *R*^2^ and *Q*^2^ were 0.926 and −0.44 for HC versus SCAD; 0.963 and −0.449 for HC versus PR-ACS; 0.961 and −0.485 for SCAD versus PR-ACS; and 0.773 and −0.421 for PR-ACS versus PO-ACS, respectively. It implied the validation of these OPLS-DA models. The score plot of OPLS-DA(NEG) exhibited similar results as shown in Figures S1(a)–S1(h).

### 3.3. Differential Diagnosis of Metabolic Biomarkers

**Figure 3:**
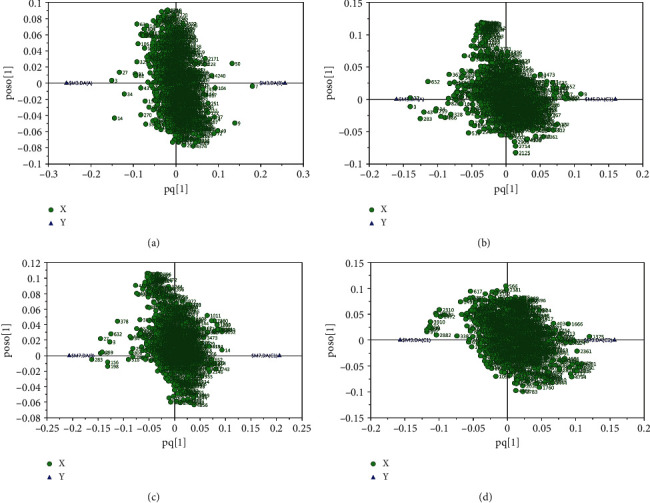
Loading plot of OPLS-DA model obtained from experimental groups (POS). (a) HC versus SCAD, (b) HC versus PR-ACS, (c) SCAD versus PR-ACS, and (d) PR-ACS versus PO-ACS.

Metabolic biomarkers can provide further information on the metabolic mechanism and biochemical pathway of disease [19, 20]. Therefore, screening for differential markers is an important step in metabolomics analysis. The loading plot of the OPLS-DA model (POS) was shown in Figure 3. The load diagram reflects the weight of the variable in the principal component. The substances on the left and right sides of the load diagram are the potential altered biomarkers. Results showed that there were specific metabolism biomarkers for health subjects versus CAD patients and SCAD versus ACS. PCI also influenced the metabolites of CAD patients. The mode of loading plot of the OPLS-DA model (NEG) obtained from experimental groups was shown in Figures S2(a)–S2(d).

To evaluate the criteria of metabolomics-based biomarkers, the variable importance in the projection value (VIP) > 1 of the OPLS-DA model and the *p* value <0.05 adjusted Student's *t*-test (*t*-test) were both used to find differential expression of metabolites. In order to identify these metabolites, we further matched the fragments of these metabolites in the MS/MS spectra. The details of metabolic parameters were shown in Table 2. As shown in Table 2, there are 17 altered metabolic biomarkers with a high correlation for HC versus CAD, 15 for SCAD versus PR-ACS, and 7 for PR-ACS versus PO-ACS (POS and NEG).

For identifying the altered metabolites, it is necessary to search the metabolomics database to find the spectrum peak attribution of the possible biomarkers. The KEGG database (https://www.genome.jp/kegg/) was used to screen all the metabolic pathways related to comparison groups. The disturbed metabolic pathways were shown in Supplementary Material 1 based on the KEGG pathway database. Compared with healthy control, we found that the levels of specific metabolites, such as 5-Cholesten-3*β*, 25(S)-diol, N-Acetyl-lysine, tyramine, biliverdin, urocanate, phenol, hypoxanthine, L-tryptophane, L-palmitoylcarnitine, were upregulated while the levels of pantetheine, indole, and lecithin were downregulated in CAD patients. Compared with SCAD patients, the levels of *α*-d-glucose, glycol-cholate, *α*-tocopherol, inosine, hypoxanthine, L-ornithine, and 5-oxoproline were upregulated in ACS patients. The levels of lecithin were downregulated. Compared with PR-PCI patients, the levels of methacrylyl-CoA, proline, 5-oxoproline, L-proline, primary bile acids, glycine, cholate, adrenosterone, and 1-oleoyl-sn-glycerol 3-phosphate were upregulated and the levels of PE (22 : 5/0 : 0) and bilirubin were downregulated in PO-PCI patients.

### 3.4. Metabolic Pathway Analysis

**Figure 4:**
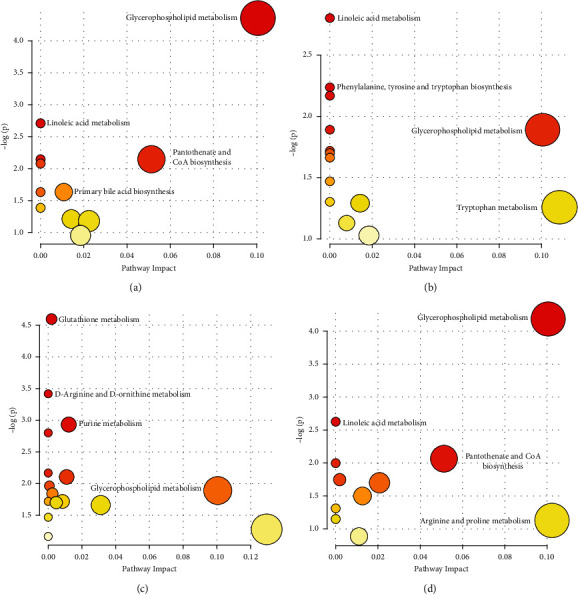
Pathway analysis of experiment group (POS). The larger the circle meant the greater the influence of topology analysis; the redder the color meant the smaller the *p* value, and vice versa. (a) HC versus SCAD, (b) HC versus PR-ACS, (c) SCAD versus PR-ACS, and (d) PR-ACS versus PO-ACS.

MetaboAnalyst 3.0 (https://www.metaboanalyst.ca) performs both metabolic pathway enrichment and topological analysis of different metabolites [21, 22]. The changes in metabolic behavior of different experimental groups in the positive mode were shown in Figures 4(a)–4(d). Compared with HC and SCAD, the metabolisms of glycerophospholipid, linoleic acid, pantothenate, CoA, and primary bile acid biosynthesis were changed. For HC versus PR-ACS, linoleic acid, phenylalanine, tyrosine, and tryptophan biosynthesis, glycerophospholipid were changed. Glutathione, D-arginine, D-ornithine, purine, and glycerophospholipid exhibited different metabolism behaviors compared to SCAD with PR-ACS. For PR-ACS versus PO-ACS, the same metabolic differences of HC versus SCAD in glycerophospholipid, linoleic acid, pantothenate, and CoA were found. Different from HC versus SCAD, arginine proline metabolism was markedly changed compared to PR-ACS with PO-ACS. The metabolic behavior changes of different groups in the negative mode were shown in Figures S3(a)–S3(d). In brief, for HC versus SCAD, the changes in metabolism were histidine, pyrimidine, tyrosine, porphyrin, and chlorophyll; for HC versus PR-ACS, linoleic acid, alpha-linolenic acid, glycerophospholipid, and pyrimidine; for SCAD versus PR-ACS, linoleic acid, alpha-linolenic acid, glycerophospholipid, and arachidonic acid; and for PR-ACS versus PO-ACS, linoleic acid, primary bile acid, fatty acid elongation, and glycerophospholipid. Changes in glycerophospholipid metabolism were found almost in all the comparison groups which suggested that glycerophospholipid had a significant impact on CAD.

## 4. Discussion

The metabolic biomarkers of CAD have been reported in many studies [21]. However, using metabolomics for the early diagnosis of CAD in terms of both stable and unstable plaques is limited, especially in Chinese. The majority of these studies paid more attention to the lipids metabolites [11, 12]. Many altered metabolites with different chemical structures were not presented. In this work, we screened all the metabolites in the experimental human groups using a metabolomics approach in an unbiased way. The altered metabolites were selected by OPLS-DA which could reduce the false positives in the data. The identified metabolites were matched with published literature and online resources. The differentiated metabolites of healthy subjects and stable and unstable CAD patients were compared. Furthermore, we focused not only on the metabolism differences between healthy and CAD patients but also on the SCAD and ACS group, PR-PCI and PR-PCI group, which were almost no relevant reports. Our results demonstrated that the accurate model could identify novel biomarkers in different types of CAD.

Previous studies have demonstrated a wide range of metabolites associated with CAD [23–36]. Our study has a high correlation with some of them. Lysine acetylation modification is a reversible posttranslational modification that affects enzyme activity, DNA binding force, and protein stability by changing the charge on lysine residues and the structure of proteins. Wang et al. found higher N-acetylthreonine levels were identified to be a biomarker associated with heart failure risk [12]. Li et al. reported lysine acetylation was found closely related to CAD [24]. In our studies, N-Acetyl-lysine was upregulated in both SCAD and ACS patients compared with healthy control. This was consistent with previous research. Serum sterols were a risk factor for CAD [25]. Abnormal metabolic pathways of cholesterol to bile acid could lead to cholesterolemia, which was involved in the occurrence and development of CAD [26]. Bhat et al. found that low excretion of bile acids might promote CAD [27]. When compared with healthy control, 5-Cholesten-*β*, 25(S)-diol, and biliverdin were found upregulated in CAD patients. It suggested that the primary bile acids were decreased in CAD patients. As an ischemia marker, hypoxanthine had been identified in ACS [28]. As an ATP degradation product, upregulation of hypoxanthine was observed in CAD patients in this study. We found that the energy metabolism was different between healthy and CAD subjects. Tryptophan, an ingredient to generate amino acids, was at high levels in CAD patients. A previous study indicated that activated amino acid biosynthesis was an indicator for CAD [29]. As the most important part of the urea cycle, ornithine was obtained from arginine by arginase. Arginine was negatively associated with CAD risk which had been reported [30]. Compared with patients with normal coronary arteries, patients with CAD downregulated lecithin, phosphatidylcholine, pantetheine, and indole as shown in Supplementary Material 1. Low lecithin cholesterol acyltransferase activity had been linked to CAD [31]. Many phosphatidylcholines (PCs) exhibited a negative association with CAD [32]. Our results showed that PC (12 : 0/22 : 2) and PC (24 : 4/12 : 0) were downregulated in CAD patients, which was consistent with other studies [33]. The changed insulin sensitivity and glycemic control were associated with an increased cardiovascular risk in previous reports [34]. The adenosine, inosine, and hypoxanthine, which were released by the oxygen-deprived heart were AMP catabolites. Inosine is a sensitive and early indicator of wall-thickness changes in the ischemic pig hearts [35]. Vitamin E with its major isoforms *α*‐tocopherol (*α*‐T) and *γ*‐tocopherol (*γ*‐T) and reduced glutathione (GSH) are the main antioxidants in the blood. Supplementation with antioxidant micronutrients could be beneficial for CAD [36]. The levels of lecithin were downregulated in CAD patients. Plasma lipids and fatty acids had been linked to CAD. Linoleic acid deficiency had been proposed as a risk factor for cardiovascular disease. Decreased hexadecanoic suggested an elevated level of fatty acids in the metabolism [37].

Different from other studies, we also obtained some new biomarkers in our study. Our results indicated that amino acid metabolism and biosynthesis could also be used as a new marker to distinguish SCAD from ACS. Tyrosine was increased in CAD patients which suggested the role of amino acid disorder in the CAD process. In addition, L-ornithine and ornithine were also found to significantly increase in the ACS group which had not been reported. PE and PC are two major subclasses of glycerophospholipids. PE is a glycerophospholipid in which a phosphoryl ethanolamine moiety occupies a glycerol substitution site. Though some Lyso PC, PC, and Lyso PE were identified to have a negative association with CAD, different classes of PC and PE might be expressed differently. Xu et al. reported most PE species showed no significant differences between AMI and stable angina patients [32]. It seemed that PEs had a strong negative association with CAD. However, in this study, the levels of PEs (P-16 : 0/0 : 0) and PE(P-18 : 0/0 : 0) were both upregulated in healthy versus CAD and SCAD versus ACS. The results suggested that some PEs might contribute to unstable plaque progress. PA (18 : 2/0 : 0) and PA (20 : 4/0 : 0) exhibited different expression levels compared with the CAD group and healthy control in our studies. However, PA (18 : 2/0 : 0), PA (20 : 4/0 : 0), and PA (22 : 4/0 : 0) were increased in ACS patients (versus SCAD), which could be used as a distinction between ACS and SCAD. PA is rarely reported as a biomarker for CAD diagnosis. As primary bile acids were found to be decreased in CAD patients, the higher level of PA might attribute to the cost of cholesterol in the synthesis of bile acids. The increased *α*-d-glucose in ACS patients was blood-related to the energy metabolism disorder compared to patients with SCAD. Interestingly, *α*-tocopherol and 5-oxoproline were upregulated in ACS patients compared with SCAD which suggested the body's autoregulatory function was stronger in ACS. We further investigated the influences of PR-PCI and PR-PCI treatment on patients with ACS. Besides proline, 5-oxoproline, L-proline, primary bile acids, glycine, cholate, the levels of methacrylyl-coA, and adrenosterone of PO-PCI were upregulated compared with PR-PCI patients. PE (22 : 5/0 : 0) and bilirubin were downregulated in PO-PCI patients which alleviated the symptom of CAD. Several new biomarkers were identified from this study with PCI treatment for CAD. These newly found biomarkers enhanced the power for discrimination of different types of CAD.

## 5. Limitation of This Study

The limitations of this study mainly include the following aspects. Firstly, although the UPLC-MS technology has high detection sensitivity [33, 34], there are still many difficulties in the identification and accurate quantification of trace substances [35]. Secondly, in this study, the number of subjects was relatively small, and the required sufficient samples are not obtained. Whether there are metabolic differences between men and women is not clear. Thirdly, the metabolites of HC, SCAD, ACS, and ACS treated by PCI or not were screened out only by LC-MS. These different metabolites still need to be verified through tracking the related upstream and downstream genes or enzymes in subsequent studies.

## 6. Conclusion

This study used UPLC-MS for metabolomics analysis in healthy subjects, SCAD, and ACS with PR-PCI or PO-PCI in positive and negative modes. There were 17 different metabolites between the healthy subjects and SCAD, 15 between SCAD and ACS, and 7 between PR-PCI and PO-PCI groups. The results suggested that CAD was closely related to energy metabolism, lipid metabolism, and glucose metabolism disorders. In summary, the altered metabolites can be used as special metabolic biomarkers for patients with different types of CAD in the early diagnosis.

## Figures and Tables

**Table 1:** The clinical data for the human plasma samples. Values are presented as mean ± SD. SBP: systolic blood pressure; Cr: creatine. Na: sodium; K: potassium; BMI: body mass index.

Clinical indicator	HC	SCAD	ACS	*p* value (HC vs. SCAD)	*p* value (HC vs. ACS)	*p* value (SCAD vs. ACS)
Sex	Male	Male	Male	—	—	—
Age (year)	52.7 ± 8.4	55.1 ± 7.9	56.5 ± 6.7	0.143	0.180	0.395
SBP (mmHg)	136.8 ± 20.9	132.75 ± 23.7	142.4 ± 20.5	0.381	0.254	0.055
Urea (mmol/L)	5.7 ± 1.4	5.6 ± 1.9	6.2 ± 2.5	0.614	0.294	0.164
Cr (*μ*mol/L)	71.7 ± 12.5	70.7 ± 13.5	73.6 ± 18.2	0.686	0.605	0.384
Na (mEq/L)	141.7 ± 2.3	141.8 ± 3.5	140.6 ± 3.2	0.784	0.099	0.099
K (mEq/L)	4.4 ± 0.4	4.4 ± 0.5	4.4 ± 0.4	0.792	0.832	0.973
GLU (mmol/L)	4.9 ± 0.4	4.9 ± 0.5	5.0 ± 0.4	0.963	0.840	0.884
LDL-c (mg/Dl)	80.7 ± 26.6	89.2 ± 14.6	89.9 ± 16.7	0.746	0.852	0.913
Smoking, *n*(%)	21 (50%)	40 (66.6%)	22 (48.9%)	0.288	0.747	0.107
BMI (kg/m^2^)	25.7 ± 1.6	25.5 ± 1.5	25.7 ± 1.6	0.765	0.967	0.737

**Table 2:** Differentiation of metabolites in experimental groups.

Group	Metabolites	m/z	Retention Time(min)	VIP	*p* Value	Fold change
HC vs. CAD	N6Acetyl-L-lysine	171.113	0.660	3.620	<0.001	1.847
	Tyramine	120.080	3.011	2.732	<0.001	1.402
	Biliverdin	583.254	4.898	2.45	<0.001	1.936
	25-Hydroxycholesterol	425.340	6.826	1.954	<0.01	1.287
	Phenol	93.034	2.094	2.177	<0.01	1.273
	Urocanic acid	174.988	2.095	1.428	<0.05	1.175
	L-tryptophane	205.097	3.145	2.588	<0.01	1.798
	L-palmitoylcarnitine	422.326	5.880	1.284	<0.05	1.385
	Hypoxanthine	137.045	1.048	1.768	<0.05	1.809
	PE (P-16 : 0/0 : 0)	436.282	6.632	2.466	<0.001	1.212
	PE (P-18 : 1/0 : 0)	462.299	7.579	3.155	<0.001	1.388
	PA (18 : 2/0 : 0)	433.235	5.552	2.272	<0.05	1.739
	PA (20 : 4/0 : 0)	457.235	5.580	1.852	<0.05	1.768
	PC (12 : 0/22 : 2)	758.569	7.152	1.505	<0.05	0.848
	PC (24 : 4/12 : 0)	804.550	7.619	1.764	<0.05	0.866
	Pantothete	220.118	1.444	1.412	<0.05	0.841
	Indole	257.112	1.416	2.068	<0.01	0.846

SCAD vs. PR-ACS	N-Acetyl-L-lysine	171.112	0.660	2.565	<0.05	1.410
	Glycocholic acid	466.328	7.561	2.077	<0.01	1.259
	Alpha-D-Glucose	180.065	3.577	2.243	<0.05	1.247
	N-Acetyl-L-glutamate	265.980	2.871	1.133	<0.05	1.118
	PC (14 : 1/4 : 0)	536.333	4.660	1.841	<0.05	1.291
	*α*-Tocopherol	431.381	9.694	1.176	<0.05	1.215
	Hypoxanthine	137.045	1.048	2.638	<0.001	2.027
	Ornithine	177.061	0.428	2.022	<0.01	1.309
	PE (P-16 : 0/0 : 0)	436.282	6.632	2.642	<0.05	1.145
	PE (P-18 : 0/0 : 0)	464.314	7.579	2.118	<0.05	1.202
	PA (18 : 2/0 : 0)	433.235	5.552	2.275	<0.01	1.514
	PA (20 : 4/0 : 0)	457.235	5.580	2.083	<0.01	1.561
	PA (22 : 4/0 : 0)	485.266	7.505	2.760	<0.05	1.293
	PC (22 : 5/16 : 1)	828.549	9.560	1.264	<0.05	0.858
	PI (16 : 0/20 : 4)	857.518	8.278	2.147	<0.05	0.738

PR-ACS vs. PO-ACS	L-Proline	116.070	4.212	1.082	<0.01	0.998
	gamma-L-Glutamyl-L-valine	247.128	1.563	2.168	<0.05	1.500
	1-Stearoyl-2-oleoyl-sn-glycerol 3-phosphocholine	786.600	7.686	1.568	<0.01	1.437
	1-Oleoyl-sn-glycerol 3-phosphate	455.259	7.463	1.112	<0.001	1.129
	Glycochenodeoxycholate	430.295	4.037	1.215	<0.05	1.272
	Bilirubin	585.270	4.462	1.812	<0.05	0.784
	PE (22 : 5/0 : 0)	550.289	6.502	2.716	<0.01	0.400

## Data Availability

The data in the used to support the findings of the study are available in Supplementary Information files.

## Abbreviations

ACS: Acute coronary syndrome
BMI: Body mass index
CT: Computed tomography
CAD: Coronary atherosclerotic heart disease
GSH: Glutathione
HC: Healthy controls
IVUS: Intravascular ultrasound
NEG: Negative mode
OPLS-DA: Orthogonal projections to latent structures-discriminate analysis
PCI: Percutaneous coronary intervention
PA: Phosphatidic acid
PE: Phosphatidyl ethanolamines
PC: Phosphatidylcholine
POS: Positive mode
PCA: Principal component analysis
QC: Quality control
SBP: Systolic blood pressure
SCAD: Stable coronary artery disease
3D: Three-dimensional
2D: Two-dimensional
UPLC-MS: Ultra-performance liquid chromatography-mass spectrometry
VIP: Variable importance projection.

## Supplementary Materials

Supplementary Materials Supplementary Material 1: metabolic pathways analysis based on the KEGG pathway database in all experimental groups. Figure S1: Figure S1(a)-S1D. Score plot of OPLS-DA model obtained from experimental groups (NEG). black: HC, red: SA, blue: PR-ACS, yellow: PO-ACS. Figure S1(e)- S1H, the validated model of OPLS-DA. 200 times were performed, and the resulting R2 and Q2 values were plotted. (Green triangle): R2; (blue square): Q2. The green line represents the regression line for R2 and the blue line for Q2. Figure S2: loading plot of OPLS-DA model obtained from experimental groups (NEG). A: HC versus SCAD, B: HC versus PR-ACS, C: SCAD versus PR-ACS, D: PR-ACS versus PO-ACS. Figure S3: pathway analysis of experiment group. The larger the circle meant the greater the influence of topology analysis; the redder the color meant the smaller the *p* value, and vice versa. A: HC versus SCAD, B: HC versus PR-ACS, C: SCAD versus PR-ACS, D: PR-ACS versus PO-ACS (NEG).
