# Supplementary material for: The Metabolomic Characterization of Different Types of Coronary Atherosclerotic Heart Disease in Male
Source: Cardiol Res Pract. 2022 Jul 12;2022:6491129. doi: 10.1155/2022/6491129 (PMC9296306; doi:10.1155/2022/6491129)
Supplement: Supplementary Materials — Supplementary Material 1: metabolic pathways analysis based on the KEGG pathway database in all experimental groups. Figure S1: Figure S1(a)-S1D. Score plot of OPLS-DA model obtained from experimental groups (NEG). black: HC, red: SA, blue: PR-ACS, yellow: PO-ACS. Figure S1(e)- S1H, the validated model of OPLS-DA. 200 times were performed, and the resulting R2 and Q2 values were plotted. (Green triangle): R2; (blue square): Q2. The green line represents the regression line for R2 and the blue line for Q2. Figure S2: loading plot of OPLS-DA model obtained from experimental groups (NEG). A: HC versus SCAD, B: HC versus PR-ACS, C: SCAD versus PR-ACS, D: PR-ACS versus PO-ACS. Figure S3: pathway analysis of experiment group. The larger the circle meant the greater the influence of topology analysis; the redder the color meant the smaller the p value, and vice versa. A: HC versus SCAD, B: HC versus PR-ACS, C: SCAD versus PR-ACS, D: PR-ACS versus PO-ACS (NEG). [file 6491129.f1.zip › 6491129.f1/Supplementary Material 1.pdf]

HC vs. CAD

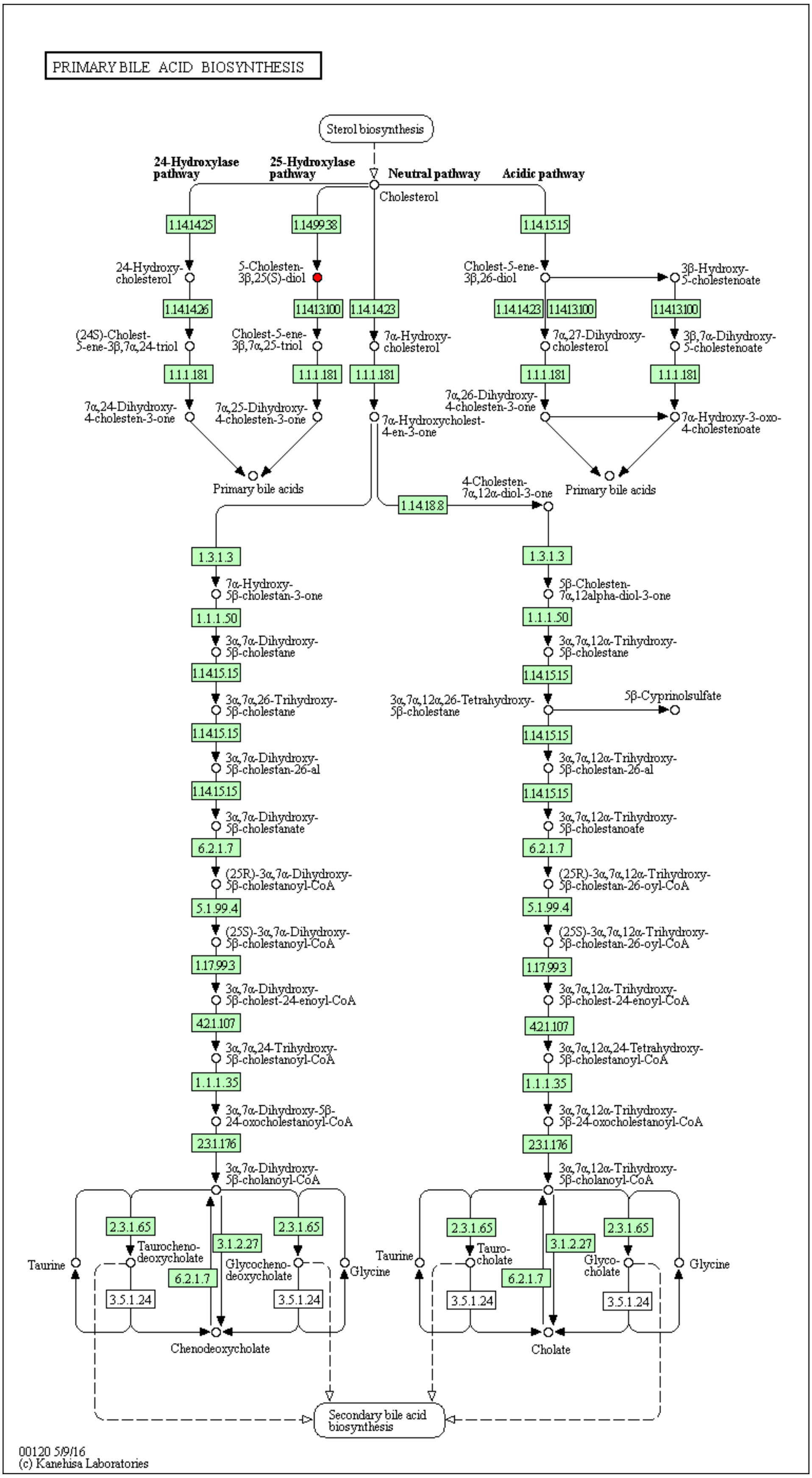
PRIMARY BILE ACID BIOSYNTHESIS
Sterol biosynthesis
24-Hydroxylase pathway
25-Hydroxylase pathway
Neutral pathway
Acidic pathway
Cholesterol
1.14.14.25
1.14.99.38
1.14.15.15
24-Hydroxy-cholesterol
5-Cholesten-3β,25(S)-diol
Cholest-5-ene-3β,26-diol
3β-Hydroxy-5-cholestenoate
1.14.14.26
1.14.13.100
1.14.14.23
(24S)-Cholest-5-ene-3β,7α,24-triol
Cholest-5-ene-3β,7α,25-triol
7α-Hydroxy-cholesterol
7α,27-Dihydroxy-cholesterol
3β,7α-Dihydroxy-5-cholestenoate
1.1.1.181
7α,24-Dihydroxy-4-cholesten-3-one
7α,25-Dihydroxy-4-cholesten-3-one
7α-Hydroxycholest-4-en-3-one
7α,26-Dihydroxy-4-cholesten-3-one
7α-Hydroxy-3-oxo-4-cholestenoate
Primary bile acids
1.14.18.8
4-Cholesten-7α,12α-diol-3-one
1.3.1.3
7α-Hydroxy-5β-cholestan-3-one
5β-Cholesten-7α,12alpha-diol-3-one
1.1.1.50
3α,7α-Dihydroxy-5β-cholestane
3α,7α,12α-Trihydroxy-5β-cholestane
3α,7α,26-Trihydroxy-5β-cholestane
3α,7α,12α,26-Tetrahydroxy-5β-cholestane
5β-Cyprinolsulfate
3α,7α-Dihydroxy-5β-cholestan-26-al
3α,7α,12α-Trihydroxy-5β-cholestan-26-al
3α,7α-Dihydroxy-5β-cholestanate
3α,7α,12α-Trihydroxy-5β-cholestanoate
6.2.1.7
(25R)-3α,7α-Dihydroxy-5β-cholestanoyl-CoA
(25R)-3α,7α,12α-Trihydroxy-5β-cholestan-26-oyl-CoA
5.1.99.4
(25S)-3α,7α-Dihydroxy-5β-cholestanoyl-CoA
(25S)-3α,7α,12α-Trihydroxy-5β-cholestan-26-oyl-CoA
1.17.99.3
3α,7α-Dihydroxy-5β-cholest-24-enoyl-CoA
3α,7α,12α-Trihydroxy-5β-cholest-24-enoyl-CoA
4.2.1.107
3α,7α,24-Trihydroxy-5β-cholestanoyl-CoA
3α,7α,12α,24-Tetrahydroxy-5β-cholestanoyl-CoA
1.1.1.35
3α,7α-Dihydroxy-5β-24-oxocholestanoyl-CoA
3α,7α,12α-Trihydroxy-5β-24-oxocholestanoyl-CoA
2.3.1.176
3α,7α-Dihydroxy-5β-cholanoyl-CoA
3α,7α,12α-Trihydroxy-5β-cholanoyl-CoA
2.3.1.65
3.1.2.27
Taurine
Taurocheno-deoxycholate
Glycocheno-deoxycholate
Glycine
Tauro-cholate
Glyco-cholate
3.5.1.24
Chenodeoxycholate
Cholate
Secondary bile acid biosynthesis
00120 5/9/16
(c) Kanehisa Laboratories

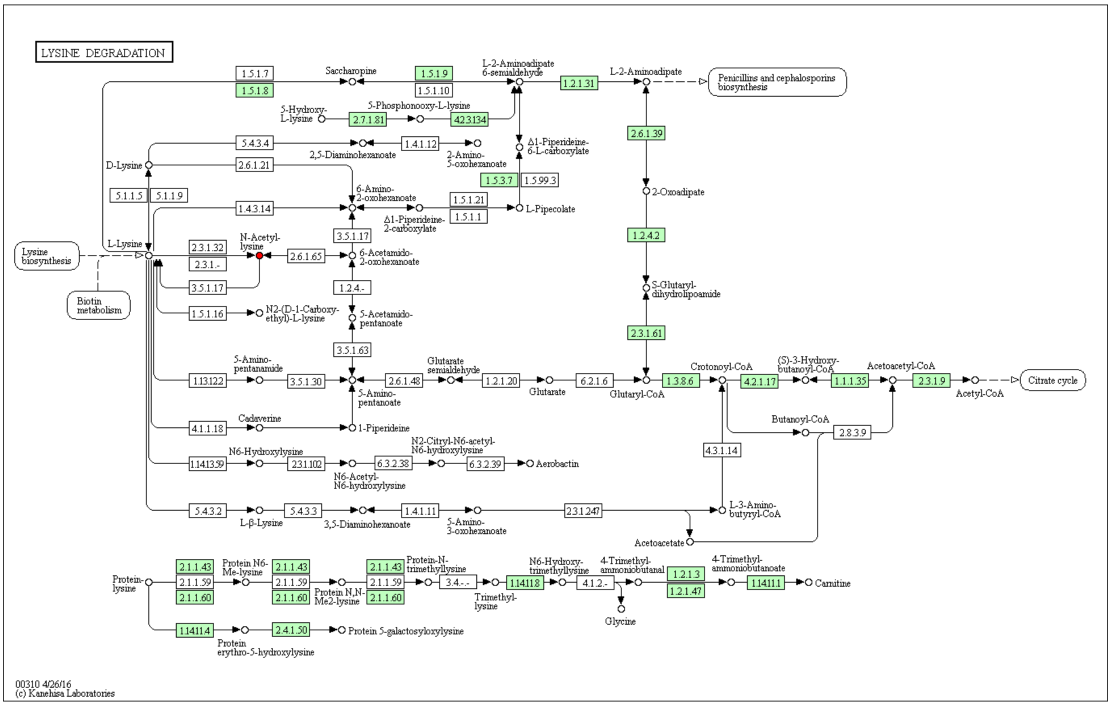
LYSINE DEGRADATION
Saccharopine
L-2-Aminoadipate 6-semialdehyde
L-2-Aminoadipate
Penicillins and cephalosporins biosynthesis
5-Hydroxy-L-lysine
5-Phosphonooxy-L-lysine
2,5-Diaminohexanoate
2-Amino-5-oxohexanoate
Δ1-Piperideine-6-L-carboxylate
D-Lysine
6-Amino-2-oxohexanoate
Δ1-Piperideine-2-carboxylate
L-Pipecolate
2-Oxoadipate
L-Lysine
Lysine biosynthesis
N-Acetyl-lysine
6-Acetamido-2-oxohexanoate
Biotin metabolism
N2-(D-1-Carboxyethyl)-L-lysine
5-Acetamido-pentanoate
S-Glutaryl-dihydrolipoamide
5-Amino-pentanamide
5-Amino-pentanoate
Glutarate semialdehyde
Glutarate
Glutaryl-CoA
Crotonoyl-CoA
(S)-3-Hydroxy-butanoyl-CoA
Acetoacetyl-CoA
Acetyl-CoA
Citrate cycle
Cadaverine
1-Piperideine
Butanoyl-CoA
N6-Hydroxylysine
N2-Citryl-N6-acetyl-N6-hydroxylysine
N6-Acetyl-N6-hydroxylysine
Aerobactin
L-β-Lysine
3,5-Diaminohexanoate
5-Amino-3-oxohexanoate
L-3-Amino-butyryl-CoA
Acetoacetate
Protein-lysine
Protein N6-Me-lysine
Protein N,N-Me2-lysine
Protein-N-trimethyllysine
N6-Hydroxy-trimethyllysine
Trimethyl-lysine
4-Trimethyl-ammoniobutanal
4-Trimethyl-ammoniobutanoate
Carnitine
Glycine
Protein erythro-5-hydroxylysine
Protein 5-galactosyloxylysine
00310 4/26/16
(c) Kanehisa Laboratories

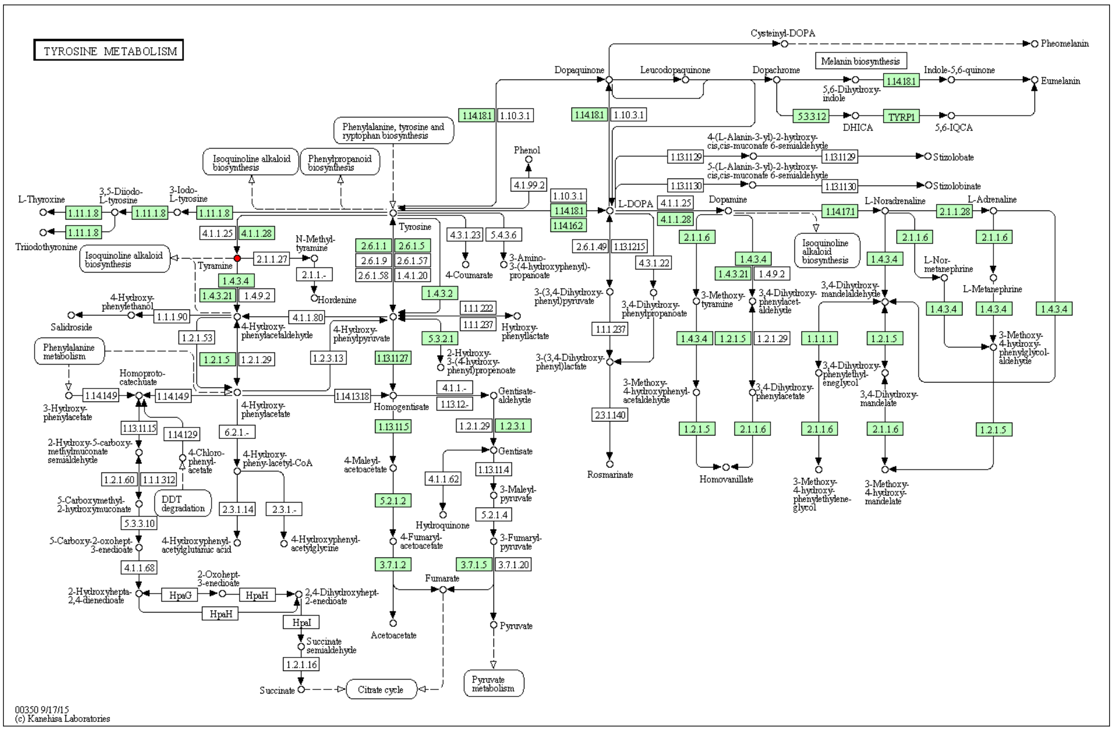
TYROSINE METABOLISM
Cysteinyl-DOPA
Pheomelanin
Dopaquinone
Leucodopaquinone
Dopachrome
Melanin biosynthesis
Indole-5,6-quinone
Eumelanin
5,6-Dihydroxy-indole
DHICA
5,6-IQCA
Phenylalanine, tyrosine and tryptophan biosynthesis
Isoquinoline alkaloid biosynthesis
Phenylpropanoid biosynthesis
Phenol
4-(L-Alanin-3-yl)-2-hydroxy-cis,cis-muconate 6-semialdehyde
5-(L-Alanin-3-yl)-2-hydroxy-cis,cis-muconate 6-semialdehyde
Stizolobate
Stizolobinate
L-Thyroxine
3,5-Diiodo-L-tyrosine
3-Iodo-L-tyrosine
Triiodothyronine
Tyrosine
L-DOPA
Dopamine
L-Noradrenaline
L-Adrenaline
N-Methyl-tyramine
Tyramine
Hordenine
4-Coumarate
3-Amino-3-(4-hydroxyphenyl)-propanoate
3-(3,4-Dihydroxy-phenyl)pyruvate
3,4-Dihydroxy-phenylpropanoate
3-(3,4-Dihydroxy-phenyl)lactate
Rosmarinate
3-Methoxy-tyramine
3,4-Dihydroxy-phenylacet-aldehyde
3,4-Dihydroxy-phenylacetate
3-Methoxy-4-hydroxyphenyl-acetaldehyde
Homovanillate
3,4-Dihydroxy-mandelaldehyde
L-Nor-metanephrine
L-Metanephrine
3-Methoxy-4-hydroxy-phenylglycol-aldehyde
3,4-Dihydroxy-phenylethyl-eneglycol
3,4-Dihydroxy-mandelate
3-Methoxy-4-hydroxy-phenylethylene-glycol
3-Methoxy-4-hydroxy-mandelate
4-Hydroxy-phenylethanol
Salidroside
4-Hydroxy-phenylacetaldehyde
4-Hydroxy-phenylpyruvate
Hydroxy-phenyllactate
2-Hydroxy-3-(4-hydroxy-phenyl)propenoate
Phenylalanine metabolism
Homoproto-catechuate
3-Hydroxy-phenylacetate
4-Hydroxy-phenylacetate
Homogentisate
Gentisate-aldehyde
Gentisate
2-Hydroxy-5-carboxy-methylmuconate semialdehyde
4-Chloro-phenyl-acetate
4-Hydroxy-pheny-lacetyl-CoA
4-Maleyl-acetoacetate
3-Maleyl-pyruvate
Hydroquinone
5-Carboxymethyl-2-hydroxymuconate
DDT degradation
5-Carboxy-2-oxohept-3-enedioate
4-Hydroxyphenyl-acetylglutamic acid
4-Hydroxyphenyl-acetylglycine
4-Fumaryl-acetoacetate
3-Fumaryl-pyruvate
Fumarate
2-Hydroxyhepta-2,4-dienedioate
2-Oxohept-3-enedioate
2,4-Dihydroxyhept-2-enedioate
HpaG
HpaH
HpaI
Acetoacetate
Pyruvate
Succinate semialdehyde
Succinate
Citrate cycle
Pyruvate metabolism
00350 9/17/15
(c) Kanehisa Laboratories

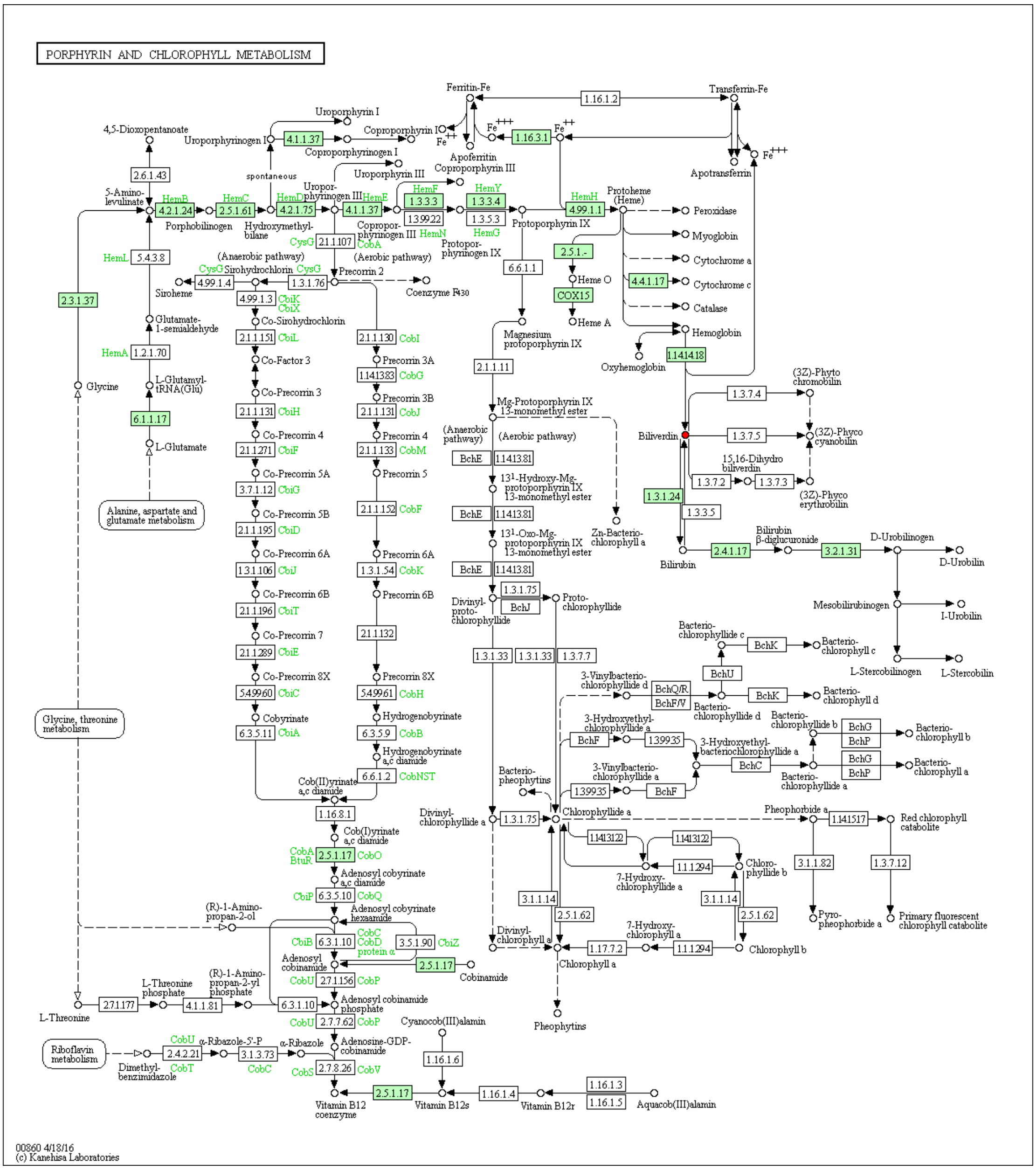
PORPHYRIN AND CHLOROPHYLL METABOLISM
Ferritin-Fe
1.16.1.2
Transferrin-Fe
Uroporphyrin I
4,5-Dioxopentanoate
Uroporphyrinogen I
4.1.1.37
Coproporphyrin I
Coproporphyrinogen I
1.16.3.1
Apoferritin
Coproporphyrin III
Apotransferrin
2.6.1.43
spontaneous
Uroporphyrin III
5-Amino-levulinate
HemB
4.2.1.24
HemC
2.5.1.61
HemD
4.2.1.75
Uropor-phyrinogen III
HemE
4.1.1.37
HemF
1.3.3.3
1.3.99.22
HemY
1.3.3.4
1.3.5.3
HemH
4.99.1.1
Protoheme (Heme)
Peroxidase
Myoglobin
Porphobilinogen
Hydroxymethyl-bilane
Copropor-phyrinogen III
HemN
HemG
Protopor-phyrinogen IX
Protoporphyrin IX
CysG
2.1.1.107
CobA
HemL
5.4.3.8
(Anaerobic pathway)
(Aerobic pathway)
2.5.1.-
Cytochrome a
4.4.1.17
Cytochrome c
Catalase
Hemoglobin
Heme O
COX15
Heme A
CysG Sirohydrochlorin CysG
Precorrin 2
4.99.1.4
1.3.1.76
Coenzyme F430
Siroheme
4.99.1.3
CbiK
CbiX
6.6.1.1
2.3.1.37
Glutamate-1-semialdehyde
Co-Sirohydrochlorin
2.1.1.151
CbiL
2.1.1.130
CbiI
Magnesium protoporphyrin IX
HemA
1.2.1.70
Co-Factor 3
Precorrin 3A
1.14.13.83
CobG
2.1.1.11
Oxyhemoglobin
1.14.14.18
Glycine
L-Glutamyl-tRNA(Glu)
Co-Precorrin 3
Precorrin 3B
(3Z)-Phyto chromobilin
1.3.7.4
2.1.1.131
CbiH
2.1.1.131
CobJ
Mg-Protoporphyrin IX 13-monomethyl ester
6.1.1.17
Co-Precorrin 4
Precorrin 4
(Anaerobic pathway)
(Aerobic pathway)
Biliverdin
1.3.7.5
(3Z)-Phyco cyanobilin
L-Glutamate
2.1.1.271
CbiF
2.1.1.133
CobM
BchE
1.14.13.81
15,16-Dihydro biliverdin
Co-Precorrin 5A
Precorrin 5
1.3.7.2
1.3.7.3
3.7.1.12
CbiG
13[1]-Hydroxy-Mg-protoporphyrin IX 13-monomethyl ester
1.3.1.24
(3Z)-Phyco erythrobilin
Alanine, aspartate and glutamate metabolism
Co-Precorrin 5B
2.1.1.152
CobF
BchE
1.14.13.81
1.3.3.5
2.1.1.195
CbiD
13[1]-Oxo-Mg-protoporphyrin IX 13-monomethyl ester
Zn-Bacterio-chlorophyll a
Bilirubin β-diglucuronide
D-Urobilinogen
Co-Precorrin 6A
Precorrin 6A
Bilirubin
2.4.1.17
3.2.1.31
D-Urobilin
1.3.1.106
CbiJ
1.3.1.54
CobK
BchE
1.14.13.81
1.3.1.75
Co-Precorrin 6B
Precorrin 6B
Divinyl-proto-chlorophyllide
BchJ
Proto-chlorophyllide
Mesobilirubinogen
I-Urobilin
2.1.1.196
CbiT
Bacterio-chlorophyllide c
BchK
Bacterio-chlorophyll c
Co-Precorrin 7
2.1.1.132
1.3.1.33
1.3.1.33
1.3.7.7
BchU
L-Stercobilinogen
L-Stercobilin
2.1.1.289
CbiE
3-Vinylbacterio-chlorophyllide d
BchQ/R
BchF/V
BchK
Bacterio-chlorophyll d
Co-Precorrin 8X
Precorrin 8X
Bacterio-chlorophyllide d
5.4.99.60
CbiC
5.4.99.61
CobH
3-Hydroxyethyl-chlorophyllide a
Bacterio-chlorophyllide b
BchG
BchP
Bacterio-chlorophyll b
Glycine, threonine metabolism
Cobyrinate
Hydrogenobyrinate
BchF
1.3.99.35
3-Hydroxyethyl-bacteriochlorophyllide a
6.3.5.11
CbiA
6.3.5.9
CobB
BchC
BchG
BchP
Bacterio-chlorophyll a
Hydrogenobyrinate a,c diamide
Bacterio-pheophytins
3-Vinylbacterio-chlorophyllide a
Bacterio-chlorophyllide a
Cob(II)yrinate a,c diamide
6.6.1.2
CobNST
1.3.99.35
BchF
1.16.8.1
Divinyl-chlorophyllide a
1.3.1.75
Chlorophyllide a
Pheophorbide a
1.14.15.17
Red chlorophyll catabolite
Cob(I)yrinate a,c diamide
1.14.13.122
1.14.13.122
CobA
BtuR
2.5.1.17
CobO
1.1.1.294
Chloro-phyllide b
3.1.1.82
1.3.7.12
Adenosyl cobyrinate a,c diamide
7-Hydroxy-chlorophyllide a
CbiP
6.3.5.10
CobQ
3.1.1.14
3.1.1.14
(R)-1-Amino-propan-2-ol
Adenosyl cobyrinate hexaamide
2.5.1.62
2.5.1.62
Pyro-pheophorbide a
Primary fluorescent chlorophyll catabolite
CobC
CbiB
6.3.1.10
CobD
3.5.1.90
CbiZ
7-Hydroxy-chlorophyll a
Divinyl-chlorophyll a
protein α
1.17.7.2
1.1.1.294
Chlorophyll b
Adenosyl cobinamide
2.5.1.17
Cobinamide
Chlorophyll a
L-Threonine phosphate
(R)-1-Amino-propan-2-yl phosphate
CobU
2.7.1.156
CobP
2.7.1.177
4.1.1.81
6.3.1.10
Adenosyl cobinamide phosphate
L-Threonine
CobU
2.7.7.62
CobP
Cyanocob(III)alamin
Pheophytins
Riboflavin metabolism
CobU
α-Ribazole-5'-P
α-Ribazole
Adenosine-GDP-cobinamide
2.4.2.21
3.1.3.73
1.16.1.6
Dimethyl-benzimidazole
CobT
CobC
CobS
2.7.8.26
CobV
1.16.1.3
2.5.1.17
1.16.1.4
1.16.1.5
Vitamin B12 coenzyme
Vitamin B12s
Vitamin B12r
Aquacob(III)alamin
00860 4/18/16
(c) Kanehisa Laboratories

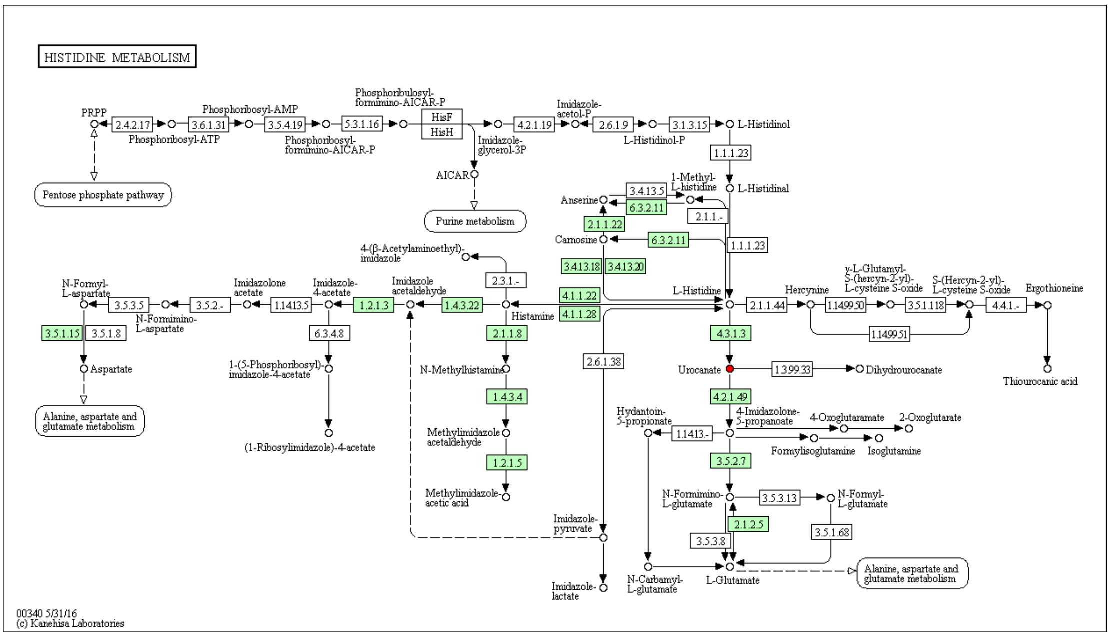
HISTIDINE METABOLISM
PRPP
2.4.2.17
Phosphoribosyl-ATP
3.6.1.31
Phosphoribosyl-AMP
3.5.4.19
Phosphoribosyl-formimino-AICAR-P
5.3.1.16
Phosphoribulosyl-formimino-AICAR-P
HisF
HisH
AICAR
Purine metabolism
Imidazole-glycerol-3P
4.2.1.19
Imidazole-acetol-P
2.6.1.9
L-Histidinol-P
3.1.3.15
L-Histidinol
1.1.1.23
L-Histidinal
Pentose phosphate pathway
1-Methyl-L-histidine
3.4.13.5
6.3.2.11
Anserine
2.1.1.22
2.1.1.-
Carnosine
6.3.2.11
3.4.13.18
3.4.13.20
L-Histidine
4-(β-Acetylaminoethyl)-imidazole
2.3.1.-
N-Formyl-L-aspartate
3.5.3.5
N-Formimino-L-aspartate
3.5.2.-
Imidazolone acetate
1.14.13.5
Imidazole-4-acetate
1.2.1.3
Imidazole acetaldehyde
1.4.3.22
Histamine
4.1.1.22
4.1.1.28
3.5.1.15
3.5.1.8
Aspartate
Alanine, aspartate and glutamate metabolism
6.3.4.8
1-(5-Phosphoribosyl)-imidazole-4-acetate
(1-Ribosylimidazole)-4-acetate
2.1.1.8
N-Methylhistamine
1.4.3.4
Methylimidazole acetaldehyde
1.2.1.5
Methylimidazole-acetic acid
2.6.1.38
Imidazole-pyruvate
Imidazole-lactate
Hercynine
2.1.1.44
1.14.99.50
γ-L-Glutamyl-S-(hercyn-2-yl)-L-cysteine S-oxide
3.5.1.118
S-(Hercyn-2-yl)-L-cysteine S-oxide
4.4.1.-
Ergothioneine
1.14.99.51
Thiourocanic acid
4.3.1.3
Urocanate
1.3.99.33
Dihydrourocanate
4.2.1.49
4-Imidazolone-5-propanoate
4-Oxoglutaramate
2-Oxoglutarate
Formylisoglutamine
Isoglutamine
Hydantoin-5-propionate
1.14.13.-
3.5.2.7
N-Formimino-L-glutamate
3.5.3.13
N-Formyl-L-glutamate
2.1.2.5
3.5.3.8
3.5.1.68
N-Carbamyl-L-glutamate
L-Glutamate
Alanine, aspartate and glutamate metabolism
00340 5/31/16
(c) Kanehisa Laboratories

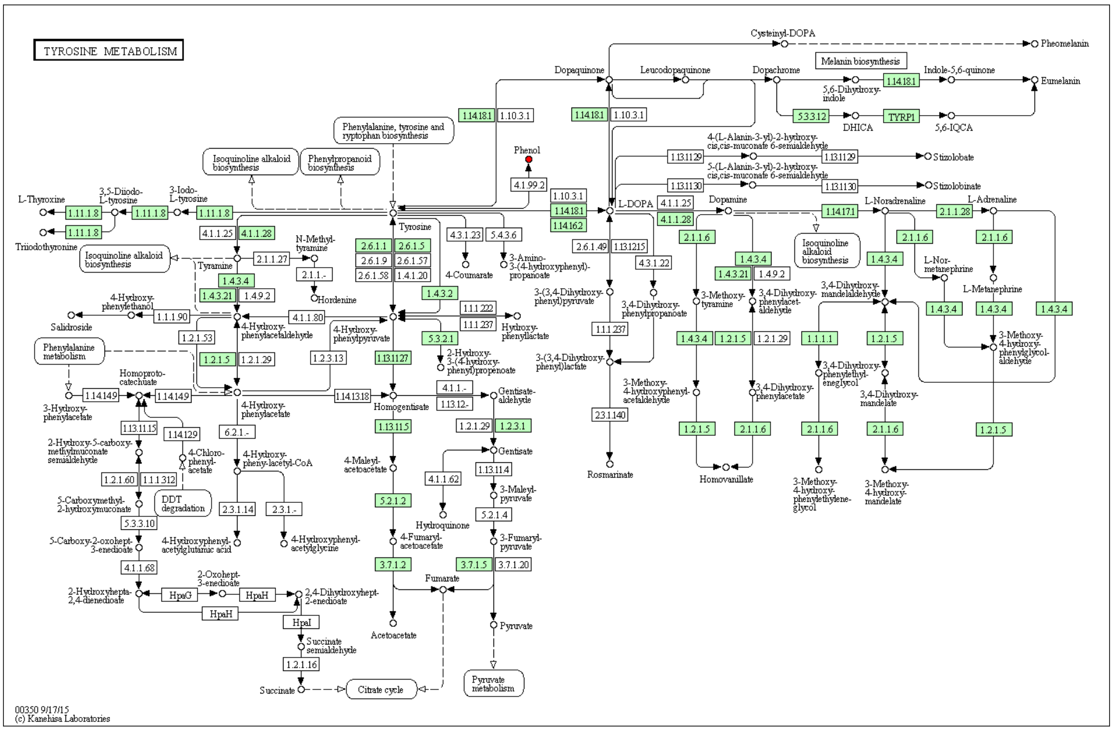
TYROSINE METABOLISM
Cysteinyl-DOPA
Pheomelanin
Dopaquinone
Leucodopaquinone
Dopachrome
Melanin biosynthesis
5,6-Dihydroxy-indole
1.14.18.1
Indole-5,6-quinone
Eumelanin
5.3.3.12
DHICA
TYRP1
5,6-IQCA
1.14.18.1
1.10.3.1
Phenylalanine, tyrosine and tryptophan biosynthesis
Isoquinoline alkaloid biosynthesis
Phenylpropanoid biosynthesis
Phenol
4.1.99.2
4-(L-Alanin-3-yl)-2-hydroxy-cis,cis-muconate 6-semialdehyde
1.13.11.29
Stizolobate
5-(L-Alanin-3-yl)-2-hydroxy-cis,cis-muconate 6-semialdehyde
1.13.11.30
Stizolobinate
L-Thyroxine
1.11.1.8
3,5-Diiodo-L-tyrosine
3-Iodo-L-tyrosine
Triiodothyronine
Tyrosine
L-DOPA
1.14.16.2
4.1.1.25
4.1.1.28
Dopamine
1.14.17.1
L-Noradrenaline
2.1.1.28
L-Adrenaline
N-Methyl-tyramine
2.1.1.27
Tyramine
Hordenine
1.4.3.4
1.4.3.21
1.4.9.2
4-Hydroxy-phenylethanol
1.1.1.90
Salidroside
4.1.1.80
4-Hydroxy-phenylacetaldehyde
1.2.1.53
1.2.1.5
1.2.1.29
Phenylalanine metabolism
2.6.1.1
2.6.1.5
2.6.1.9
2.6.1.57
2.6.1.58
1.4.1.20
4.3.1.23
5.4.3.6
4-Coumarate
3-Amino-3-(4-hydroxyphenyl)-propanoate
4-Hydroxy-phenylpyruvate
1.4.3.2
1.1.1.222
1.1.1.237
Hydroxy-phenyllactate
5.3.2.1
2-Hydroxy-3-(4-hydroxy-phenyl)propenoate
1.13.11.27
1.23.13
Homoproto-catechuate
1.14.14.9
3-Hydroxy-phenylacetate
1.13.11.15
1.14.12.9
4-Chloro-phenyl-acetate
2-Hydroxy-5-carboxy-methylmuconate semialdehyde
1.2.1.60
1.1.1.312
DDT degradation
5-Carboxymethyl-2-hydroxymuconate
5.3.3.10
5-Carboxy-2-oxohept-3-enedioate
4.1.1.68
2-Hydroxyhepta-2,4-dienedioate
HpaG
2-Oxohept-3-enedioate
HpaH
2,4-Dihydroxyhept-2-enedioate
HpaH
HpaI
Succinate semialdehyde
1.2.1.16
Succinate
Citrate cycle
4-Hydroxy-phenylacetate
6.2.1.-
4-Hydroxy-phenylacetyl-CoA
2.3.1.14
2.3.1.-
4-Hydroxyphenyl-acetylglutamic acid
4-Hydroxyphenyl-acetylglycine
1.14.13.18
Homogentisate
4.1.1.-
1.13.12.-
Gentisate-aldehyde
1.2.1.29
1.2.3.1
Gentisate
1.13.11.5
4-Maleyl-acetoacetate
5.2.1.2
4-Fumaryl-acetoacetate
3.7.1.2
Acetoacetate
Fumarate
4.1.1.62
Hydroquinone
1.13.11.4
3-Maleyl-pyruvate
5.2.1.4
3-Fumaryl-pyruvate
3.7.1.5
3.7.1.20
Pyruvate
Pyruvate metabolism
2.6.1.49
1.13.12.15
4.3.1.22
3-(3,4-Dihydroxy-phenyl)pyruvate
3,4-Dihydroxy-phenylpropanoate
1.1.1.237
3-(3,4-Dihydroxy-phenyl)lactate
2.3.1.140
Rosmarinate
2.1.1.6
3-Methoxy-tyramine
3,4-Dihydroxy-phenylacet-aldehyde
3-Methoxy-4-hydroxyphenyl-acetaldehyde
3,4-Dihydroxy-phenylacetate
Homovanillate
Isoquinoline alkaloid biosynthesis
3,4-Dihydroxy-mandelaldehyde
L-Nor-metanephrine
L-Metanephrine
1.1.1.1
3,4-Dihydroxy-phenylethyl-eneglycol
3,4-Dihydroxy-mandelate
3-Methoxy-4-hydroxy-phenylglycol-aldehyde
3-Methoxy-4-hydroxy-phenylethylene-glycol
3-Methoxy-4-hydroxy-mandelate
00350 9/17/15
(c) Kanehisa Laboratories

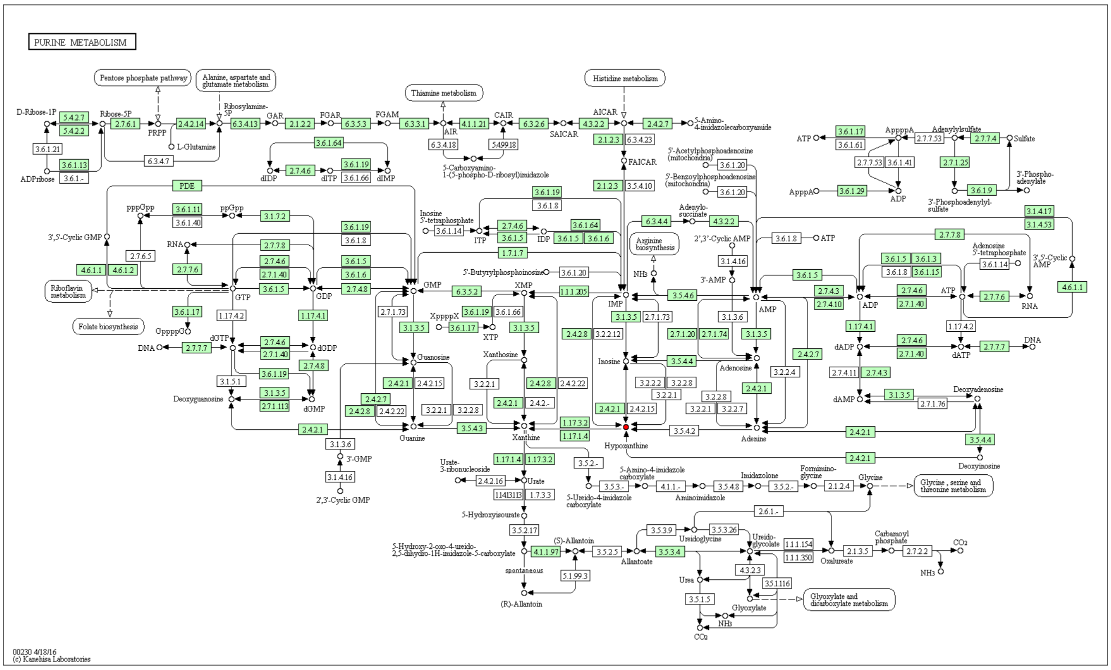
PURINE METABOLISM
Pentose phosphate pathway
Alanine, aspartate and glutamate metabolism
Thiamine metabolism
Histidine metabolism
D-Ribose-1P
Ribose-5P
PRPP
Ribosylamine-5P
L-Glutamine
GAR
FGAR
FGAM
AIR
CAIR
SAICAR
AICAR
FAICAR
5-Amino-4-imidazolecarboxyamide
5-Carboxyamino-1-(5-phospho-D-ribosyl)imidazole
ADPribose
dIDP
dITP
dIMP
PDE
ppGpp
pppGpp
3',5'-Cyclic GMP
RNA
Riboflavin metabolism
Folate biosynthesis
GTP
GDP
GMP
dGTP
dGDP
dGMP
DNA
GppppG
Deoxyguanosine
Guanosine
Guanine
3'-GMP
2',3'-Cyclic GMP
XpppX
XTP
XMP
Xanthosine
Xanthine
Inosine 5'-tetraphosphate
ITP
IDP
IMP
Inosine
Hypoxanthine
5'-Butyrylphosphoinosine
Arginine biosynthesis
NH3
Adenylo-succinate
5'-Acetylphosphoadenosine (mitochondria)
5'-Benzoylphosphoadenosine (mitochondria)
2',3'-Cyclic AMP
3'-AMP
AMP
ADP
ATP
Adenosine
Adenine
ApppA
Adenylylsulfate
Sulfate
3'-Phospho-adenylate
3'-Phosphoadenylyl-sulfate
AppppA
Adenosine 5'-tetraphosphate
3',5'-Cyclic AMP
RNA
dADP
dATP
dAMP
Deoxyadenosine
Deoxyinosine
Urate
Urate-3-ribonucleoside
5-Hydroxyisourate
5-Hydroxy-2-oxo-4-ureido-2,5-dihydro-1H-imidazole-5-carboxylate
(S)-Allantoin
(R)-Allantoin
spontaneous
Allantoate
5-Amino-4-imidazole carboxylate
5-Ureido-4-imidazole carboxylate
Aminoimidazole
Imidazolone
Formimino-glycine
Glycine
Glycine, serine and threonine metabolism
Ureidoglycine
Ureido-glycolate
Oxalureate
Carbamoyl phosphate
CO2
NH3
Urea
Glyoxylate
Glyoxylate and dicarboxylate metabolism
00230 4/18/16
(c) Kanehisa Laboratories

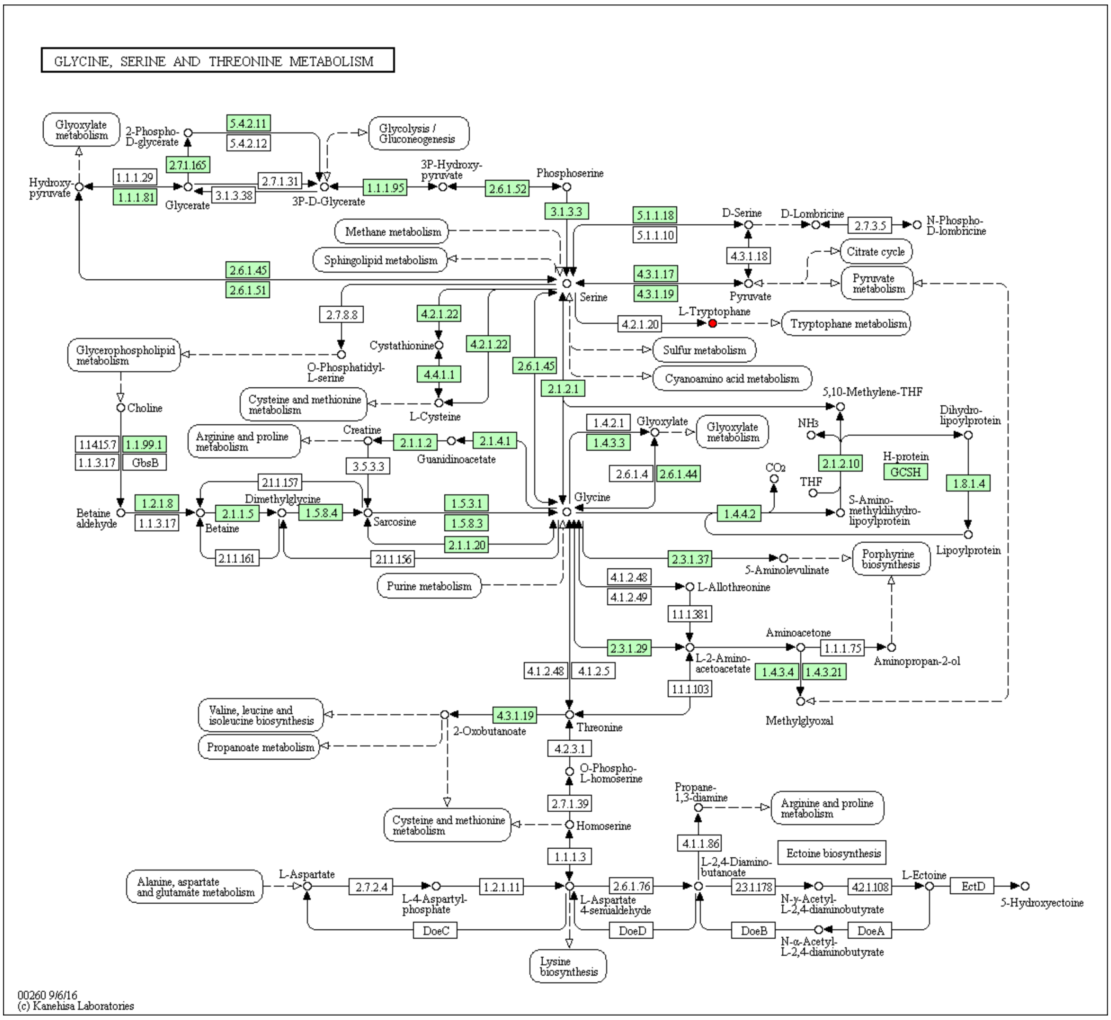
GLYCINE, SERINE AND THREONINE METABOLISM
Glyoxylate metabolism
2-Phospho-D-glycerate
5.4.2.11
5.4.2.12
Glycolysis / Gluconeogenesis
2.7.1.165
Hydroxy-pyruvate
1.1.1.29
1.1.1.81
Glycerate
2.7.1.31
1.1.3.38
3P-D-Glycerate
1.1.1.95
3P-Hydroxy-pyruvate
2.6.1.52
Phosphoserine
3.1.3.3
Methane metabolism
Sphingolipid metabolism
5.1.1.18
5.1.1.10
D-Serine
D-Lombricine
2.7.3.5
N-Phospho-D-lombricine
4.3.1.18
Citrate cycle
Pyruvate metabolism
2.6.1.45
2.6.1.51
4.3.1.17
4.3.1.19
Serine
Pyruvate
2.7.8.8
4.2.1.22
4.2.1.20
L-Tryptophane
Tryptophane metabolism
Glycerophospholipid metabolism
O-Phosphatidyl-L-serine
Cystathionine
4.2.1.22
Sulfur metabolism
Cyanoamino acid metabolism
4.4.1.1
2.6.1.45
2.1.2.1
Choline
Cysteine and methionine metabolism
L-Cysteine
5,10-Methylene-THF
Dihydro-lipoylprotein
1.4.2.1
Glyoxylate
Glyoxylate metabolism
NH3
Arginine and proline metabolism
Creatine
2.1.1.2
2.1.4.1
1.4.3.3
1.14.15.7
1.1.99.1
Guanidinoacetate
2.1.2.10
H-protein
1.1.3.17
GbsB
3.5.3.3
GCSH
2.6.1.4
2.6.1.44
CO2
THF
1.8.1.4
2.1.1.157
1.2.1.8
Dimethylglycine
1.5.3.1
Glycine
S-Amino-methyldihydro-lipoylprotein
Betaine aldehyde
2.1.1.5
1.5.8.4
Sarcosine
1.4.4.2
1.1.3.17
Betaine
1.5.8.3
2.1.1.20
Lipoylprotein
2.1.1.161
2.1.1.156
2.3.1.37
Porphyrine biosynthesis
5-Aminolevulinate
Purine metabolism
4.1.2.48
L-Allothreonine
4.1.2.49
1.1.1.381
Aminoacetone
2.3.1.29
L-2-Amino-acetoacetate
1.1.1.75
Aminopropan-2-ol
4.1.2.48
4.1.2.5
1.4.3.4
1.4.3.21
1.1.1.103
Valine, leucine and isoleucine biosynthesis
4.3.1.19
Threonine
Methylglyoxal
2-Oxobutanoate
Propanoate metabolism
4.2.3.1
O-Phospho-L-homoserine
Propane-1,3-diamine
Arginine and proline metabolism
2.7.1.39
Cysteine and methionine metabolism
Homoserine
4.1.1.86
Ectoine biosynthesis
1.1.1.3
L-2,4-Diamino-butanoate
Alanine, aspartate and glutamate metabolism
L-Aspartate
2.7.2.4
L-4-Aspartyl-phosphate
1.2.1.11
2.6.1.76
L-Aspartate 4-semialdehyde
2.3.1.178
N-γ-Acetyl-L-2,4-diaminobutyrate
4.2.1.108
L-Ectoine
EctD
5-Hydroxyectoine
DoeC
DoeD
DoeB
N-α-Acetyl-L-2,4-diaminobutyrate
DoeA
Lysine biosynthesis
00260 9/6/16
(c) Kanehisa Laboratories

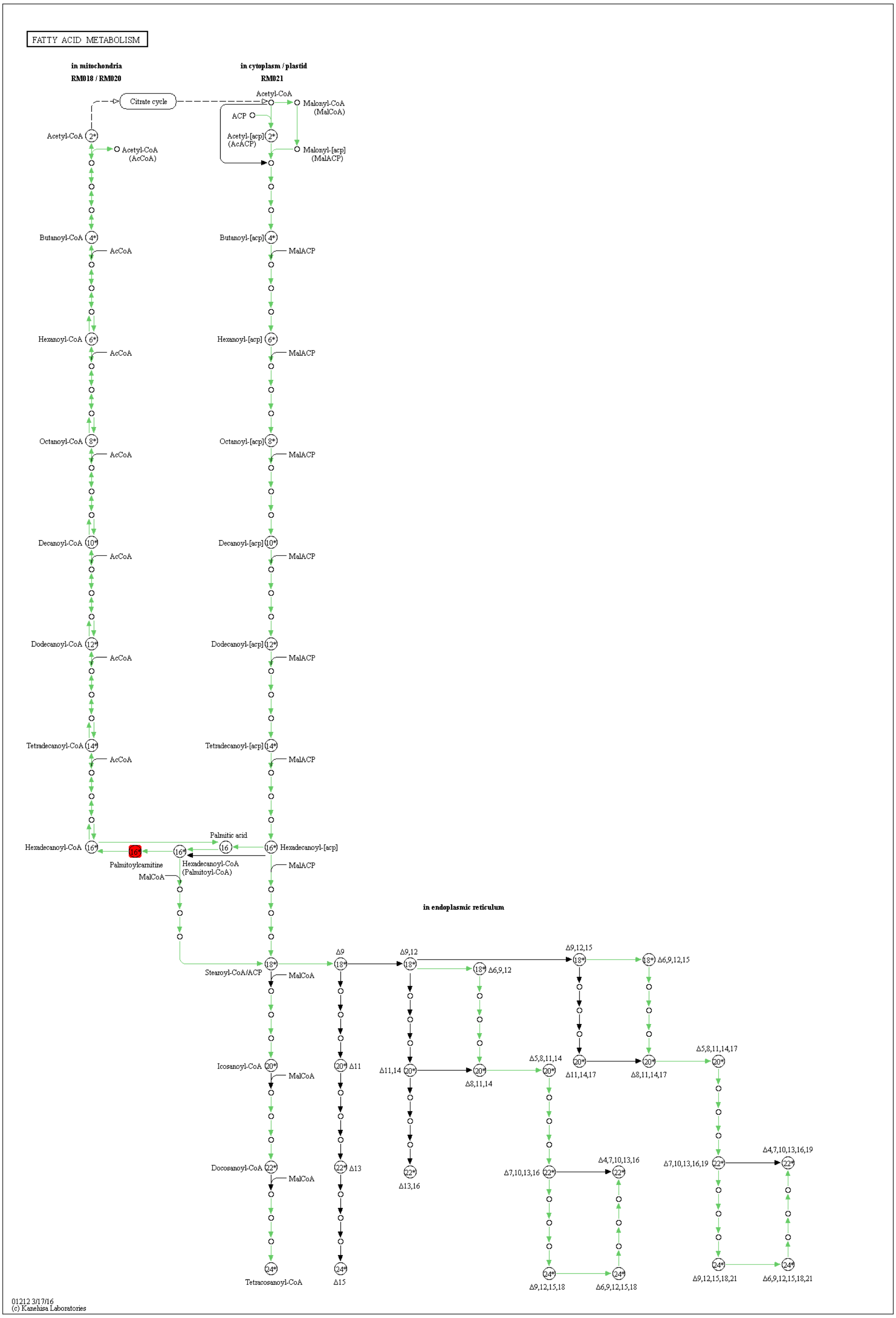

FATTY ACID METABOLISM
in mitochondria
RM018 / RM020
in cytoplasm / plastid
RM021
Citrate cycle
Acetyl-CoA
Malonyl-CoA
(MalCoA)
ACP
Acetyl-[acp]
(AcACP)
Malonyl-[acp]
(MalACP)
Acetyl-CoA
(AcCoA)
Butanoyl-CoA
Butanoyl-[acp]
AcCoA
MalACP
Hexanoyl-CoA
Hexanoyl-[acp]
Octanoyl-CoA
Octanoyl-[acp]
Decanoyl-CoA
Decanoyl-[acp]
Dodecanoyl-CoA
Dodecanoyl-[acp]
Tetradecanoyl-CoA
Tetradecanoyl-[acp]
Palmitic acid
Hexadecanoyl-CoA
Hexadecanoyl-[acp]
Palmitoylcarnitine
Hexadecanoyl-CoA
(Palmitoyl-CoA)
MalCoA
in endoplasmic reticulum
Stearoyl-CoA/ACP
Icosanoyl-CoA
Docosanoyl-CoA
Tetracosanoyl-CoA
Δ9
Δ9,12
Δ6,9,12
Δ9,12,15
Δ6,9,12,15
Δ11
Δ11,14
Δ8,11,14
Δ5,8,11,14
Δ11,14,17
Δ8,11,14,17
Δ5,8,11,14,17
Δ13
Δ13,16
Δ7,10,13,16
Δ4,7,10,13,16
Δ7,10,13,16,19
Δ4,7,10,13,16,19
Δ15
Δ9,12,15,18
Δ6,9,12,15,18
Δ9,12,15,18,21
Δ6,9,12,15,18,21
01212 3/17/16
(c) Kanehisa Laboratories

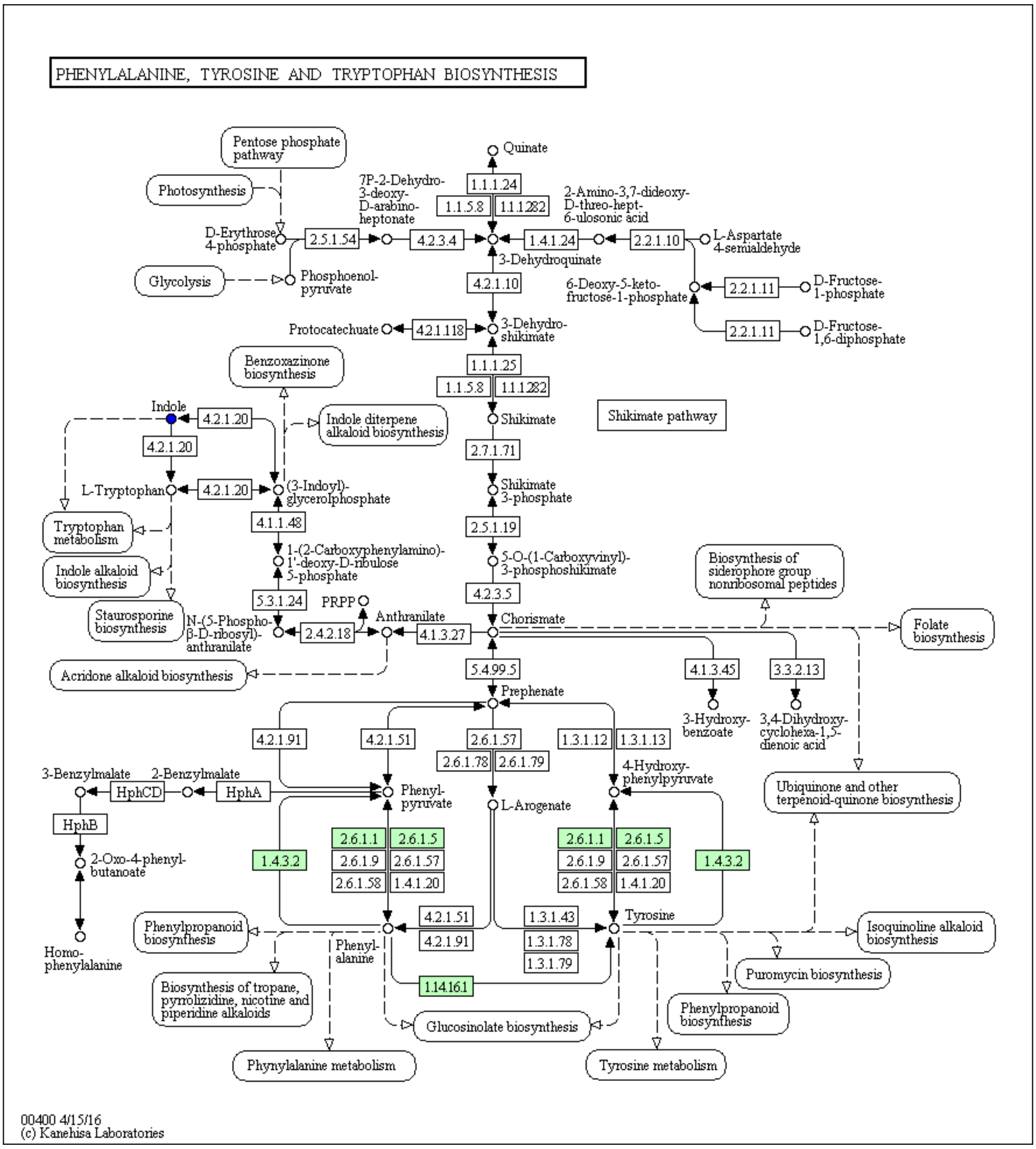
PHENYLALANINE, TYROSINE AND TRYPTOPHAN BIOSYNTHESIS
Pentose phosphate pathway
Photosynthesis
Glycolysis
D-Erythrose 4-phosphate
Phosphoenol-pyruvate
2.5.1.54
7P-2-Dehydro-3-deoxy-D-arabino-heptonate
4.2.3.4
Quinate
1.1.1.24
1.1.5.8
1.1.1.282
3-Dehydroquinate
1.4.1.24
2-Amino-3,7-dideoxy-D-threo-hept-6-ulosonic acid
2.2.1.10
L-Aspartate 4-semialdehyde
6-Deoxy-5-keto-fructose-1-phosphate
2.2.1.11
D-Fructose-1-phosphate
D-Fructose-1,6-diphosphate
4.2.1.10
Protocatechuate
4.2.1.118
3-Dehydro-shikimate
1.1.1.25
Shikimate
Shikimate pathway
2.7.1.71
Shikimate 3-phosphate
2.5.1.19
5-O-(1-Carboxyvinyl)-3-phosphoshikimate
4.2.3.5
Chorismate
Benzoxazinone biosynthesis
Indole diterpene alkaloid biosynthesis
Indole
4.2.1.20
L-Tryptophan
(3-Indoyl)-glycerolphosphate
Tryptophan metabolism
Indole alkaloid biosynthesis
Staurosporine biosynthesis
4.1.1.48
1-(2-Carboxyphenylamino)-1'-deoxy-D-ribulose 5-phosphate
5.3.1.24
PRPP
N-(5-Phospho-β-D-ribosyl)-anthranilate
2.4.2.18
Anthranilate
4.1.3.27
Acridone alkaloid biosynthesis
Biosynthesis of siderophore group nonribosomal peptides
Folate biosynthesis
4.1.3.45
3.3.2.13
3-Hydroxy-benzoate
3,4-Dihydroxy-cyclohexa-1,5-dienoic acid
5.4.99.5
Prephenate
4.2.1.91
4.2.1.51
2.6.1.57
2.6.1.78
2.6.1.79
1.3.1.12
1.3.1.13
4-Hydroxy-phenylpyruvate
Ubiquinone and other terpenoid-quinone biosynthesis
3-Benzylmalate
2-Benzylmalate
HphCD
HphA
HphB
2-Oxo-4-phenyl-butanoate
Homo-phenylalanine
Phenyl-pyruvate
L-Arogenate
1.4.3.2
2.6.1.1
2.6.1.5
2.6.1.9
2.6.1.58
1.4.1.20
1.3.1.43
1.3.1.78
1.3.1.79
Tyrosine
Phenylpropanoid biosynthesis
Phenyl-alanine
1.14.16.1
Isoquinoline alkaloid biosynthesis
Puromycin biosynthesis
Biosynthesis of tropane, pyrrolizidine, nicotine and piperidine alkaloids
Glucosinolate biosynthesis
Phynylalanine metabolism
Tyrosine metabolism
00400 4/15/16
(c) Kanehisa Laboratories

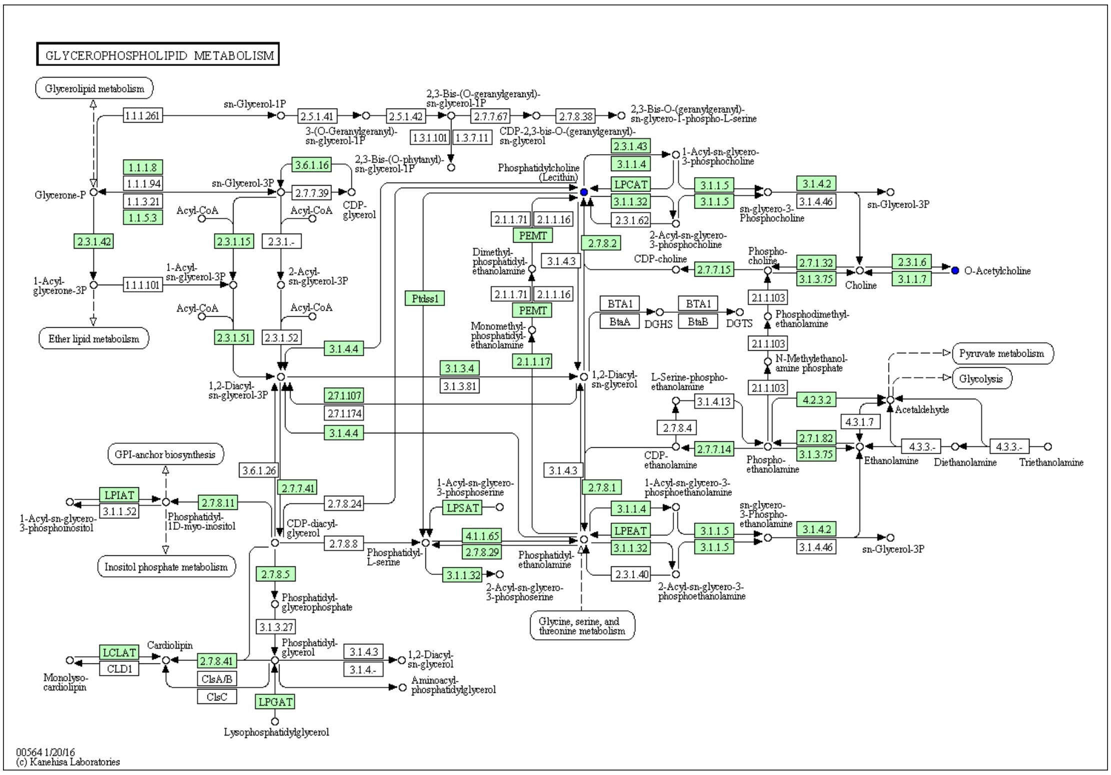
GLYCEROPHOSPHOLIPID METABOLISM
Glycerolipid metabolism
Glycerone-P
sn-Glycerol-1P
sn-Glycerol-3P
3-(O-Geranylgeranyl)-sn-glycerol-1P
2,3-Bis-(O-geranylgeranyl)-sn-glycerol-1P
CDP-2,3-bis-O-(geranylgeranyl)-sn-glycerol
2,3-Bis-O-(geranylgeranyl)-sn-glycero-1-phospho-L-serine
2,3-Bis-(O-phytanyl)-sn-glycerol-1P
CDP-glycerol
Acyl-CoA
1-Acyl-glycerone-3P
1-Acyl-sn-glycerol-3P
2-Acyl-sn-glycerol-3P
Ether lipid metabolism
1,2-Diacyl-sn-glycerol-3P
Phosphatidylcholine (Lecithin)
1-Acyl-sn-glycero-3-phosphocholine
2-Acyl-sn-glycero-3-phosphocholine
sn-glycero-3-Phosphocholine
sn-Glycerol-3P
Phospho-choline
Choline
O-Acetylcholine
CDP-choline
Dimethyl-phosphatidyl-ethanolamine
Monomethyl-phosphatidyl-ethanolamine
DGHS
DGTS
Phosphodimethyl-ethanolamine
N-Methylethanol-amine phosphate
Pyruvate metabolism
Glycolysis
Acetaldehyde
1,2-Diacyl-sn-glycerol
L-Serine-phospho-ethanolamine
CDP-ethanolamine
Phospho-ethanolamine
Ethanolamine
Diethanolamine
Triethanolamine
GPI-anchor biosynthesis
1-Acyl-sn-glycero-3-phosphoinositol
Phosphatidyl-1D-myo-inositol
Inositol phosphate metabolism
CDP-diacyl-glycerol
1-Acyl-sn-glycero-3-phosphoserine
Phosphatidyl-L-serine
2-Acyl-sn-glycero-3-phosphoserine
Phosphatidyl-ethanolamine
1-Acyl-sn-glycero-3-phosphoethanolamine
2-Acyl-sn-glycero-3-phosphoethanolamine
sn-glycero-3-Phospho-ethanolamine
Glycine, serine, and threonine metabolism
Phosphatidyl-glycerophosphate
Phosphatidyl-glycerol
1,2-Diacyl-sn-glycerol
Aminoacyl-phosphatidylglycerol
Cardiolipin
Monolyso-cardiolipin
Lysophosphatidylglycerol
1.1.1.261
2.5.1.41
2.5.1.42
2.7.7.67
2.7.8.38
1.3.1.101
1.3.7.11
1.1.1.8
1.1.1.94
1.1.3.21
1.1.5.3
3.6.1.16
2.7.7.39
2.3.1.42
2.3.1.15
2.3.1.-
1.1.1.101
2.3.1.51
2.3.1.52
3.1.4.4
2.3.1.43
3.1.1.4
LPCAT
3.1.1.32
2.3.1.62
3.1.1.5
3.1.4.2
3.1.4.46
2.1.1.71
2.1.1.16
PEMT
2.7.8.2
3.1.4.3
2.7.7.15
2.7.1.32
3.1.3.75
2.3.1.6
3.1.1.7
Ptdss1
BTA1
BtaA
BtaB
2.1.1.103
2.1.1.17
3.1.3.4
3.1.3.81
2.7.1.107
2.7.1.174
3.1.4.13
2.7.8.4
4.2.3.2
4.3.1.7
4.3.3.-
2.7.7.14
2.7.1.82
3.6.1.26
2.7.7.41
2.7.8.24
2.7.8.1
LPIAT
3.1.1.52
2.7.8.11
LPSAT
4.1.1.65
2.7.8.29
2.7.8.8
LPEAT
2.7.8.5
3.1.3.27
LCLAT
CLD1
2.7.8.41
ClsA/B
ClsC
3.1.4.-
LPGAT
00564 1/20/16
(c) Kanehisa Laboratories

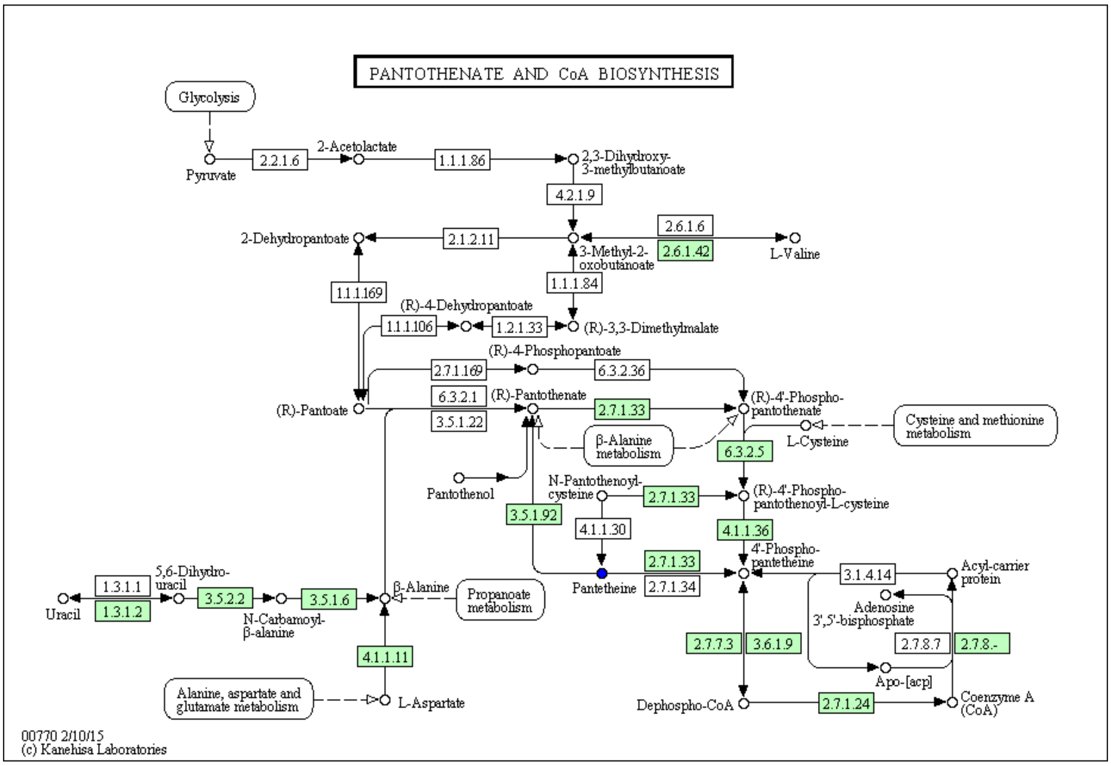
PANTOTHENATE AND CoA BIOSYNTHESIS
Glycolysis
Pyruvate
2.2.1.6
2-Acetolactate
1.1.1.86
2,3-Dihydroxy-3-methylbutanoate
4.2.1.9
2-Dehydropantoate
2.1.2.11
3-Methyl-2-oxobutanoate
2.6.1.6
2.6.1.42
L-Valine
1.1.1.169
1.1.1.84
(R)-4-Dehydropantoate
1.1.1.106
1.2.1.33
(R)-3,3-Dimethylmalate
(R)-4-Phosphopantoate
2.7.1.169
6.3.2.36
(R)-Pantoate
6.3.2.1
3.5.1.22
(R)-Pantothenate
2.7.1.33
(R)-4'-Phospho-pantothenate
Cysteine and methionine metabolism
L-Cysteine
β-Alanine metabolism
6.3.2.5
Pantothenol
N-Pantothenoyl-cysteine
2.7.1.33
(R)-4'-Phospho-pantothenoyl-L-cysteine
3.5.1.92
4.1.1.30
4.1.1.36
4'-Phospho-pantetheine
2.7.1.33
Pantetheine
2.7.1.34
3.1.4.14
Acyl-carrier protein
Adenosine 3',5'-bisphosphate
1.3.1.1
5,6-Dihydro-uracil
Uracil
1.3.1.2
3.5.2.2
3.5.1.6
N-Carbamoyl-β-alanine
β-Alanine
Propanoate metabolism
2.7.7.3
3.6.1.9
2.7.8.7
2.7.8.-
Apo-[acp]
4.1.1.11
Alanine, aspartate and glutamate metabolism
L-Aspartate
Dephospho-CoA
2.7.1.24
Coenzyme A (CoA)
00770 2/10/15
(c) Kanehisa Laboratories

SA vs. PR-ACS

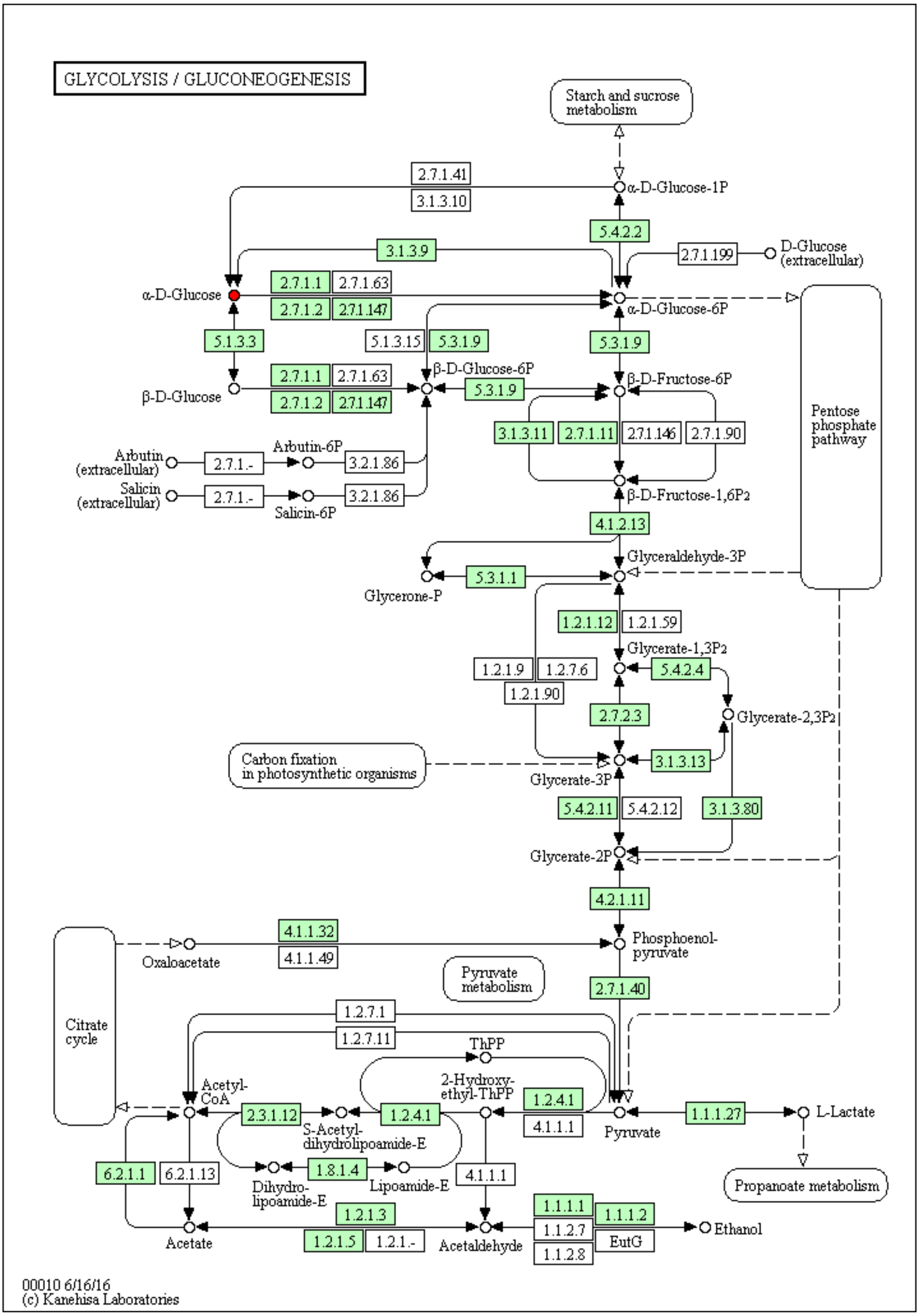
GLYCOLYSIS / GLUCONEOGENESIS
Starch and sucrose metabolism
2.7.1.41
3.1.3.10
α-D-Glucose-1P
5.4.2.2
3.1.3.9
2.7.1.199
D-Glucose (extracellular)
α-D-Glucose
2.7.1.1
2.7.1.63
2.7.1.2
2.7.1.147
α-D-Glucose-6P
5.1.3.3
5.1.3.15
5.3.1.9
β-D-Glucose-6P
β-D-Glucose
β-D-Fructose-6P
3.1.3.11
2.7.1.11
2.7.1.146
2.7.1.90
Pentose phosphate pathway
Arbutin (extracellular)
2.7.1.-
Arbutin-6P
3.2.1.86
Salicin (extracellular)
Salicin-6P
β-D-Fructose-1,6P2
4.1.2.13
Glyceraldehyde-3P
5.3.1.1
Glycerone-P
1.2.1.12
1.2.1.59
Glycerate-1,3P2
1.2.1.9
1.2.7.6
1.2.1.90
5.4.2.4
2.7.2.3
Glycerate-2,3P2
Carbon fixation in photosynthetic organisms
3.1.3.13
Glycerate-3P
5.4.2.11
5.4.2.12
3.1.3.80
Glycerate-2P
4.2.1.11
4.1.1.32
4.1.1.49
Oxaloacetate
Phosphoenol-pyruvate
Citrate cycle
Pyruvate metabolism
2.7.1.40
1.2.7.1
1.2.7.11
ThPP
Acetyl-CoA
2-Hydroxy-ethyl-ThPP
1.2.4.1
2.3.1.12
S-Acetyl-dihydrolipoamide-E
4.1.1.1
Pyruvate
1.1.1.27
L-Lactate
6.2.1.1
6.2.1.13
1.8.1.4
Dihydro-lipoamide-E
Lipoamide-E
Propanoate metabolism
1.2.1.3
1.2.1.5
1.2.1.-
Acetate
Acetaldehyde
1.1.1.1
1.1.2.7
1.1.2.8
1.1.1.2
EutG
Ethanol
00010 6/16/16
(c) Kanehisa Laboratories

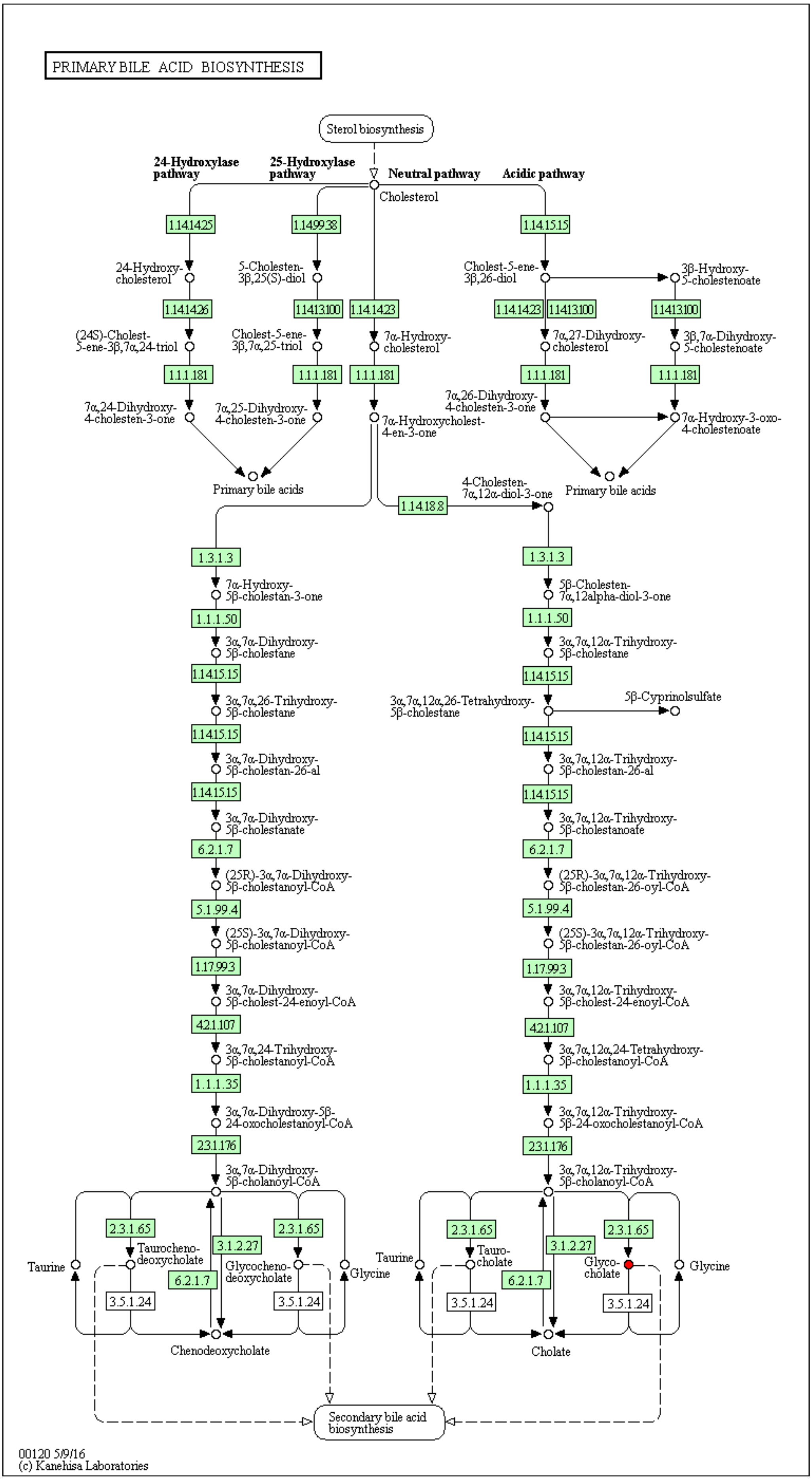

PRIMARY BILE ACID BIOSYNTHESIS
Sterol biosynthesis
24-Hydroxylase pathway
25-Hydroxylase pathway
Neutral pathway
Acidic pathway
Cholesterol
1.14.14.25
1.14.99.38
1.14.15.15
24-Hydroxy-cholesterol
5-Cholesten-3β,25(S)-diol
Cholest-5-ene-3β,26-diol
3β-Hydroxy-5-cholestenoate
1.14.14.26
1.14.13.100
1.14.14.23
1.14.14.23
1.14.13.100
1.14.13.100
(24S)-Cholest-5-ene-3β,7α,24-triol
Cholest-5-ene-3β,7α,25-triol
7α-Hydroxy-cholesterol
7α,27-Dihydroxy-cholesterol
3β,7α-Dihydroxy-5-cholestenoate
1.1.1.181
7α,24-Dihydroxy-4-cholesten-3-one
7α,25-Dihydroxy-4-cholesten-3-one
7α-Hydroxycholest-4-en-3-one
7α,26-Dihydroxy-4-cholesten-3-one
7α-Hydroxy-3-oxo-4-cholestenoate
Primary bile acids
Primary bile acids
1.14.18.8
4-Cholesten-7α,12α-diol-3-one
1.3.1.3
7α-Hydroxy-5β-cholestan-3-one
5β-Cholesten-7α,12alpha-diol-3-one
1.1.1.50
3α,7α-Dihydroxy-5β-cholestane
3α,7α,12α-Trihydroxy-5β-cholestane
1.14.15.15
3α,7α,26-Trihydroxy-5β-cholestane
3α,7α,12α,26-Tetrahydroxy-5β-cholestane
5β-Cyprinolsulfate
3α,7α-Dihydroxy-5β-cholestan-26-al
3α,7α,12α-Trihydroxy-5β-cholestan-26-al
3α,7α-Dihydroxy-5β-cholestanate
3α,7α,12α-Trihydroxy-5β-cholestanoate
6.2.1.7
(25R)-3α,7α-Dihydroxy-5β-cholestanoyl-CoA
(25R)-3α,7α,12α-Trihydroxy-5β-cholestan-26-oyl-CoA
5.1.99.4
(25S)-3α,7α-Dihydroxy-5β-cholestanoyl-CoA
(25S)-3α,7α,12α-Trihydroxy-5β-cholestan-26-oyl-CoA
1.17.99.3
3α,7α-Dihydroxy-5β-cholest-24-enoyl-CoA
3α,7α,12α-Trihydroxy-5β-cholest-24-enoyl-CoA
4.2.1.107
3α,7α,24-Trihydroxy-5β-cholestanoyl-CoA
3α,7α,12α,24-Tetrahydroxy-5β-cholestanoyl-CoA
1.1.1.35
3α,7α-Dihydroxy-5β-24-oxocholestanoyl-CoA
3α,7α,12α-Trihydroxy-5β-24-oxocholestanoyl-CoA
2.3.1.176
3α,7α-Dihydroxy-5β-cholanoyl-CoA
3α,7α,12α-Trihydroxy-5β-cholanoyl-CoA
2.3.1.65
3.1.2.27
Taurine
Taurocheno-deoxycholate
Glycocheno-deoxycholate
Glycine
Tauro-cholate
Glyco-cholate
3.5.1.24
Chenodeoxycholate
Cholate
Secondary bile acid biosynthesis
00120 5/9/16
(c) Kanehisa Laboratories

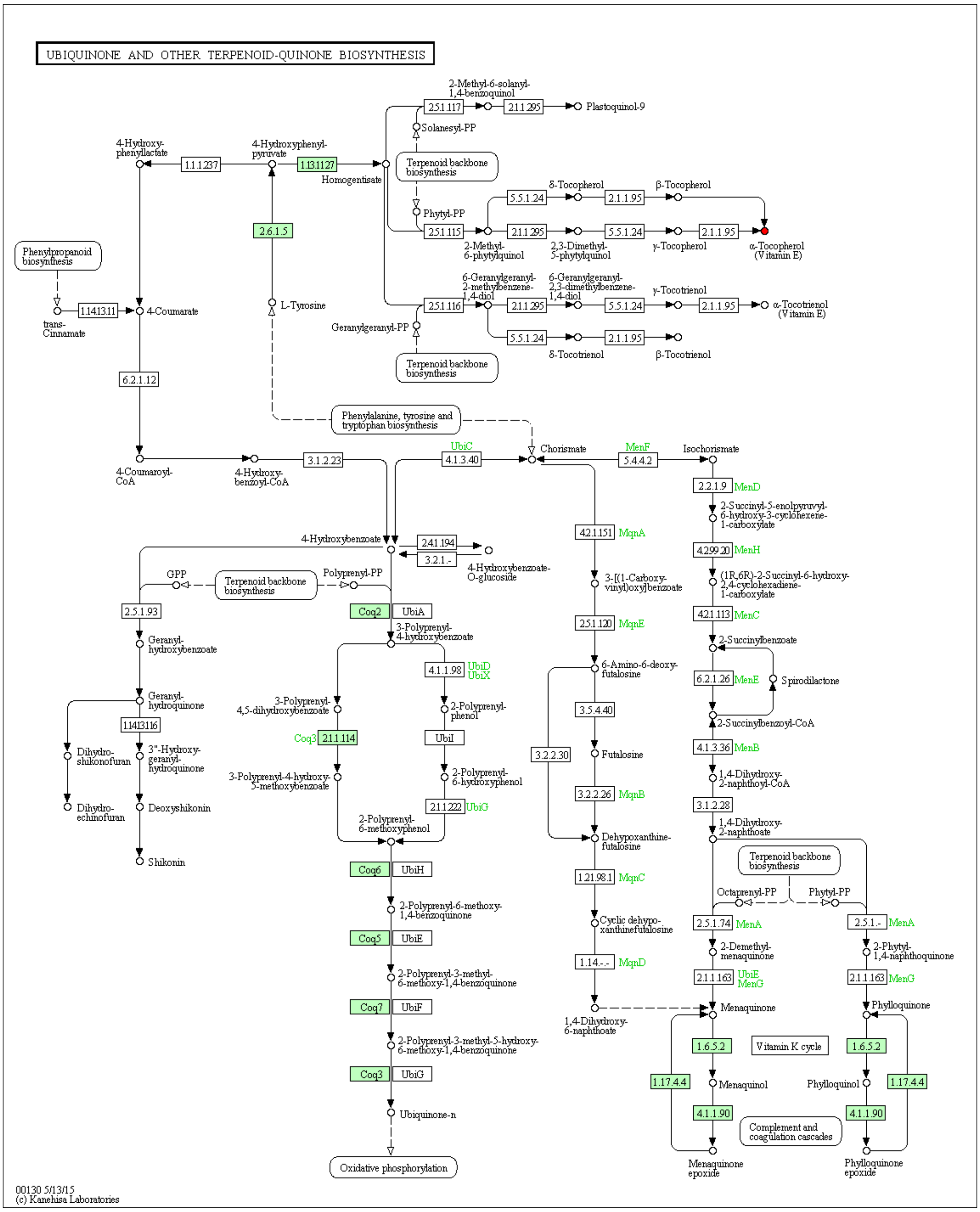
UBIQUINONE AND OTHER TERPENOID-QUINONE BIOSYNTHESIS
2-Methyl-6-solanyl-1,4-benzoquinol
2.5.1.117
2.1.1.295
Plastoquinol-9
Solanesyl-PP
4-Hydroxyphenyllactate
1.1.1.237
4-Hydroxyphenylpyruvate
1.13.11.27
Homogentisate
Terpenoid backbone biosynthesis
δ-Tocopherol
β-Tocopherol
5.5.1.24
2.1.1.95
Phytyl-PP
2.6.1.5
2.5.1.115
2-Methyl-6-phytylquinol
2,3-Dimethyl-5-phytylquinol
γ-Tocopherol
α-Tocopherol (Vitamin E)
Phenylpropanoid biosynthesis
6-Geranylgeranyl-2-methylbenzene-1,4-diol
6-Geranylgeranyl-2,3-dimethylbenzene-1,4-diol
γ-Tocotrienol
L-Tyrosine
2.5.1.116
α-Tocotrienol (Vitamin E)
trans-Cinnamate
1.14.13.11
4-Coumarate
Geranylgeranyl-PP
δ-Tocotrienol
β-Tocotrienol
6.2.1.12
Phenylalanine, tyrosine and tryptophan biosynthesis
UbiC
MenF
Chorismate
Isochorismate
4.1.3.40
5.4.4.2
4-Coumaroyl-CoA
4-Hydroxybenzoyl-CoA
3.1.2.23
2.2.1.9
MenD
2-Succinyl-5-enolpyruvyl-6-hydroxy-3-cyclohexene-1-carboxylate
4-Hydroxybenzoate
2.4.1.194
3.2.1.-
4-Hydroxybenzoate-O-glucoside
4.2.1.151
MqnA
4.2.99.20
MenH
GPP
Polyprenyl-PP
3-[(1-Carboxyvinyl)oxy]benzoate
(1R,6R)-2-Succinyl-6-hydroxy-2,4-cyclohexadiene-1-carboxylate
2.5.1.93
Coq2
UbiA
3-Polyprenyl-4-hydroxybenzoate
2.5.1.120
MqnE
4.2.1.113
MenC
Geranylhydroxybenzoate
2-Succinylbenzoate
4.1.1.98
UbiD
UbiX
6-Amino-6-deoxyfutalosine
Geranylhydroquinone
6.2.1.26
MenE
Spirodilactone
1.14.13.116
3-Polyprenyl-4,5-dihydroxybenzoate
2-Polyprenylphenol
3.5.4.40
2-Succinylbenzoyl-CoA
Dihydroshikonofuran
3''-Hydroxygeranylhydroquinone
Coq3
2.1.1.114
UbiI
3.2.2.30
Futalosine
4.1.3.36
MenB
3-Polyprenyl-4-hydroxy-5-methoxybenzoate
2-Polyprenyl-6-hydroxyphenol
3.2.2.26
MqnB
1,4-Dihydroxy-2-naphthoyl-CoA
Dihydroechinofuran
Deoxyshikonin
2.1.1.222
UbiG
3.1.2.28
2-Polyprenyl-6-methoxyphenol
Dehypoxanthinefutalosine
1,4-Dihydroxy-2-naphthoate
Shikonin
Coq6
UbiH
1.21.98.1
MqnC
Octaprenyl-PP
2-Polyprenyl-6-methoxy-1,4-benzoquinone
Cyclic dehypoxanthinefutalosine
2.5.1.74
MenA
2.5.1.-
Coq5
UbiE
2-Demethylmenaquinone
2-Phytyl-1,4-naphthoquinone
1.14.-.-
MqnD
2-Polyprenyl-3-methyl-6-methoxy-1,4-benzoquinone
2.1.1.163
UbiE
MenG
Coq7
UbiF
Menaquinone
Phylloquinone
1,4-Dihydroxy-6-naphthoate
2-Polyprenyl-3-methyl-5-hydroxy-6-methoxy-1,4-benzoquinone
1.6.5.2
Vitamin K cycle
1.17.4.4
Coq3
UbiG
Menaquinol
Phylloquinol
4.1.1.90
Ubiquinone-n
Complement and coagulation cascades
Oxidative phosphorylation
Menaquinone epoxide
Phylloquinone epoxide
00130 5/13/15
(c) Kanehisa Laboratories

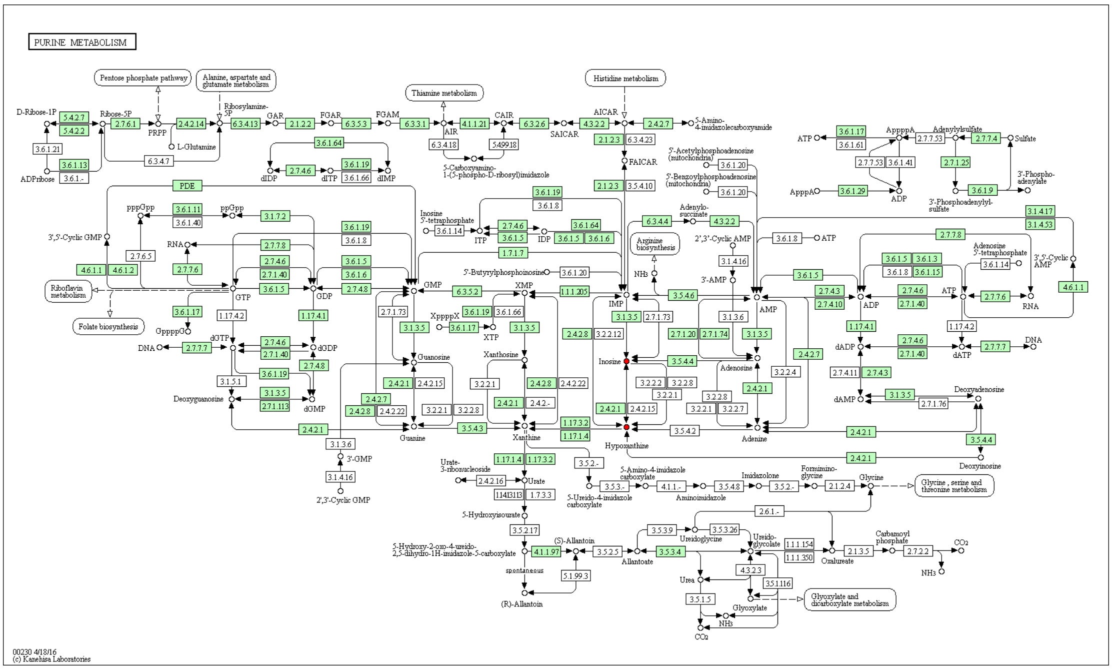
PURINE METABOLISM
Pentose phosphate pathway
Alanine, aspartate and glutamate metabolism
Thiamine metabolism
Histidine metabolism
00230 4/18/16
(c) Kanehisa Laboratories

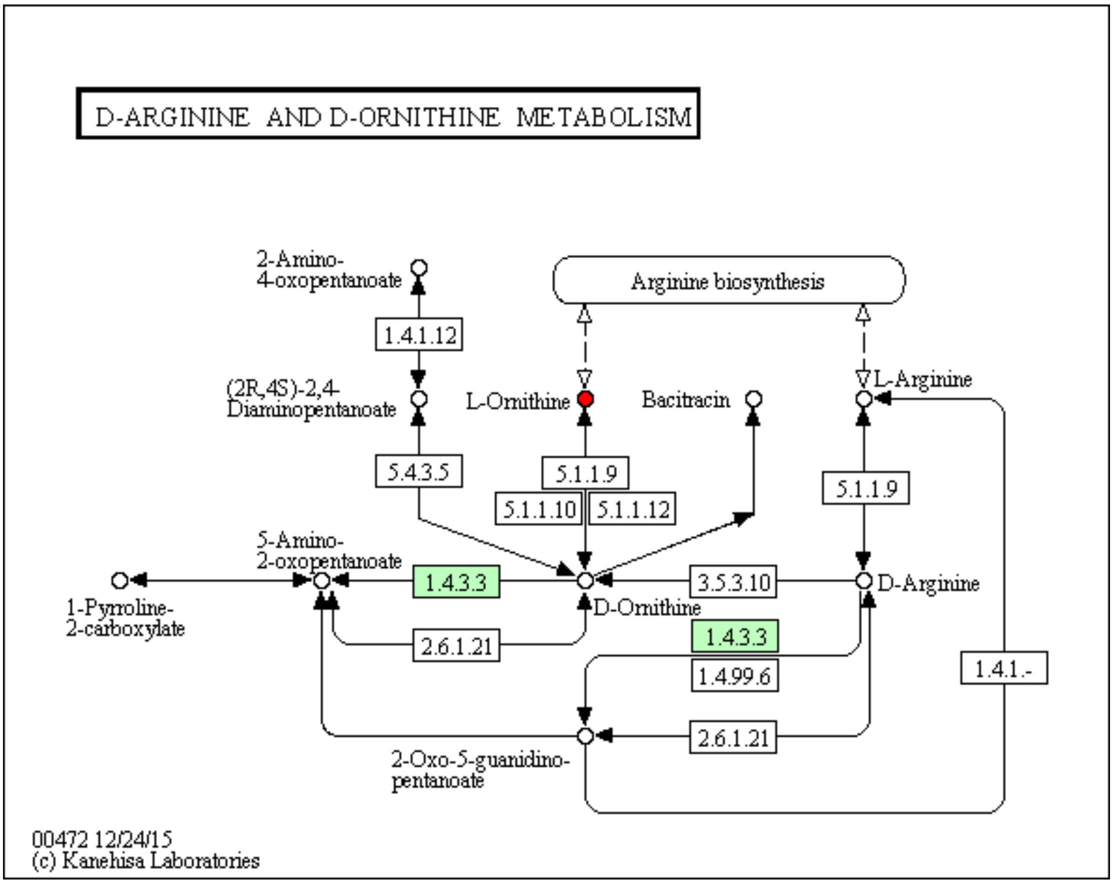
D-ARGININE AND D-ORNITHINE METABOLISM
2-Amino-
4-oxopentanoate
Arginine biosynthesis
1.4.1.12
(2R,4S)-2,4-
Diaminopentanoate
L-Ornithine
Bacitracin
L-Arginine
5.4.3.5
5.1.1.9
5.1.1.10
5.1.1.12
5.1.1.9
5-Amino-
2-oxopentanoate
1-Pyrroline-
2-carboxylate
1.4.3.3
D-Ornithine
3.5.3.10
D-Arginine
2.6.1.21
1.4.3.3
1.4.99.6
1.4.1.-
2.6.1.21
2-Oxo-5-guanidino-
pentanoate
00472 12/24/15
(c) Kanehisa Laboratories

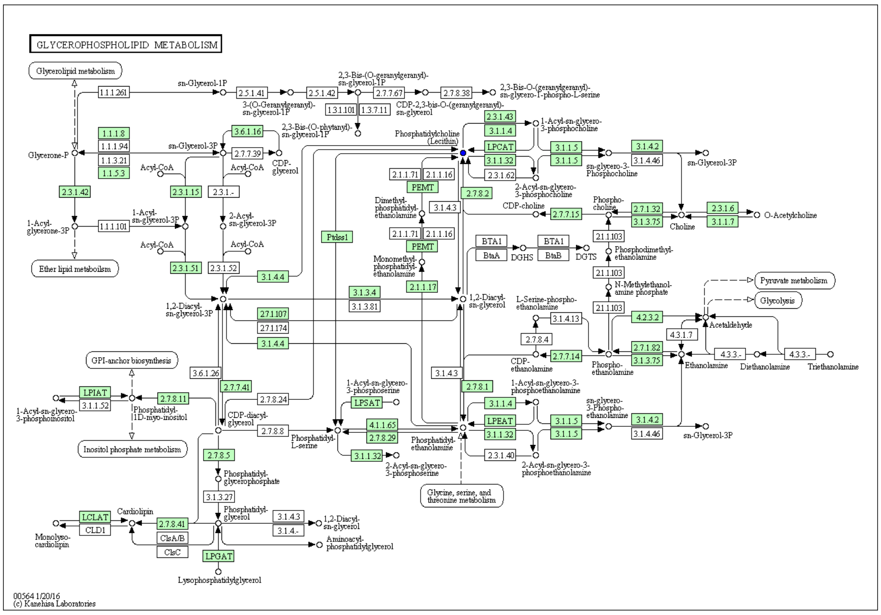
GLYCEROPHOSPHOLIPID METABOLISM
Glycerolipid metabolism
sn-Glycerol-1P
Glycerone-P
sn-Glycerol-3P
1-Acyl-glycerone-3P
Ether lipid metabolism
1-Acyl-sn-glycerol-3P
2-Acyl-sn-glycerol-3P
1,2-Diacyl-sn-glycerol-3P
CDP-diacylglycerol
Phosphatidylcholine (Lecithin)
Dimethyl-phosphatidyl-ethanolamine
Monomethyl-phosphatidyl-ethanolamine
1,2-Diacyl-sn-glycerol
CDP-choline
Phosphocholine
Choline
O-Acetylcholine
sn-Glycero-3-Phosphocholine
sn-Glycerol-3P
Pyruvate metabolism
Glycolysis
Acetaldehyde
Ethanolamine
Diethanolamine
Triethanolamine
CDP-ethanolamine
Phosphoethanolamine
L-Serine-phospho-ethanolamine
GPI-anchor biosynthesis
Phosphatidyl-1D-myo-inositol
1-Acyl-sn-glycero-3-phosphoinositol
Inositol phosphate metabolism
Phosphatidyl-L-serine
Phosphatidylethanolamine
1-Acyl-sn-glycero-3-phosphoserine
2-Acyl-sn-glycero-3-phosphoserine
Phosphatidylglycerophosphate
Phosphatidylglycerol
Cardiolipin
Monolyso-cardiolipin
Aminoacyl-phosphatidylglycerol
Lysophosphatidylglycerol
Glycine, serine, and threonine metabolism
00564 1/20/16
(c) Kanehisa Laboratories

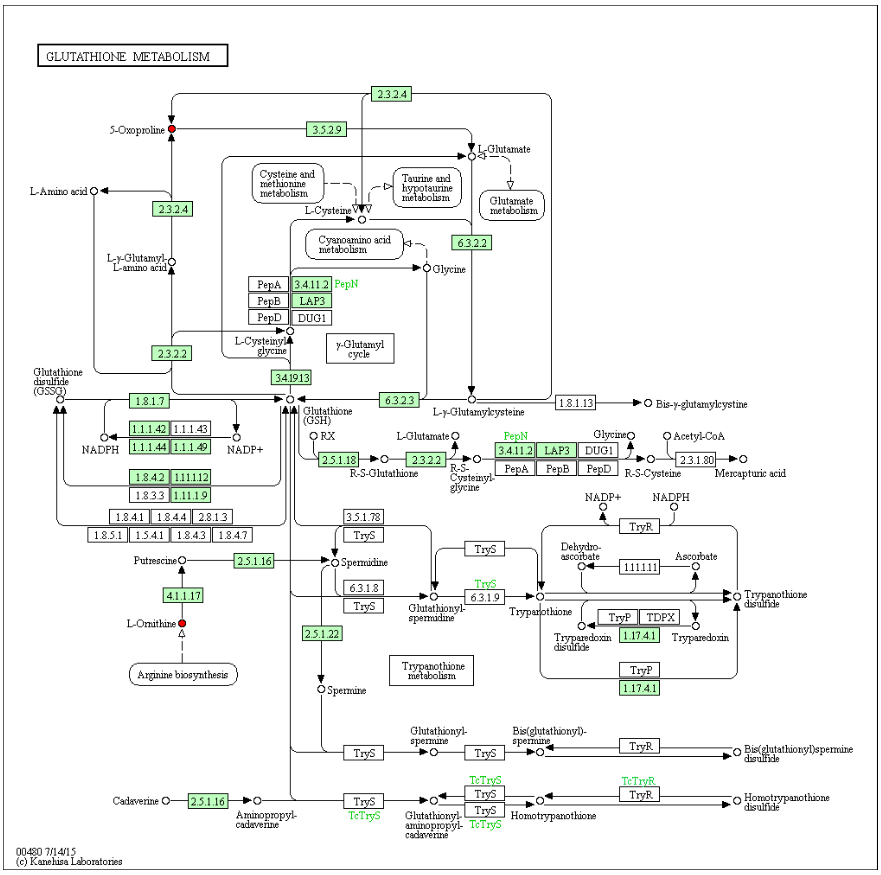
GLUTATHIONE METABOLISM
5-Oxoproline
L-Glutamate
L-Amino acid
Cysteine and methionine metabolism
Taurine and hypotaurine metabolism
Glutamate metabolism
L-Cysteine
Cyanoamino acid metabolism
Glycine
L-γ-Glutamyl-L-amino acid
L-Cysteinyl-glycine
γ-Glutamyl cycle
Glutathione disulfide (GSSG)
Glutathione (GSH)
L-γ-Glutamylcysteine
Bis-γ-glutamylcystine
NADPH
NADP+
RX
R-S-Glutathione
R-S-Cysteinyl-glycine
R-S-Cysteine
Acetyl-CoA
Mercapturic acid
Putrescine
Spermidine
L-Ornithine
Arginine biosynthesis
Glutathionyl-spermidine
Trypanothione
Trypanothione disulfide
Dehydro-ascorbate
Ascorbate
Tryparedoxin disulfide
Tryparedoxin
Trypanothione metabolism
Spermine
Glutathionyl-spermine
Bis(glutathionyl)-spermine
Bis(glutathionyl)spermine disulfide
Cadaverine
Aminopropyl-cadaverine
Glutathionyl-aminopropyl-cadaverine
Homotrypanothione
Homotrypanothione disulfide
00480 7/14/15
(c) Kanehisa Laboratories

PR-ACS vs. PO-ACS

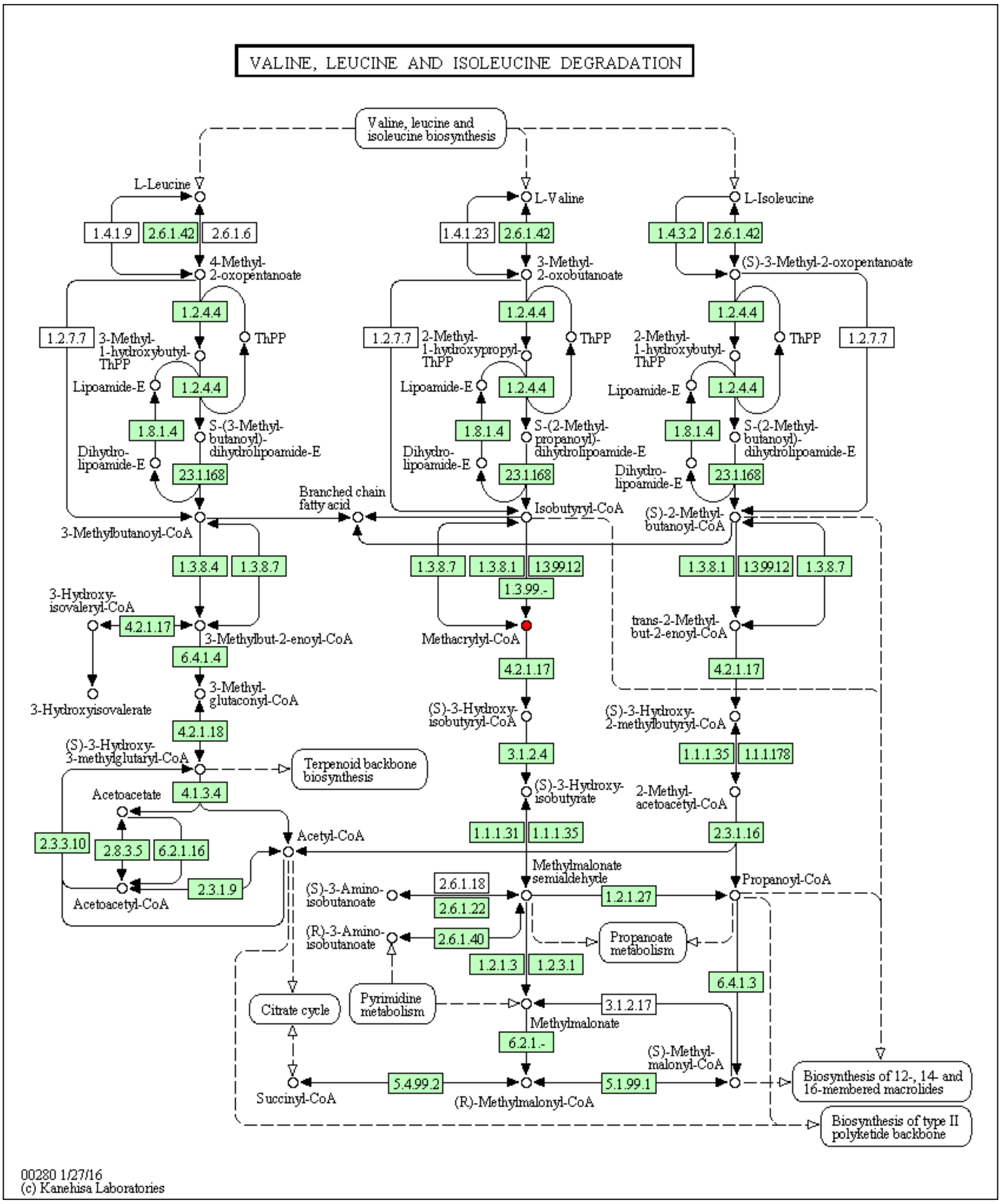

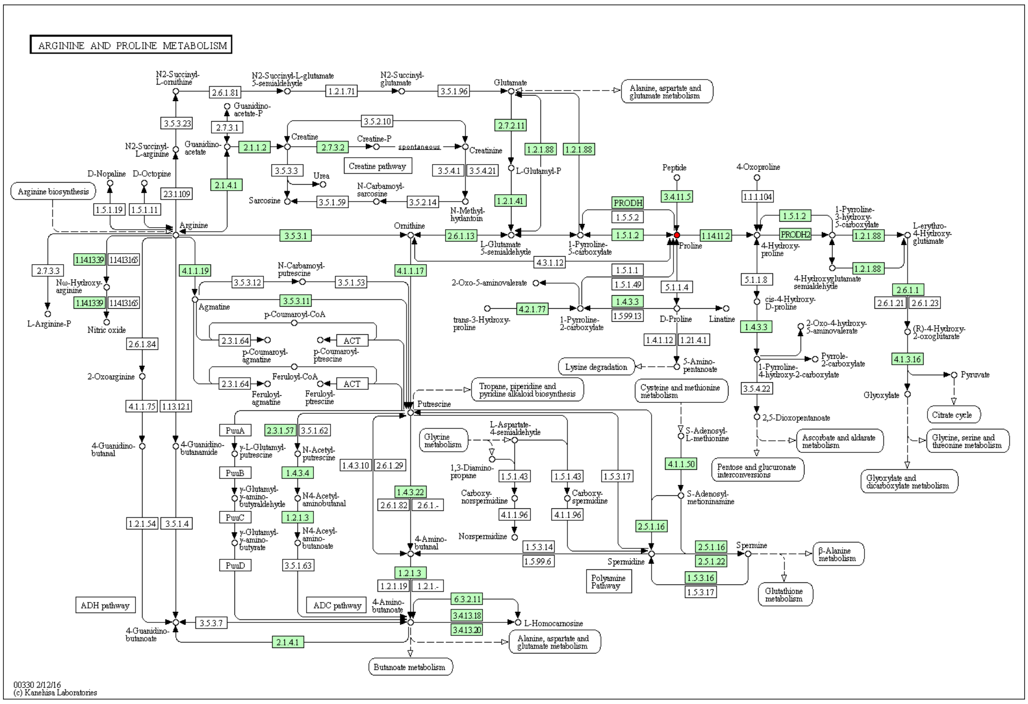
ARGININE AND PROLINE METABOLISM
Arginine biosynthesis
Glutamate
Alanine, aspartate and glutamate metabolism
Creatine pathway
Arginine
Ornithine
Proline
Putrescine
Agmatine
Spermidine
Spermine
Lysine degradation
Cysteine and methionine metabolism
Tropane, piperidine and pyridine alkaloid biosynthesis
Glycine metabolism
Ascorbate and aldarate metabolism
Pentose and glucuronate interconversions
Citrate cycle
Glycine, serine and threonine metabolism
Glyoxylate and dicarboxylate metabolism
β-Alanine metabolism
Glutathione metabolism
Polyamine Pathway
ADH pathway
ADC pathway
Butanoate metabolism
L-Homocarnosine
00330 2/12/16
(c) Kanehisa Laboratories

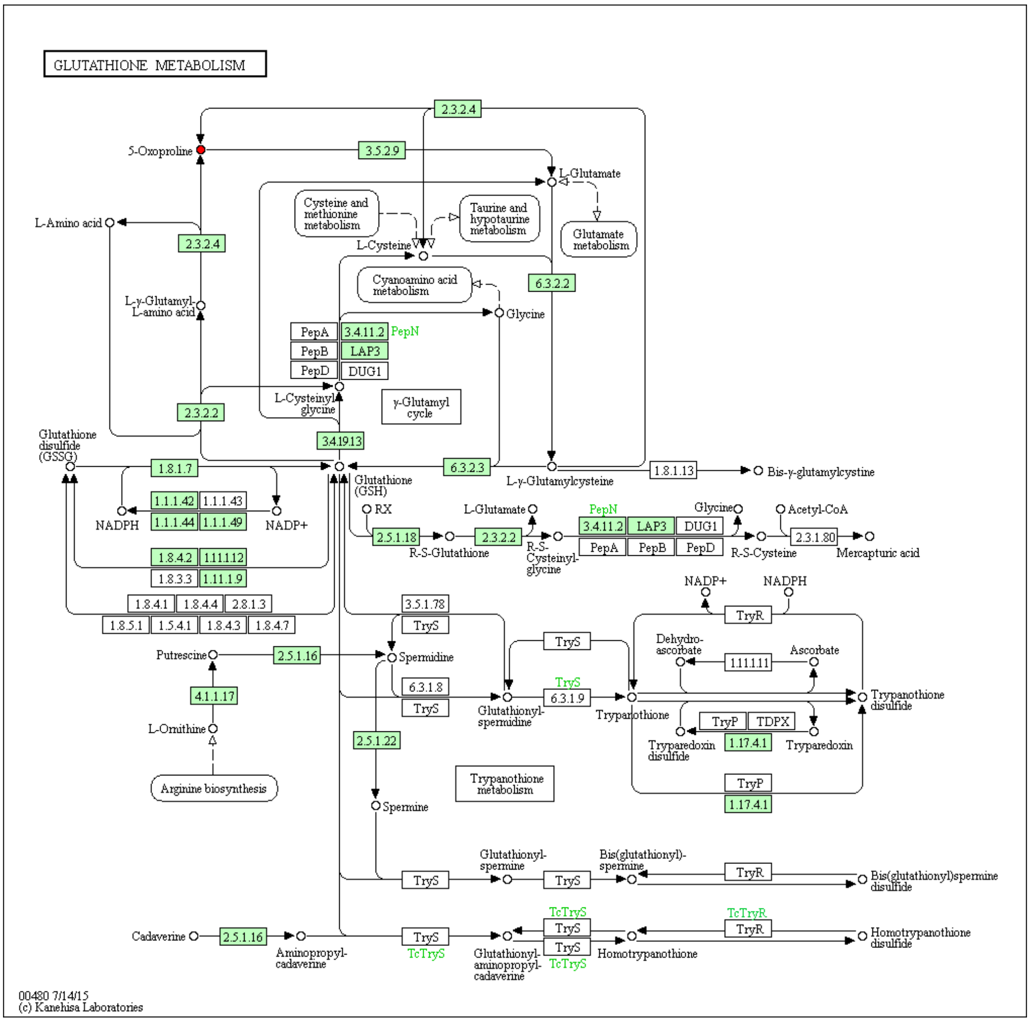
GLUTATHIONE METABOLISM
5-Oxoproline
2.3.2.4
3.5.2.9
L-Glutamate
L-Amino acid
Cysteine and methionine metabolism
Taurine and hypotaurine metabolism
Glutamate metabolism
L-Cysteine
Cyanoamino acid metabolism
6.3.2.2
L-γ-Glutamyl-L-amino acid
Glycine
PepA
3.4.11.2
PepN
PepB
LAP3
PepD
DUG1
L-Cysteinyl glycine
γ-Glutamyl cycle
2.3.2.2
3.4.19.13
Glutathione disulfide (GSSG)
1.8.1.7
6.3.2.3
Glutathione (GSH)
L-γ-Glutamylcysteine
1.8.1.13
Bis-γ-glutamylcystine
1.11.1.42
1.11.1.43
1.11.1.44
1.11.1.49
NADPH
NADP+
RX
2.5.1.18
R-S-Glutathione
R-S-Cysteinyl-glycine
Acetyl-CoA
2.3.1.80
R-S-Cysteine
Mercapturic acid
1.8.4.2
1.11.1.12
1.8.3.3
1.11.1.9
1.8.4.1
1.8.4.4
2.8.1.3
1.8.5.1
1.5.4.1
1.8.4.3
1.8.4.7
3.5.1.78
TryS
TryR
Putrescine
2.5.1.16
Spermidine
6.3.1.8
Dehydro-ascorbate
Ascorbate
1.11.1.11
Glutathionyl-spermidine
6.3.1.9
Trypanothione
Trypanothione disulfide
TryP
TDPX
Trypanedoxin disulfide
Trypanedoxin
1.17.4.1
4.1.1.17
L-Ornithine
2.5.1.22
Arginine biosynthesis
Trypanothione metabolism
Spermine
Glutathionyl-spermine
Bis(glutathionyl)-spermine
Bis(glutathionyl)spermine disulfide
Cadaverine
Aminopropyl-cadaverine
TcTryS
TcTryR
Glutathionyl-aminopropyl-cadaverine
Homotrypanothione
Homotrypanothione disulfide
00480 7/14/15
(c) Kanehisa Laboratories

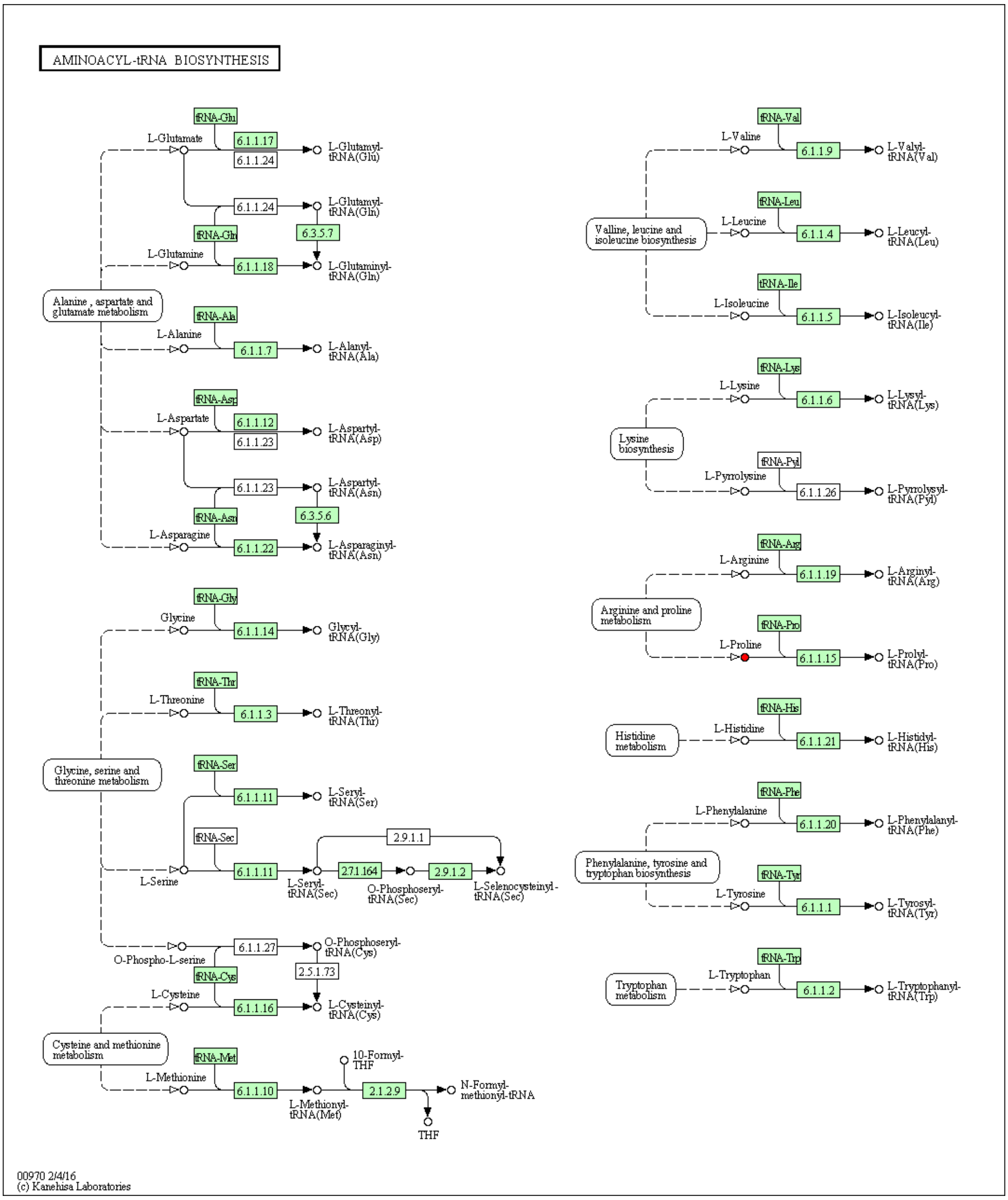

AMINOACYL-tRNA BIOSYNTHESIS
tRNA-Glu
L-Glutamate
6.1.1.17
6.1.1.24
L-Glutamyl-tRNA(Glu)
6.1.1.24
L-Glutamyl-tRNA(Gln)
tRNA-Gln
6.3.5.7
L-Glutamine
6.1.1.18
L-Glutaminyl-tRNA(Gln)
Alanine , aspartate and glutamate metabolism
tRNA-Ala
L-Alanine
6.1.1.7
L-Alanyl-tRNA(Ala)
tRNA-Asp
L-Aspartate
6.1.1.12
6.1.1.23
L-Aspartyl-tRNA(Asp)
6.1.1.23
L-Aspartyl-tRNA(Asn)
tRNA-Asn
6.3.5.6
L-Asparagine
6.1.1.22
L-Asparaginyl-tRNA(Asn)
tRNA-Gly
Glycine
6.1.1.14
Glycyl-tRNA(Gly)
tRNA-Thr
L-Threonine
6.1.1.3
L-Threonyl-tRNA(Thr)
Glycine, serine and threonine metabolism
tRNA-Ser
6.1.1.11
L-Seryl-tRNA(Ser)
tRNA-Sec
2.9.1.1
6.1.1.11
L-Serine
L-Seryl-tRNA(Sec)
2.7.1.164
O-Phosphoseryl-tRNA(Sec)
2.9.1.2
L-Selenocysteinyl-tRNA(Sec)
6.1.1.27
O-Phosphoseryl-tRNA(Cys)
O-Phospho-L-serine
2.5.1.73
tRNA-Cys
L-Cysteine
6.1.1.16
L-Cysteinyl-tRNA(Cys)
Cysteine and methionine metabolism
tRNA-Met
L-Methionine
6.1.1.10
L-Methionyl-tRNA(Met)
10-Formyl-THF
2.1.2.9
N-Formyl-methionyl-tRNA
THF
tRNA-Val
L-Valine
6.1.1.9
L-Valyl-tRNA(Val)
Valline, leucine and isoleucine biosynthesis
tRNA-Leu
L-Leucine
6.1.1.4
L-Leucyl-tRNA(Leu)
tRNA-Ile
L-Isoleucine
6.1.1.5
L-Isoleucyl-tRNA(Ile)
tRNA-Lys
L-Lysine
6.1.1.6
L-Lysyl-tRNA(Lys)
Lysine biosynthesis
tRNA-Pyl
L-Pyrrolysine
6.1.1.26
L-Pyrrolysyl-tRNA(Pyl)
tRNA-Arg
L-Arginine
6.1.1.19
L-Arginyl-tRNA(Arg)
Arginine and proline metabolism
tRNA-Pro
L-Proline
6.1.1.15
L-Prolyl-tRNA(Pro)
tRNA-His
Histidine metabolism
L-Histidine
6.1.1.21
L-Histidyl-tRNA(His)
tRNA-Phe
L-Phenylalanine
6.1.1.20
L-Phenylalanyl-tRNA(Phe)
Phenylalanine, tyrosine and tryptophan biosynthesis
tRNA-Tyr
L-Tyrosine
6.1.1.1
L-Tyrosyl-tRNA(Tyr)
tRNA-Trp
Tryptophan metabolism
L-Tryptophan
6.1.1.2
L-Tryptophanyl-tRNA(Trp)
00970 2/4/16
(c) Kanehisa Laboratories

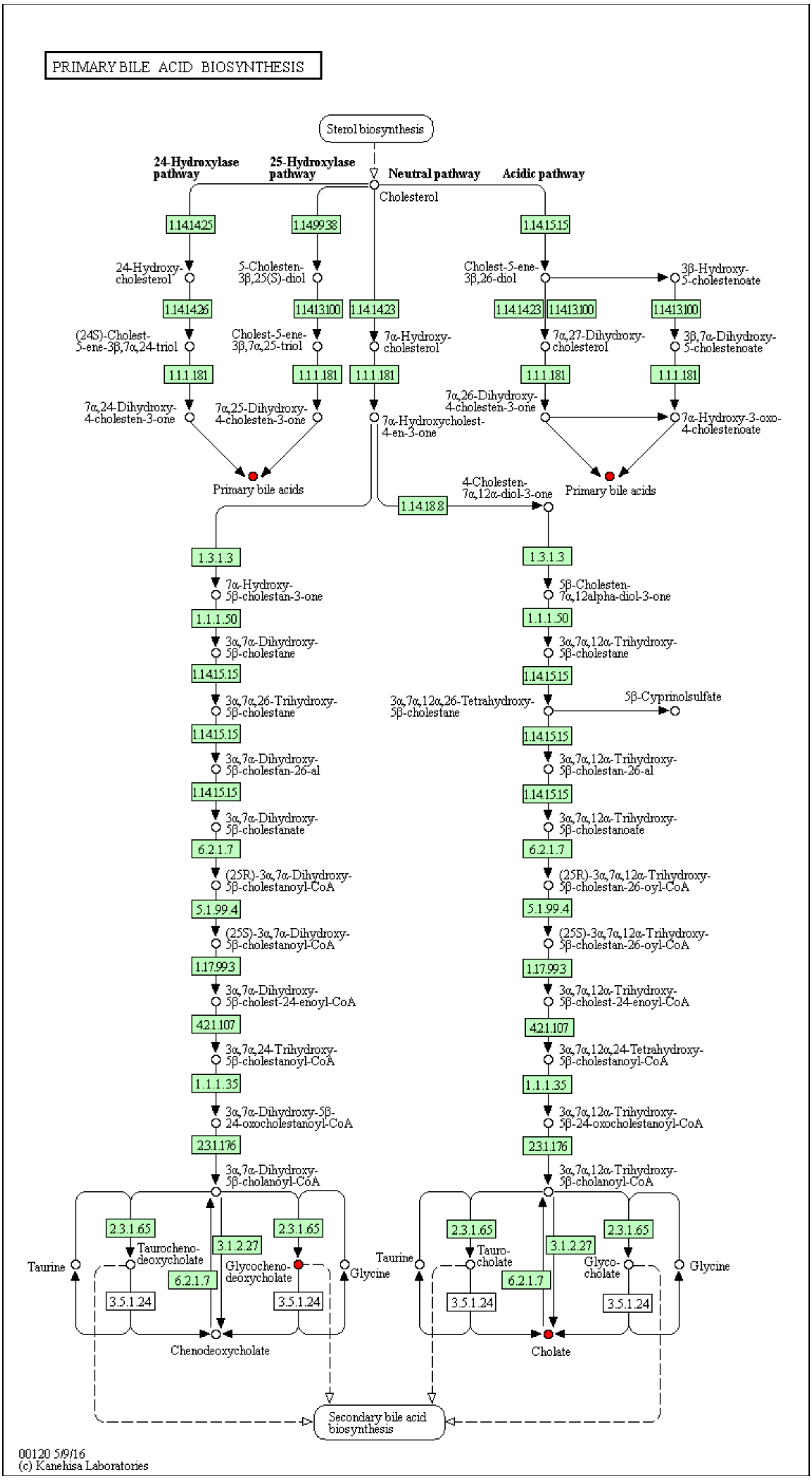

PRIMARY BILE ACID BIOSYNTHESIS
Sterol biosynthesis
24-Hydroxylase pathway
25-Hydroxylase pathway
Neutral pathway
Acidic pathway
Cholesterol
1.14.14.25
1.14.99.38
1.14.15.15
24-Hydroxy-cholesterol
5-Cholesten-3β,25(S)-diol
Cholest-5-ene-3β,26-diol
3β-Hydroxy-5-cholestenoate
1.14.14.26
1.14.13.100
1.14.14.23
1.14.14.23
1.14.13.100
1.14.13.100
(24S)-Cholest-5-ene-3β,7α,24-triol
Cholest-5-ene-3β,7α,25-triol
7α-Hydroxy-cholesterol
7α,27-Dihydroxy-cholesterol
3β,7α-Dihydroxy-5-cholestenoate
1.1.1.181
7α,24-Dihydroxy-4-cholesten-3-one
7α,25-Dihydroxy-4-cholesten-3-one
7α-Hydroxycholest-4-en-3-one
7α,26-Dihydroxy-4-cholesten-3-one
7α-Hydroxy-3-oxo-4-cholestenoate
Primary bile acids
Primary bile acids
1.14.18.8
4-Cholesten-7α,12α-diol-3-one
1.3.1.3
7α-Hydroxy-5β-cholestan-3-one
5β-Cholesten-7α,12alpha-diol-3-one
1.1.1.50
3α,7α-Dihydroxy-5β-cholestane
3α,7α,12α-Trihydroxy-5β-cholestane
1.14.15.15
3α,7α,26-Trihydroxy-5β-cholestane
3α,7α,12α,26-Tetrahydroxy-5β-cholestane
5β-Cyprinolsulfate
3α,7α-Dihydroxy-5β-cholestan-26-al
3α,7α,12α-Trihydroxy-5β-cholestan-26-al
3α,7α-Dihydroxy-5β-cholestanate
3α,7α,12α-Trihydroxy-5β-cholestanoate
6.2.1.7
(25R)-3α,7α-Dihydroxy-5β-cholestanoyl-CoA
(25R)-3α,7α,12α-Trihydroxy-5β-cholestan-26-oyl-CoA
5.1.99.4
(25S)-3α,7α-Dihydroxy-5β-cholestanoyl-CoA
(25S)-3α,7α,12α-Trihydroxy-5β-cholestan-26-oyl-CoA
1.17.99.3
3α,7α-Dihydroxy-5β-cholest-24-enoyl-CoA
3α,7α,12α-Trihydroxy-5β-cholest-24-enoyl-CoA
4.2.1.107
3α,7α,24-Trihydroxy-5β-cholestanoyl-CoA
3α,7α,12α,24-Tetrahydroxy-5β-cholestanoyl-CoA
1.1.1.35
3α,7α-Dihydroxy-5β-24-oxocholestanoyl-CoA
3α,7α,12α-Trihydroxy-5β-24-oxocholestanoyl-CoA
2.3.1.176
3α,7α-Dihydroxy-5β-cholanoyl-CoA
3α,7α,12α-Trihydroxy-5β-cholanoyl-CoA
2.3.1.65
3.1.2.27
Taurine
Taurochenodeoxycholate
Glycochenodeoxycholate
Glycine
Tauro-cholate
Glyco-cholate
3.5.1.24
Chenodeoxycholate
Cholate
Secondary bile acid biosynthesis
00120 5/9/16
(c) Kanehisa Laboratories

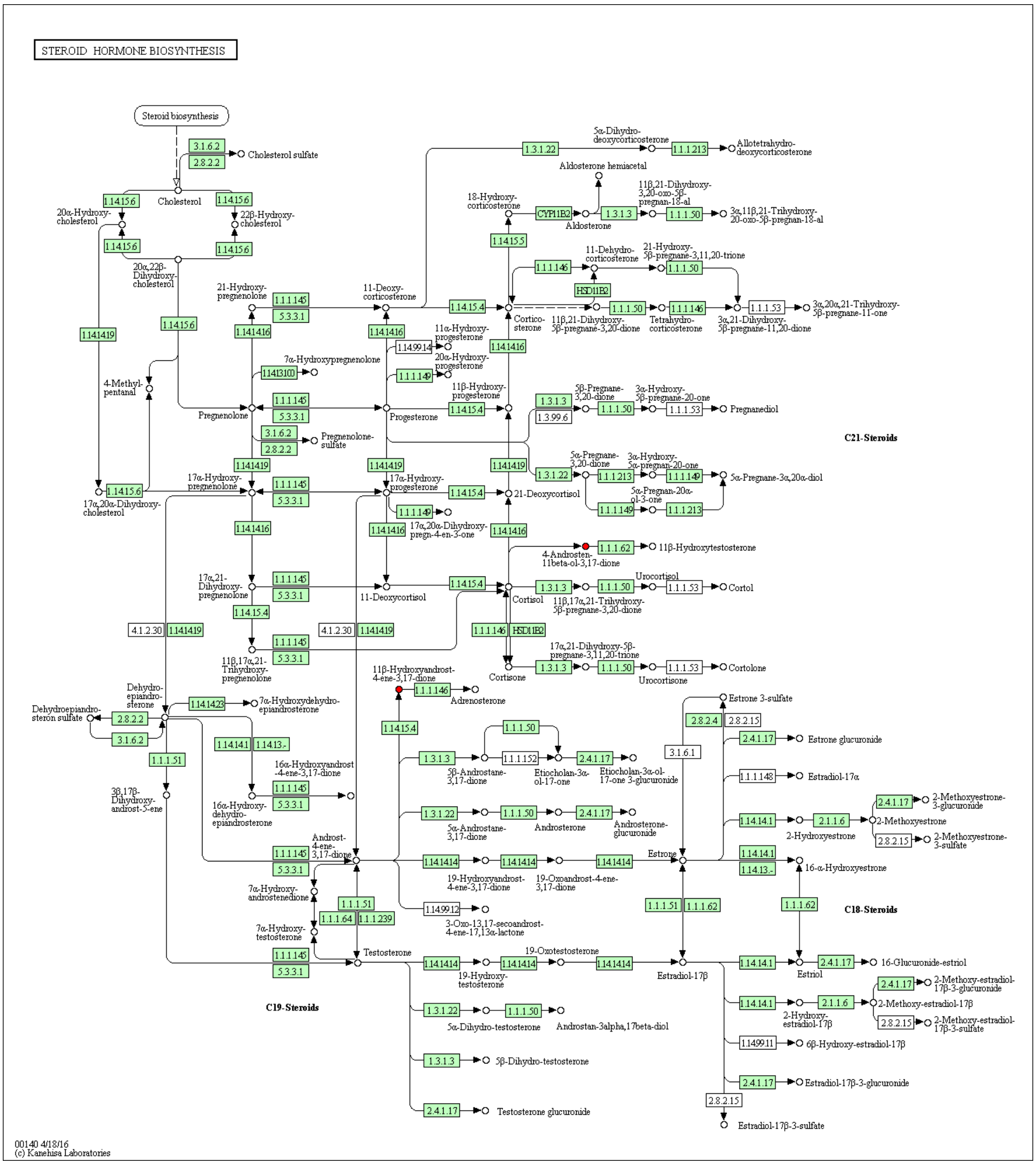
STEROID HORMONE BIOSYNTHESIS
Steroid biosynthesis
Cholesterol sulfate
Cholesterol
20α-Hydroxy-cholesterol
22β-Hydroxy-cholesterol
20α,22β-Dihydroxy-cholesterol
4-Methyl-pentanal
17α,20α-Dihydroxy-cholesterol
21-Hydroxy-pregnenolone
7α-Hydroxypregnenolone
Pregnenolone
Pregnenolone-sulfate
17α-Hydroxy-pregnenolone
17α,21-Dihydroxy-pregnenolone
11β,17α,21-Trihydroxy-pregnenolone
11-Deoxy-corticosterone
11α-Hydroxy-progesterone
20α-Hydroxy-progesterone
Progesterone
11β-Hydroxy-progesterone
17α-Hydroxy-progesterone
17α,20α-Dihydroxy-pregn-4-en-3-one
11-Deoxycortisol
21-Deoxycortisol
18-Hydroxy-corticosterone
Aldosterone hemiacetal
Aldosterone
Cortico-sterone
11-Dehydro-corticosterone
5α-Dihydro-deoxycorticosterone
Allotetrahydro-deoxycorticosterone
11β,21-Dihydroxy-3,20-oxo-5β-pregnan-18-al
3α,11β,21-Trihydroxy-20-oxo-5β-pregnan-18-al
21-Hydroxy-5β-pregnane-3,11,20-trione
11β,21-Dihydroxy-5β-pregnane-3,20-dione
Tetrahydro-corticosterone
3α,21-Dihydroxy-5β-pregnane-11,20-dione
3α,20α,21-Trihydroxy-5β-pregnane-11-one
5β-Pregnane-3,20-dione
3α-Hydroxy-5β-pregnane-20-one
Pregnanediol
C21-Steroids
5α-Pregnane-3,20-dione
3α-Hydroxy-5α-pregnan-20-one
5α-Pregnane-3α,20α-diol
5α-Pregnan-20α-ol-3-one
4-Androsten-11beta-ol-3,17-dione
11β-Hydroxytestosterone
Cortisol
Urocortisol
Cortol
11β,17α,21-Trihydroxy-5β-pregnane-3,20-dione
Cortisone
17α,21-Dihydroxy-5β-pregnane-3,11,20-trione
Urocortisone
Cortolone
11β-Hydroxyandrost-4-ene-3,17-dione
Adrenosterone
Dehydro-epiandro-sterone
Dehydroepiandro-steron sulfate
7α-Hydroxydehydro-epiandrosterone
3β,17β-Dihydroxy-androst-5-ene
16α-Hydroxyandrost-4-ene-3,17-dione
16α-Hydroxy-dehydro-epiandrosterone
5β-Androstane-3,17-dione
Etiocholan-3α-ol-17-one
Etiocholan-3α-ol-17-one 3-glucuronide
5α-Androstane-3,17-dione
Androsterone
Androsterone-glucuronide
Androst-4-ene-3,17-dione
7α-Hydroxy-androstenedione
7α-Hydroxy-testosterone
19-Hydroxyandrost-4-ene-3,17-dione
19-Oxoandrost-4-ene-3,17-dione
3-Oxo-13,17-secoandrost-4-ene-17,13α-lactone
Testosterone
19-Hydroxy-testosterone
19-Oxotestosterone
5α-Dihydro-testosterone
Androstan-3alpha,17beta-diol
5β-Dihydro-testosterone
Testosterone glucuronide
C19-Steroids
Estrone 3-sulfate
Estrone glucuronide
Estradiol-17α
2-Hydroxyestrone
2-Methoxyestrone
2-Methoxyestrone-3-glucuronide
2-Methoxyestrone-3-sulfate
Estrone
16-α-Hydroxyestrone
C18-Steroids
Estradiol-17β
Estriol
16-Glucuronide-estriol
2-Hydroxy-estradiol-17β
2-Methoxy-estradiol-17β
2-Methoxy-estradiol-17β-3-glucuronide
2-Methoxy-estradiol-17β-3-sulfate
6β-Hydroxy-estradiol-17β
Estradiol-17β-3-glucuronide
Estradiol-17β-3-sulfate
00140 4/18/16
(c) Kanehisa Laboratories

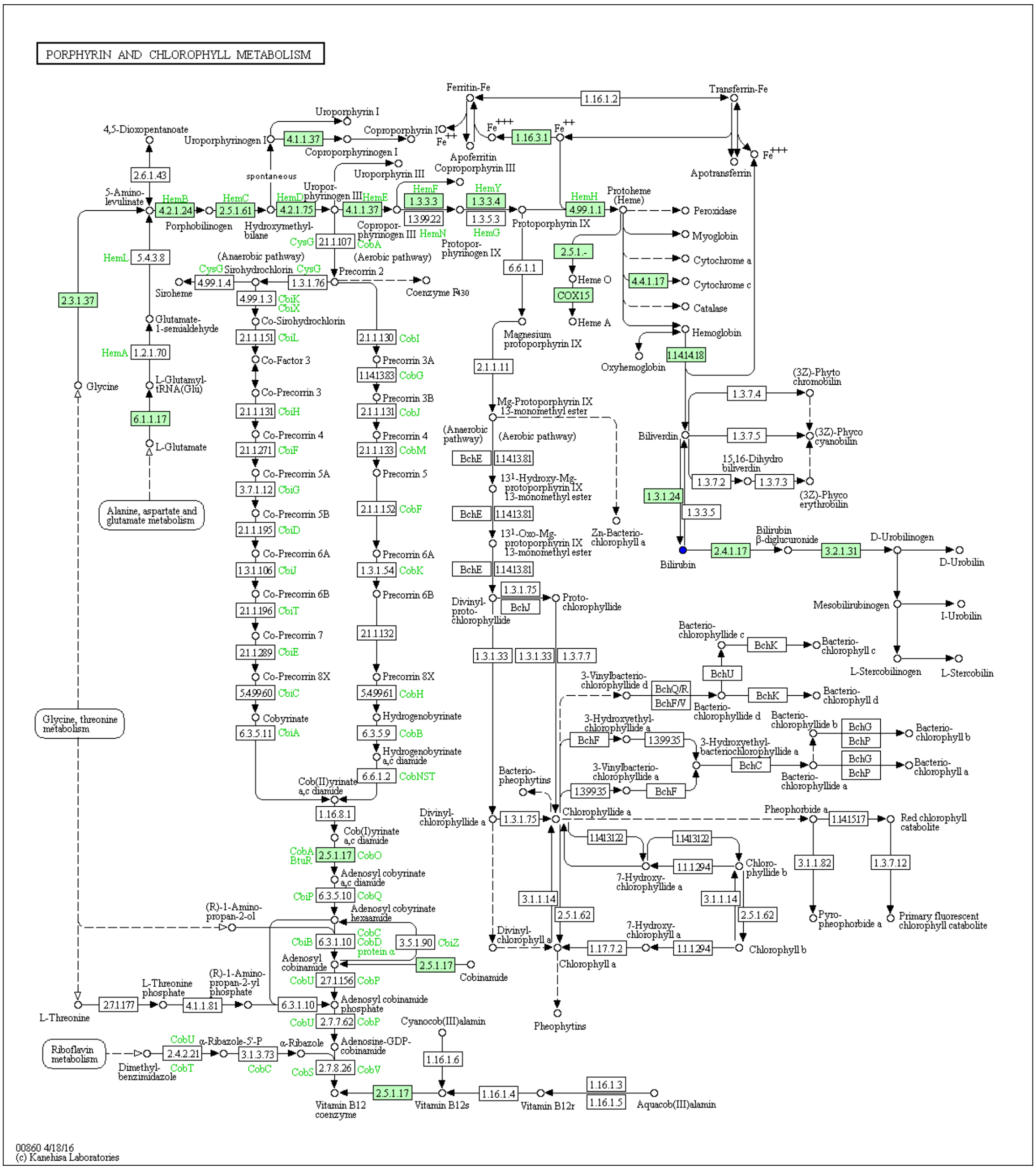

PORPHYRIN AND CHLOROPHYLL METABOLISM
Ferritin-Fe
1.16.1.2
Transferrin-Fe
Uroporphyrin I
4,5-Dioxopentanoate
Uroporphyrinogen I
4.1.1.37
Coproporphyrin I
Fe++
Fe+++
1.16.3.1
Coproporphyrinogen I
Apoferritin
Apotransferrin
spontaneous
Uroporphyrin III
Coproporphyrin III
2.6.1.43
5-Amino-levulinate
HemB
HemC
HemD
Uropor-phyrinogen III
HemE
HemF
HemY
HemH
Protoheme (Heme)
4.2.1.24
2.5.1.61
4.2.1.75
1.3.3.3
1.3.3.4
4.99.1.1
Peroxidase
Porphobilinogen
Hydroxymethyl-bilane
Copropor-phyrinogen III
1.3.99.22
1.3.5.3
Protoporphyrin IX
Myoglobin
HemN
HemG
Protopor-phyrinogen IX
CysG
2.1.1.107
CobA
2.5.1.-
Cytochrome a
HemL
5.4.3.8
(Anaerobic pathway)
(Aerobic pathway)
CysG Sirohydrochlorin CysG
Precorrin 2
6.6.1.1
Heme O
4.4.1.17
Cytochrome c
4.99.1.4
1.3.1.76
Coenzyme F430
Siroheme
COX15
Catalase
2.3.1.37
4.99.1.3
CbiK
CbiX
Glutamate-1-semialdehyde
Co-Sirohydrochlorin
Heme A
Hemoglobin
2.1.1.151
CbiL
2.1.1.130
CbiI
Magnesium protoporphyrin IX
HemA
1.2.1.70
Co-Factor 3
Precorrin 3A
1.14.14.18
Oxyhemoglobin
Glycine
L-Glutamyl-tRNA(Glu)
1.14.13.83
CobG
2.1.1.11
(3Z)-Phyto chromobilin
Co-Precorrin 3
Precorrin 3B
1.3.7.4
2.1.1.131
CbiH
2.1.1.131
CobJ
Mg-Protoporphyrin IX 13-monomethyl ester
6.1.1.17
Co-Precorrin 4
Precorrin 4
(Anaerobic pathway)
(Aerobic pathway)
Biliverdin
1.3.7.5
(3Z)-Phyco cyanobilin
L-Glutamate
2.1.1.271
CbiF
2.1.1.133
CobM
BchE
1.14.13.81
15,16-Dihydro biliverdin
Co-Precorrin 5A
Precorrin 5
1.3.7.2
1.3.7.3
3.7.1.12
CbiG
13¹-Hydroxy-Mg-protoporphyrin IX 13-monomethyl ester
1.3.1.24
(3Z)-Phyco erythrobilin
Alanine, aspartate and glutamate metabolism
Co-Precorrin 5B
2.1.1.152
CobF
BchE
1.14.13.81
1.3.3.5
2.1.1.195
CbiD
13¹-Oxo-Mg-protoporphyrin IX 13-monomethyl ester
Zn-Bacterio-chlorophyll a
Bilirubin β-diglucuronide
D-Urobilinogen
Co-Precorrin 6A
Precorrin 6A
2.4.1.17
3.2.1.31
1.3.1.106
CbiJ
1.3.1.54
CobK
BchE
1.14.13.81
Bilirubin
D-Urobilin
Co-Precorrin 6B
Precorrin 6B
1.3.1.75
Divinyl-proto-chlorophyllide
BchJ
Proto-chlorophyllide
2.1.1.196
CbiT
Mesobilirubinogen
I-Urobilin
Co-Precorrin 7
2.1.1.132
Bacterio-chlorophyllide c
BchK
Bacterio-chlorophyll c
2.1.1.289
CbiE
1.3.1.33
1.3.1.33
1.3.7.7
L-Stercobilinogen
L-Stercobilin
Co-Precorrin 8X
Precorrin 8X
BchU
5.4.99.60
CbiC
5.4.99.61
CobH
3-Vinylbacterio-chlorophyllide d
BchQ/R
BchK
Bacterio-chlorophyll d
Glycine, threonine metabolism
Cobyrinate
Hydrogenobyrinate
BchF/V
Bacterio-chlorophyllide d
3-Hydroxyethyl-chlorophyllide a
Bacterio-chlorophyllide b
BchG
Bacterio-chlorophyll b
6.3.5.11
CbiA
6.3.5.9
CobB
BchF
1.3.99.35
BchP
Hydrogenobyrinate a,c diamide
3-Hydroxyethyl-bacteriochlorophyllide a
BchG
Bacterio-chlorophyll a
Cob(II)yrinate a,c diamide
6.6.1.2
CobNST
Bacterio-pheophytins
BchC
BchP
3-Vinylbacterio-chlorophyllide a
1.3.99.35
BchF
Bacterio-chlorophyllide a
1.16.8.1
Divinyl-chlorophyllide a
1.3.1.75
Chlorophyllide a
Pheophorbide a
1.14.15.17
Red chlorophyll catabolite
Cob(I)yrinate a,c diamide
1.14.13.122
1.14.13.122
CobA
BtuR
2.5.1.17
CobO
1.1.1.294
Chloro-phyllide b
3.1.1.82
1.3.7.12
Adenosyl cobyrinate a,c diamide
7-Hydroxy-chlorophyllide a
CbiP
6.3.5.10
CobQ
3.1.1.14
3.1.1.14
Adenosyl cobyrinate hexaamide
2.5.1.62
2.5.1.62
Pyro-pheophorbide a
Primary fluorescent chlorophyll catabolite
(R)-1-Amino-propan-2-ol
7-Hydroxy-chlorophyll a
CbiB
6.3.1.10
CobC
CobD
protein α
3.5.1.90
CbiZ
Divinyl-chlorophyll a
1.17.7.2
1.1.1.294
Chlorophyll b
Adenosyl cobinamide
2.5.1.17
Cobinamide
Chlorophyll a
L-Threonine phosphate
(R)-1-Amino-propan-2-yl phosphate
CobU
2.7.1.156
CobP
2.7.1.177
4.1.1.81
6.3.1.10
Adenosyl cobinamide phosphate
L-Threonine
CobU
2.7.7.62
CobP
Cyanocob(III)alamin
Pheophytins
Riboflavin metabolism
CobU
α-Ribazole-5'-P
α-Ribazole
Adenosine-GDP-cobinamide
2.4.2.21
3.1.3.73
1.16.1.6
Dimethyl-benzimidazole
CobT
CobC
CobS
2.7.8.26
CobV
1.16.1.3
2.5.1.17
1.16.1.4
1.16.1.5
Vitamin B12 coenzyme
Vitamin B12s
Vitamin B12r
Aquacob(III)alamin
00860 4/18/16
(c) Kanehisa Laboratories
