# Supplementary figures and images for: The Metabolomic Characterization of Different Types of Coronary Atherosclerotic Heart Disease in Male
Source: Cardiol Res Pract. 2022 Jul 12;2022:6491129. doi: 10.1155/2022/6491129 (PMC9296306; doi:10.1155/2022/6491129)

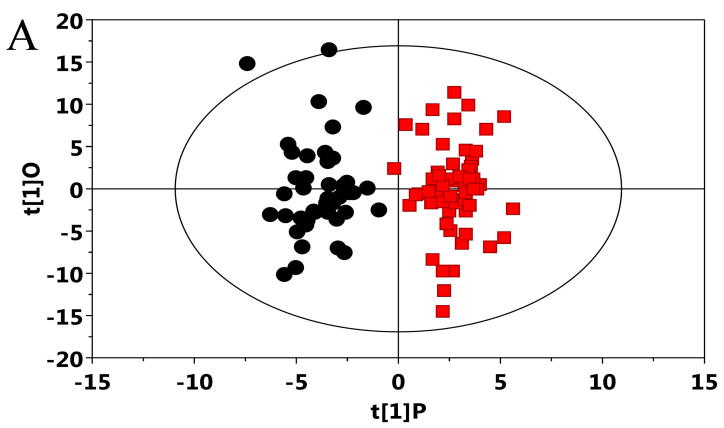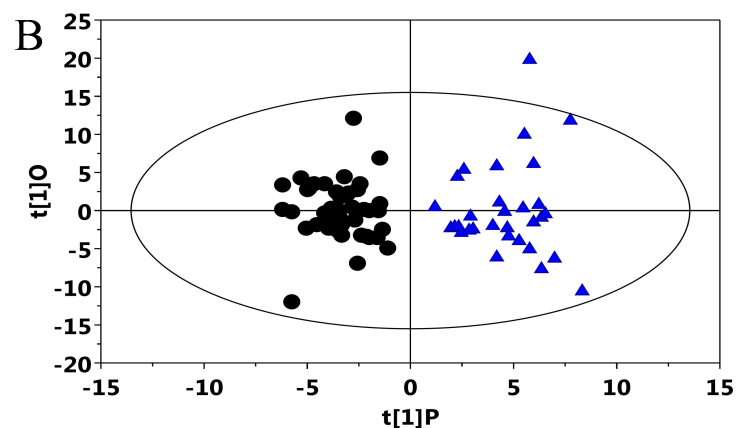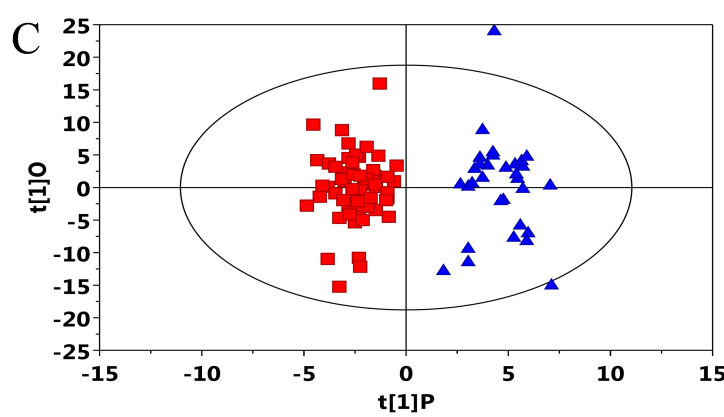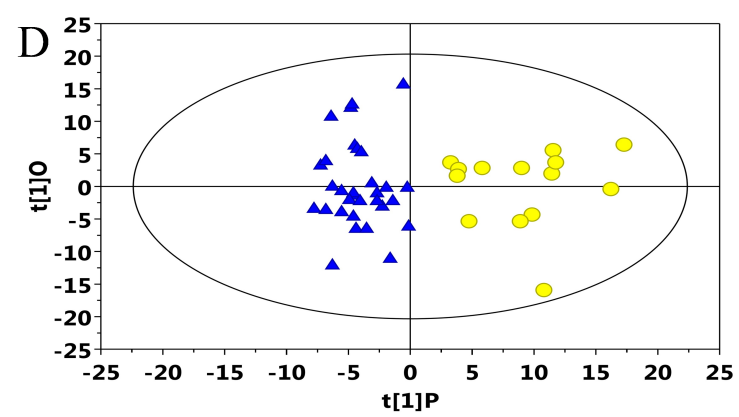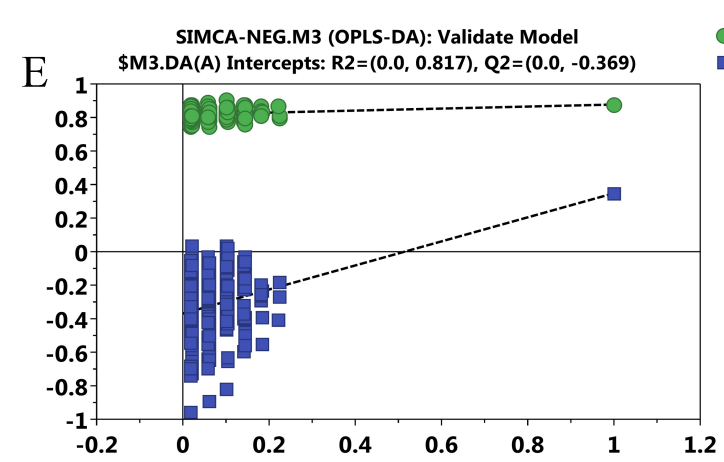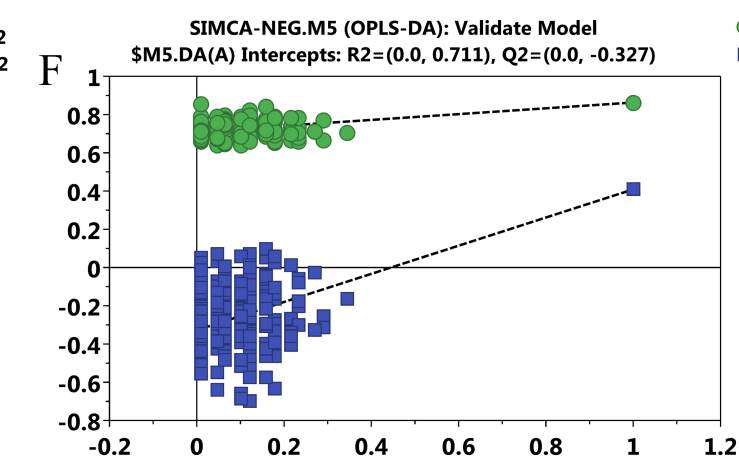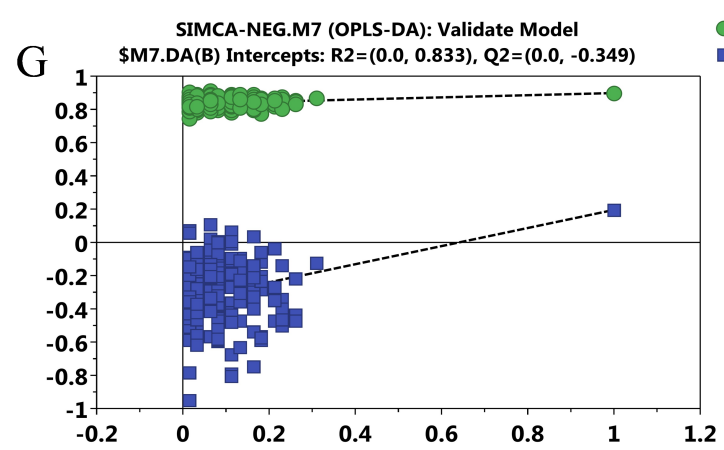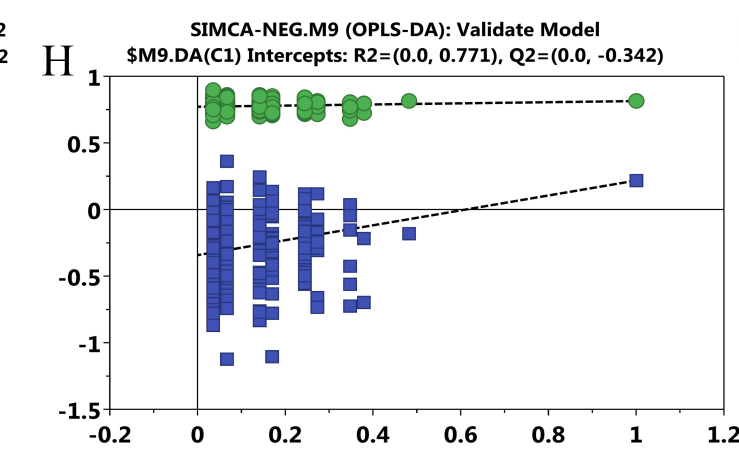

Supplement: Supplementary Materials — Supplementary Material 1: metabolic pathways analysis based on the KEGG pathway database in all experimental groups. Figure S1: Figure S1(a)-S1D. Score plot of OPLS-DA model obtained from experimental groups (NEG). black: HC, red: SA, blue: PR-ACS, yellow: PO-ACS. Figure S1(e)- S1H, the validated model of OPLS-DA. 200 times were performed, and the resulting R2 and Q2 values were plotted. (Green triangle): R2; (blue square): Q2. The green line represents the regression line for R2 and the blue line for Q2. Figure S2: loading plot of OPLS-DA model obtained from experimental groups (NEG). A: HC versus SCAD, B: HC versus PR-ACS, C: SCAD versus PR-ACS, D: PR-ACS versus PO-ACS. Figure S3: pathway analysis of experiment group. The larger the circle meant the greater the influence of topology analysis; the redder the color meant the smaller the p value, and vice versa. A: HC versus SCAD, B: HC versus PR-ACS, C: SCAD versus PR-ACS, D: PR-ACS versus PO-ACS (NEG). [file 6491129.f1.zip › 6491129.f1/Fig.S1.pdf]

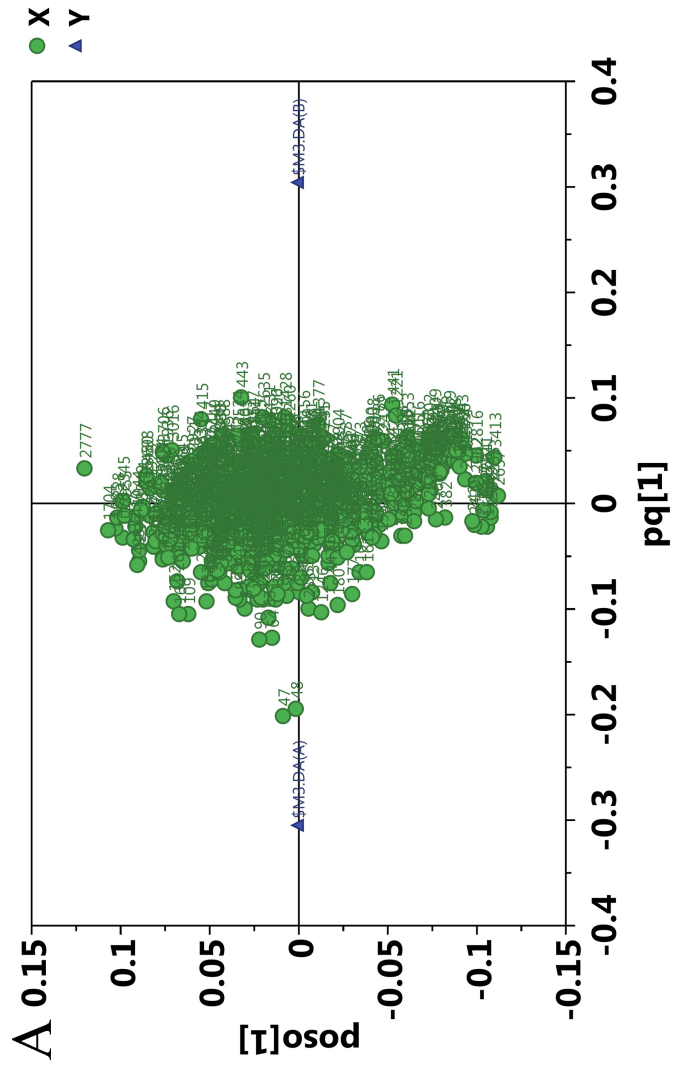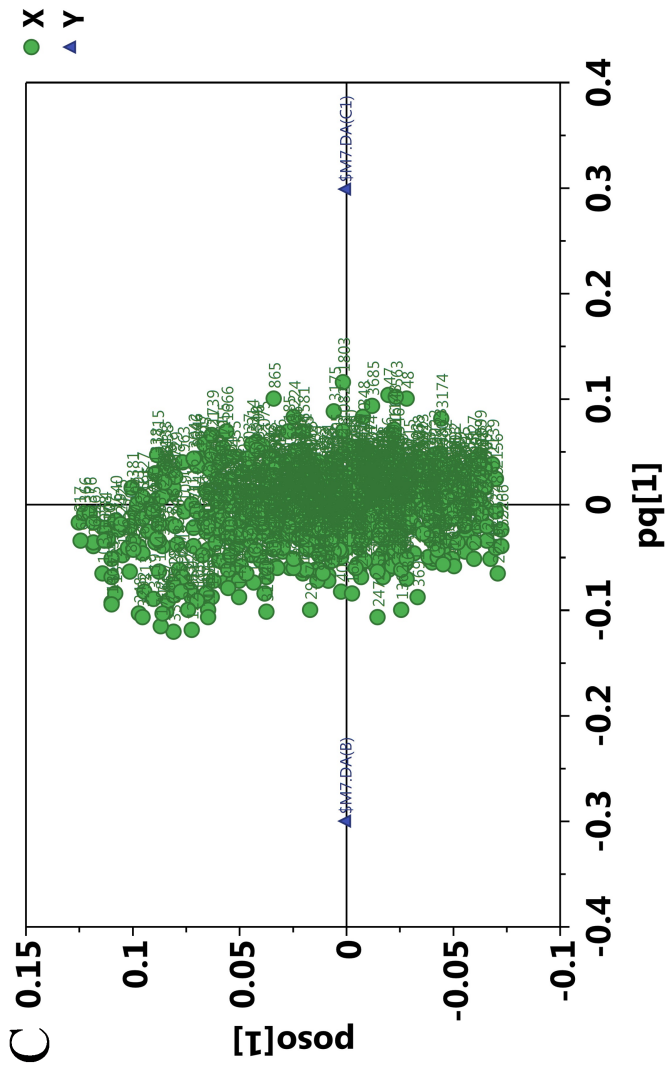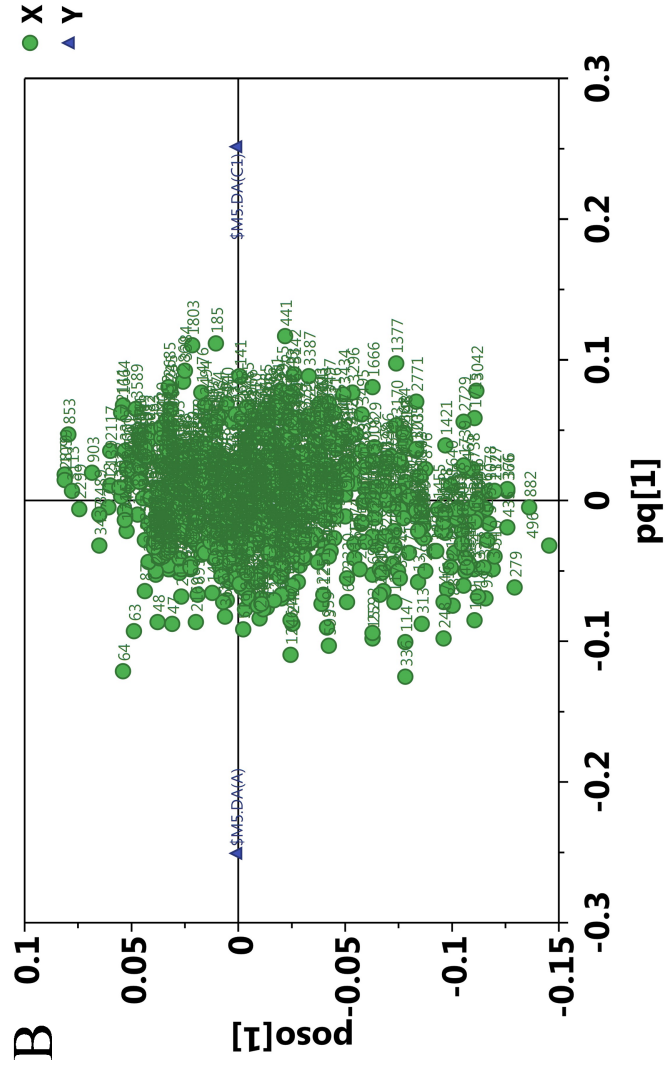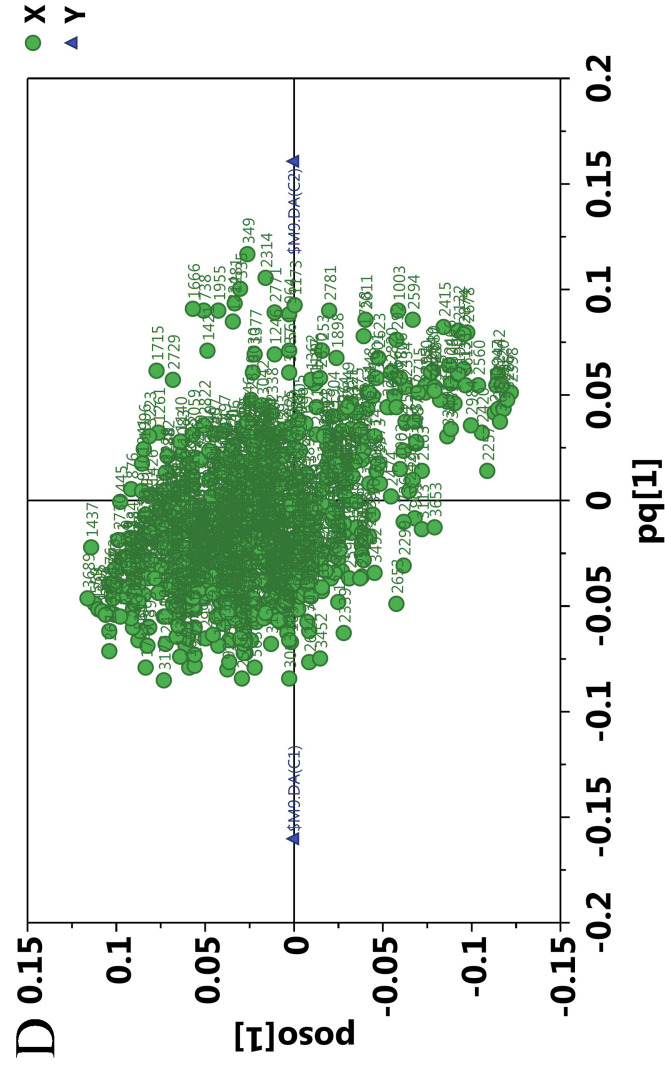

Supplement: Supplementary Materials — Supplementary Material 1: metabolic pathways analysis based on the KEGG pathway database in all experimental groups. Figure S1: Figure S1(a)-S1D. Score plot of OPLS-DA model obtained from experimental groups (NEG). black: HC, red: SA, blue: PR-ACS, yellow: PO-ACS. Figure S1(e)- S1H, the validated model of OPLS-DA. 200 times were performed, and the resulting R2 and Q2 values were plotted. (Green triangle): R2; (blue square): Q2. The green line represents the regression line for R2 and the blue line for Q2. Figure S2: loading plot of OPLS-DA model obtained from experimental groups (NEG). A: HC versus SCAD, B: HC versus PR-ACS, C: SCAD versus PR-ACS, D: PR-ACS versus PO-ACS. Figure S3: pathway analysis of experiment group. The larger the circle meant the greater the influence of topology analysis; the redder the color meant the smaller the p value, and vice versa. A: HC versus SCAD, B: HC versus PR-ACS, C: SCAD versus PR-ACS, D: PR-ACS versus PO-ACS (NEG). [file 6491129.f1.zip › 6491129.f1/Fig.S2.pdf]

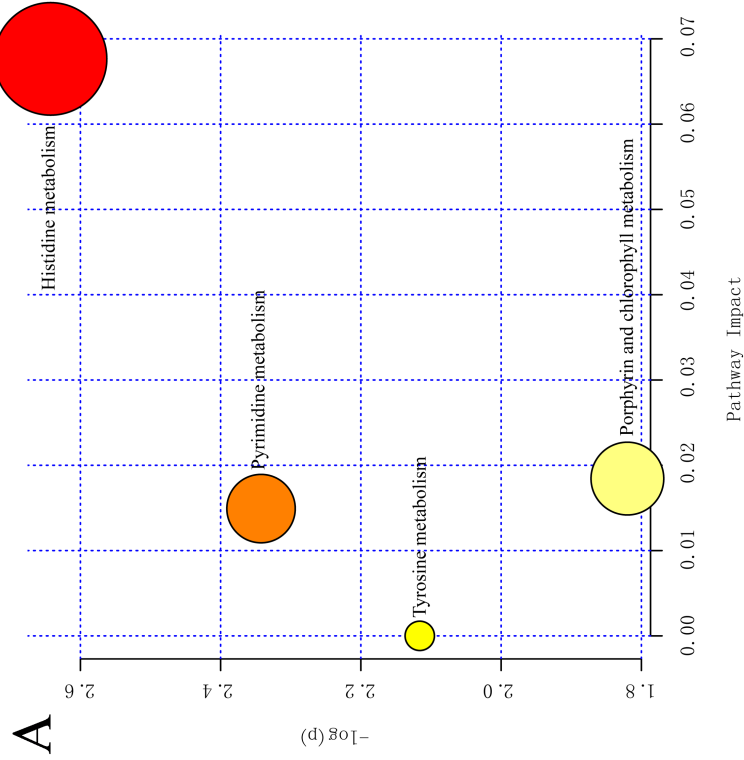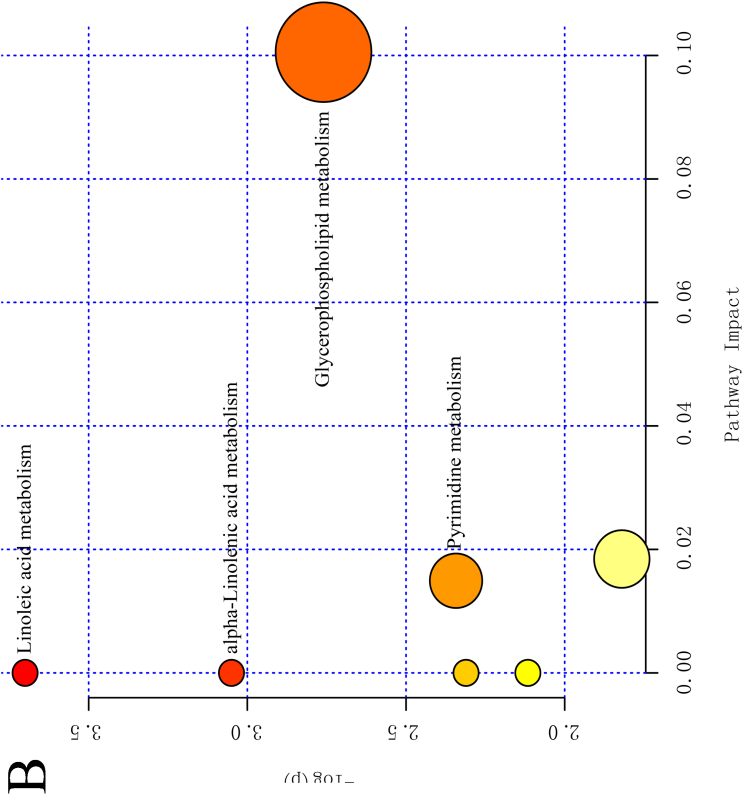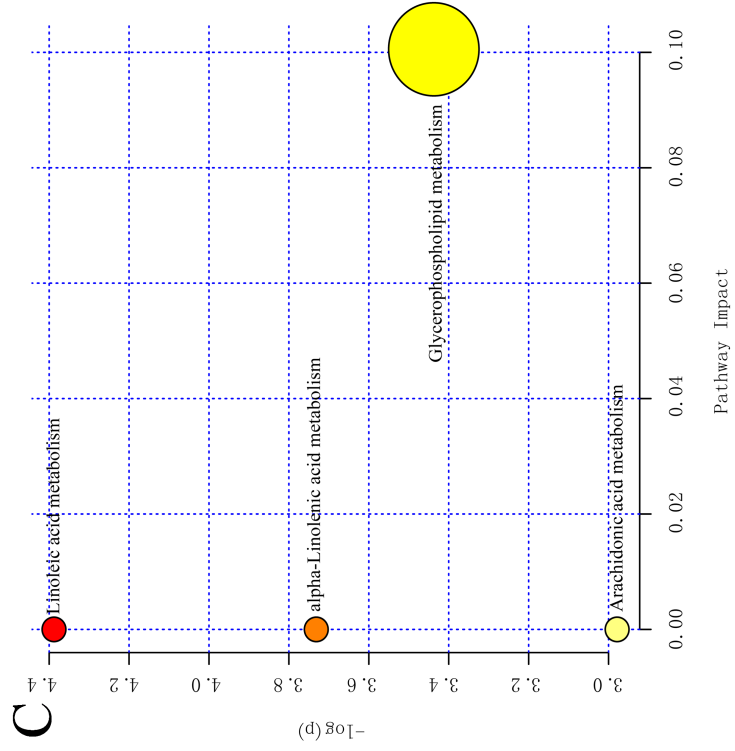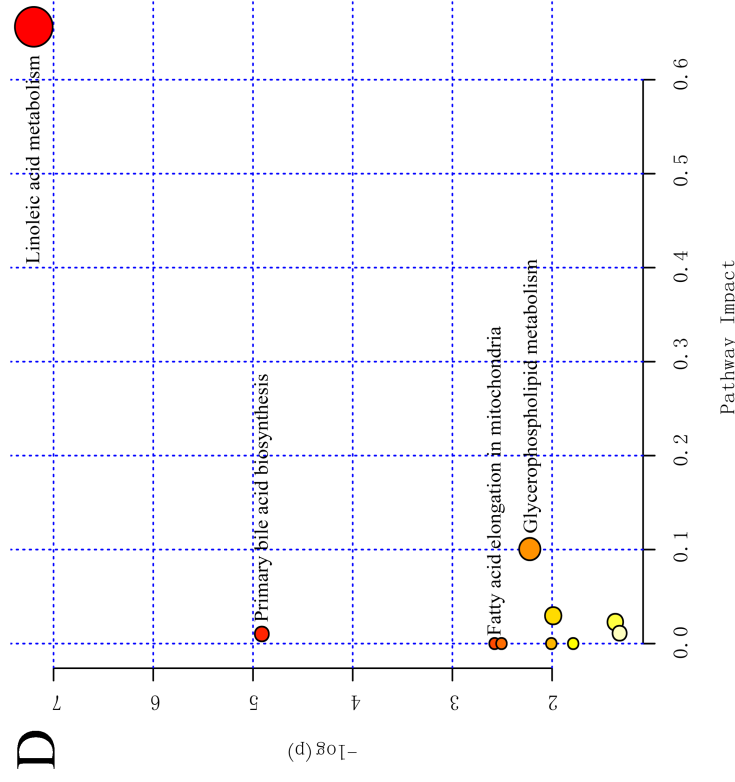

Supplement: Supplementary Materials — Supplementary Material 1: metabolic pathways analysis based on the KEGG pathway database in all experimental groups. Figure S1: Figure S1(a)-S1D. Score plot of OPLS-DA model obtained from experimental groups (NEG). black: HC, red: SA, blue: PR-ACS, yellow: PO-ACS. Figure S1(e)- S1H, the validated model of OPLS-DA. 200 times were performed, and the resulting R2 and Q2 values were plotted. (Green triangle): R2; (blue square): Q2. The green line represents the regression line for R2 and the blue line for Q2. Figure S2: loading plot of OPLS-DA model obtained from experimental groups (NEG). A: HC versus SCAD, B: HC versus PR-ACS, C: SCAD versus PR-ACS, D: PR-ACS versus PO-ACS. Figure S3: pathway analysis of experiment group. The larger the circle meant the greater the influence of topology analysis; the redder the color meant the smaller the p value, and vice versa. A: HC versus SCAD, B: HC versus PR-ACS, C: SCAD versus PR-ACS, D: PR-ACS versus PO-ACS (NEG). [file 6491129.f1.zip › 6491129.f1/Fig.S3.pdf]
